# Formal total syntheses of classic natural product target molecules via palladium-catalyzed enantioselective alkylation

**DOI:** 10.3762/bjoc.10.261

**Published:** 2014-10-28

**Authors:** Yiyang Liu, Marc Liniger, Ryan M McFadden, Jenny L Roizen, Jacquie Malette, Corey M Reeves, Douglas C Behenna, Masaki Seto, Jimin Kim, Justin T Mohr, Scott C Virgil, Brian M Stoltz

**Affiliations:** 1 Warren and Katharine Schlinger Laboratory of Chemistry and Chemical Engineering, Division of Chemistry and Chemical Engineering, California Institute of Technology, 1200 E. California Boulevard, Pasadena, CA, USA

**Keywords:** enantioselective alkylation, natural products, palladium

## Abstract

Pd-catalyzed enantioselective alkylation in conjunction with further synthetic elaboration enables the formal total syntheses of a number of “classic” natural product target molecules. This publication highlights recent methods for setting quaternary and tetrasubstituted tertiary carbon stereocenters to address the synthetic hurdles encountered over many decades across multiple compound classes spanning carbohydrate derivatives, terpenes, and alkaloids. These enantioselective methods will impact both academic and industrial settings, where the synthesis of stereogenic quaternary carbons is a continuing challenge.

## Introduction

**Scheme 1:**
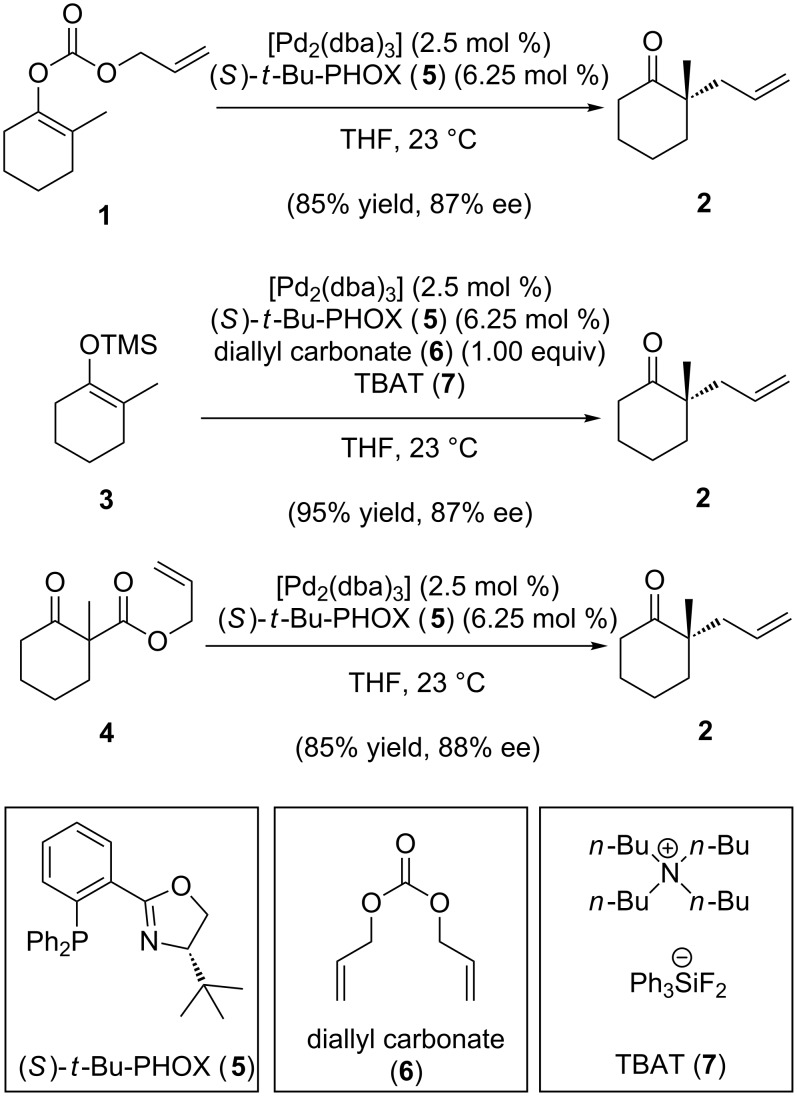
Three classes of Pd-catalyzed enantioselective allylic alkylations.

Catalytic enantioselective allylic alkylation has emerged as a powerful method for the construction of building blocks bearing quaternary carbon and fully substituted tertiary centers [1–2]. A recent addition developed by our laboratory is the allylic alkylation of nonstabilized enolate precursors to form α-quaternary carbonyl compounds (Scheme 1) [3]. Once the key stereocenter is set by this chemistry, further elaboration allows access to many bioactive small molecules. In our lab alone, this palladium-catalyzed alkylation has enabled the enantioselective total syntheses of dichroanone [4], elatol [5], cyanthiwigins [6–8], carissone [9], cassiol [10], chamigrenes [11], and liphagal [12]. Other labs have also utilized our method in natural product total synthesis [13–14]. Often, it is the case that a new technology that allows the synthesis of building blocks will open up new avenues to complex structures of long standing interest [15–16]. Herein we detail the application of this asymmetric chemistry in formal total syntheses of “classic” natural product targets across a range of compound families by strategic selection of allylic alkylation substrates and subsequent product transformations.

## Results and Discussion

### A) Thujopsene

**Figure 1:**
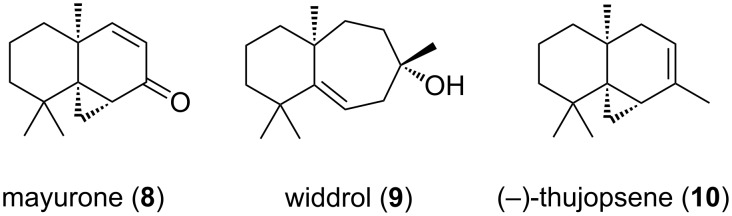
Selected natural products from *Thujopsis dolabrata*.

The Japanese hiba tree, *Thujopsis dolabrata* has been used for centuries as decoration and within traditional architecture [17]. The plant is a member of the order *Cupressaceae*, and its fragrant wood oil contains numerous sesquiterpenes including mayurone (**8**) [18–19], widdrol (**9**) [20], and (−)-thujopsene (**10**) (Figure 1) [21–22]. The wood oil is a potent dust mite deterrent; thus, in addition to its ornamental value, the hiba tree also provides and environmentally benign means of pest control [23–24].

(−)-Thujopsene (**10**) has attractive features to the synthetic chemist. Its tricyclo[5.4.0.0^1,3^]undecane skeleton contains three contiguous all-carbon quaternary centers, two of which are stereogenic. Being a hydrocarbon, (−)-thujopsene (**10**) has few natural handles for retrosynthetic analysis. Inspired by the complexity of this relatively small natural product, several total syntheses of racemic **10** have been reported [25–29] along with at least two enantioselective routes [30–32].

**Scheme 2:**
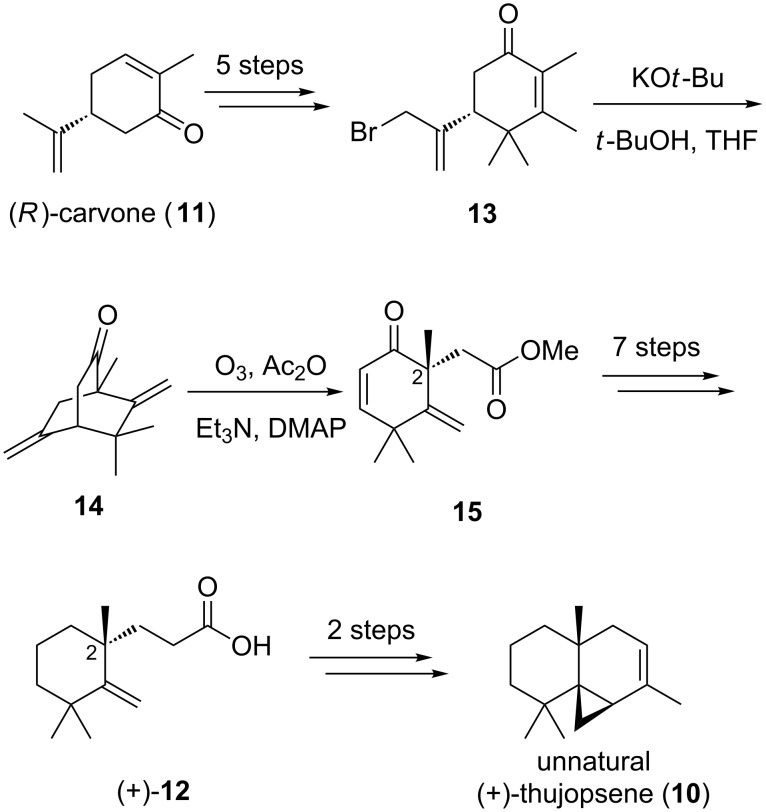
Srikrishna and Anebouselvy’s approach to (+)-thujopsene.

One enantiospecific total synthesis of (+)-thujopsene (**10**) by Srikrishna and Anebouselvy began with (*R*)-carvone (**11**) (Scheme 2) [33]. During the total synthesis, the authors prepared carboxylic acid (+)-**12** over a 14-step sequence. We planned to intercept the antipode of (+)-**12** using the palladium-catalyzed enantioselective alkylation chemistry described above.

**Scheme 3:**
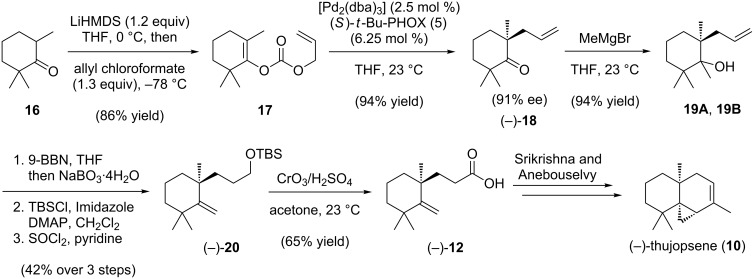
Formal total synthesis of (−)-thujopsene.

We commenced a formal total synthesis of (−)-thujopsene (**10**) with the goal of improved efficiency compared to the Srikrishna/Anebouselvy route and to use enantioselective palladium catalysis to install the initial stereocenters (Scheme 3). Treatment of **16** with LiHMDS in THF, followed by allyl chloroformate, furnished the known carbonate **17** in high yield [34]. This substrate smoothly undergoes palladium-catalyzed enantioselective decarboxylative allylation in the presence of (*S*)-*t*-Bu-PHOX (**5**), giving allyl ketone (−)-**18** in 94% yield and 91% ee [34]. Treatment of the ketone (−)-**18** with MeMgBr at 23 ºC provided a mixture of two diastereomeric alcohols **19A** and **19B** in 94% yield. Without separation, the diastereomers were rapidly carried through a three-step sequence of hydroboration/oxidation, terminal alcohol silylation, and tertiary alcohol dehydration, affording methylene cyclohexane (−)-**20**. Treatment of this silyl ether with Jones reagent simultaneously cleaved the silyl group and oxidized the resulting alcohol, furnishing carboxylic acid (−)-**12** in 65% yield. With this enantioenriched acid in hand, the formal total synthesis of (−)-thujopsene (**10**) is completed in only 9 steps from trimethylcyclohexanone **16**.

### B) Quinic acid

**Scheme 4:**
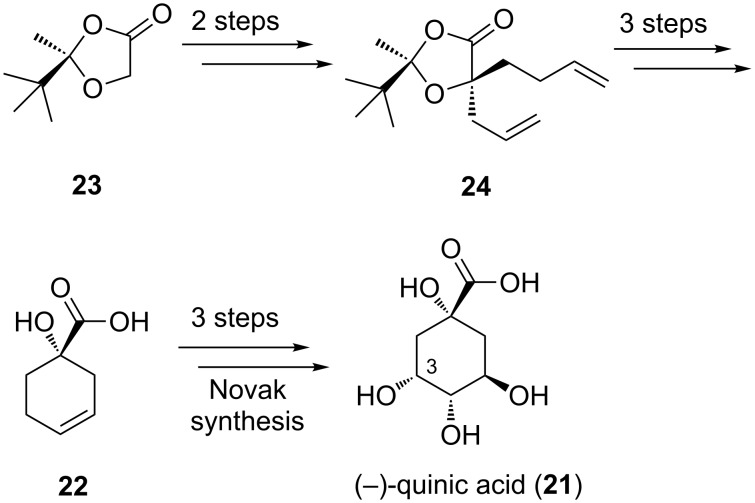
Renaud’s formal total synthesis of (−)-quinic acid.

(−)-Quinic acid (**21**) [35–36] serves as a useful chiral building block that has been employed in numerous syntheses [37], including our own syntheses of (+)- and (−)-dragmacidin F [38–41], and the initial commercial-scale synthesis of Tamiflu [42]. In Renaud’s formal total synthesis of (−)-quinic acid (**21**) [35], a key carboxylic acid **22** was accessed, intercepting Novak’s older synthesis of the natural product (Scheme 4) [36]. To begin, Renaud transformed the chiral glycolic acid ketal **23** (enantioenriched to 80% ee) to the more elaborate diene **24** via two diastereoselective alkylations. After a sequence of three reactions including removal of the pinacolone portion of the auxiliary, carboxylic acid **22** could be accessed. Novak’s synthesis applied a bromolactonization of **22** to build in the requisite syn relationship between the carboxylate group and the 3-hydroxy group, ultimately leading to quinic acid.

**Scheme 5:**
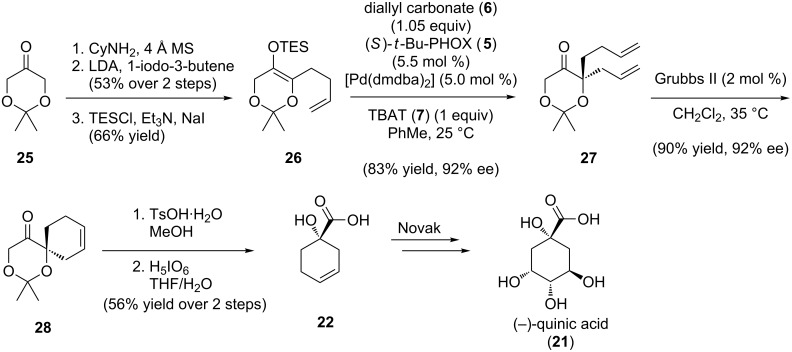
Formal total synthesis of (−)-quinic acid.

Unlike the allylic alkylations in Scheme 1, which form all-carbon stereocenters, we envisioned a unique modification of the silyl enol ether version to access nonracemic tertiary alcohols (Scheme 5) [43]. The planned modification would involve the use of dioxanone-derived substrates instead of the prototypical cycloalkanone-derived ones. To demonstrate this new technology in the context of formal total synthesis, we chose to intercept the acid **22** in the Renaud and Novak routes to quinic acid (**21**). Conversion of dioxanone **25** to a cyclohexylimine enabled alkylation via a metalloenamine. On acidic work-up, imine hydrolysis furnished an alkylated dioxanone in good yield. The targeted silyl enol ether **26** was prepared by thermodynamic silylation in 66% yield [43]. Optimal conversions and enantioselectivities were achieved from triethylsilyl enol ether **26** on exposure to [Pd(dmdba)_2_] (5 mol %), (*S*)-*t*-BuPHOX (**5**, 5.5 mol %), and diallyl carbonate (1.05 equiv) at 25 °C, in PhMe with an equivalent of Bu_4_NPh_3_SiF_2_ (TBAT) [43]. Recognizing that enantioenriched α,ω-dienes could be transformed into cycloalkenes with a stereocenter remote to the olefin [44], chiral diene **27** was submitted to ring closing metathesis to generate **28** in 90% yield and 92% ee [43]. Cyclohexene **28** readily undergoes acetonide cleavage and periodic acid oxidation to provide carboxylic acid (*S*)-**22** [43], completing the formal synthesis of (−)-quinic acid (**21**). Additionally, one could in principle also access the less commercially abundant antipode (+)-quinic acid (**21**) using the catalyst (*R*)-*t*-Bu-PHOX.

### C) Dysidiolide

**Scheme 6:**
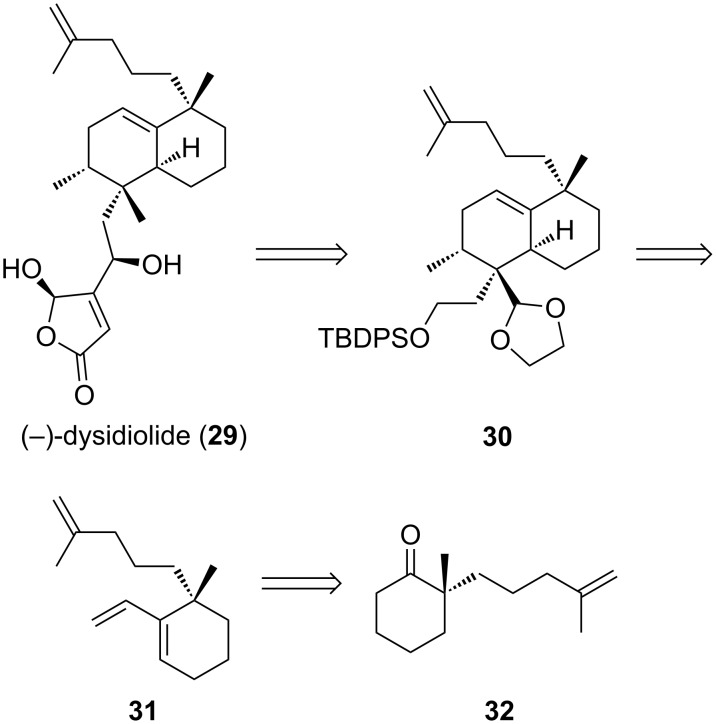
Danishefsky’s approach to (±)-dysidiolide.

Dysidiolide (**29**, Scheme 6) was isolated from the marine sponge *Dysidea etheria* and found to have inhibitory activity toward protein phosphatase cdc25, with an IC_50_ value of 9.4 µM [45]. This enzyme is a member of the protein family responsible for dephosphorylation of cyclin-dependent kinases [46]. Thus, inhibitors of cdc25 might allow for targeted cell-cycle disruption [45]. The relative stereochemistry of dysidiolide (**29**) was determined via single-crystal X-ray diffraction analysis, revealing a molecule with six stereocenters, two of which are quaternary carbons [45]. Several groups have reported total syntheses of this natural product [47–53], three of which are enantioselective [54–56].

In Danishefsky’s approach to racemic dysidiolide, the cyclohexene ring of **30** was installed via diastereoselective Diels–Alder reaction of a transient dioxolenium dienophile and chiral vinylcyclohexene **31** [48]. Triene **31** was prepared from α-quaternary ketone (±)-**32** in racemic form. We anticipated the interception of (−)-**32** [57] in Danishefsky’s route using enantioselective palladium-catalyzed allylic alkylation to set the quaternary stereocenter.

**Scheme 7:**
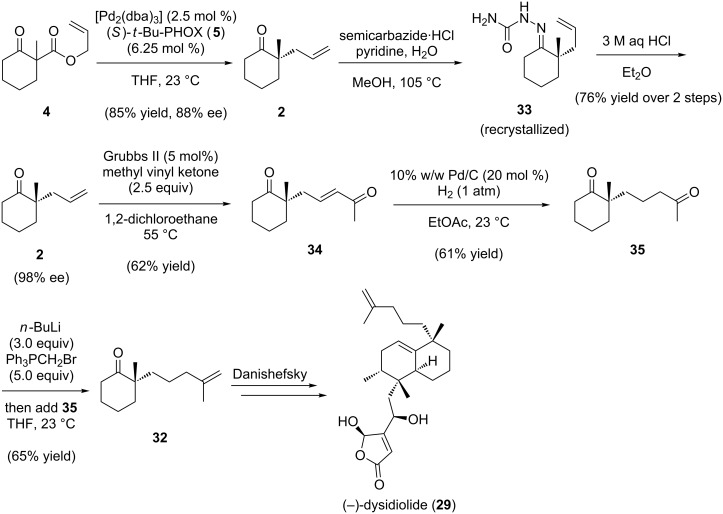
Formal total synthesis of (−)-dysidiolide.

The formal total synthesis of (−)-dysidiolide (**29**) commenced with known allyl β-ketoester **4** (Scheme 7), which was converted to 2-allyl-2-methylcyclohexanone (**2**) in 85% yield and 88% ee [58] with a catalytic amount of [Pd_2_(dba)_3_] and (*S*)-*t*-BuPHOX (**5**, Scheme 1). The allyl ketone was enriched to 98% ee via recrystallization of semicarbazone **33** [59]. Using the Grubbs 2^nd^ generation metathesis catalyst, allyl ketone (−)-**2** was crossed with methyl vinyl ketone in 62% yield [34]. Reduction of enone **34** was achieved in the presence of Pd/C with H_2_ in EtOAc to furnish diketone **35** [34]. Chemoselective Wittig mono-olefination of **35** provided ω-enone (−)-**32**, spectroscopically identical to the material in Danishefsky’s racemic synthesis. This formal synthesis shows the power of the enantioselective allylic alkylation to access formerly racemic constructs as single enantiomers; Danishefsky’s synthesis is now rendered enantioselective.

### D) Aspidospermine

**Scheme 8:**
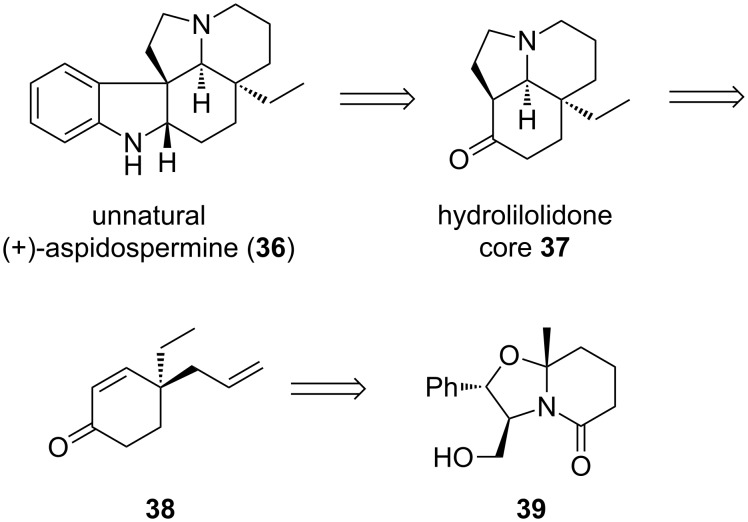
Meyers’ approach to unnatural (+)-aspidospermine.

The aspidosperma alkaloids have garnered much attention as beautiful targets for the synthetic chemist. Most of the 250-plus compounds in this class share a pentacyclic core, from the clinical anticancer therapeutics vincristine and vinblastine to the simpler aspidospermidine [60]. To address the challenging synthetic features of the aspidosperma alkaloids, many clever synthetic approaches have been reported [61–62]. One popular target in this family is aspidospermine (**36**, Scheme 8). Although its medicinal potency is inferior to other members of the class, this alkaloid has served as a proving ground for many synthetic chemists.

In 1989, Meyers reported an enantioselective synthesis of the (4a*S*,8a*R*,8*S*)-hydrolilolidone core **37** [63–64] present in aspidospermine (**36**), and thus a formal total synthesis of the alkaloid itself, intercepting Stork’s classic route [61]. One precursor described in the core synthesis is enone **38**, which bears the quaternary stereocenter of the natural product. Contrasting Meyers’ approach, which employed a chiral auxiliary as part of **39**, we thought a catalytic enantioselective alkylation strategy would be ideal for a formal total synthesis of natural (−)-aspidospermine (**36**) via the antipode of **38**.

**Scheme 9:**
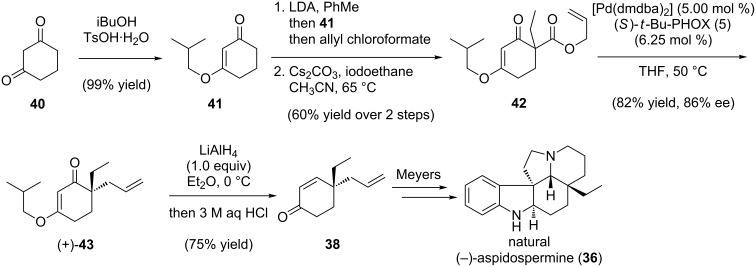
Formal total synthesis of (−)-aspidospermine.

The formal synthesis began with 1,3-cyclohexanedione (**40**), which was converted to isobutyl vinylogous ether **41** under acid promotion (Scheme 9) [65]. The β-ketoester **42** was prepared using a two-step sequence of acylation and alkylation, then treated with the (*S*)-*t*-Bu-PHOX catalyst system (with [Pd(dmdba)_2_]) to generate (+)-**43** in 86% ee. The challenge of installing the γ-stereocenter of the target **38** was addressed as follows: LiAlH_4_ treatment of (+)-**43** gave exclusive 1,2-reduction. When the crude product was hydrolyzed, β-elimination gave the desired enone **38**. The overall formal synthesis represents a rare example of enantioselective Stork–Danheiser chemistry [66–67].

### E) Rhazinilam

(−)-Rhazinilam (**44**) has been isolated from various plants including *Rhazya strica decaisne* [68], *Melodinus australis* [69], and *Kopsia singapurensis* [70]. Shortly after the first isolation, its structure was elucidated by single crystal X-ray diffraction analysis [71]. It features a tetracyclic scaffold with a nine-membered ring and an all-carbon quaternary stereocenter. This alkaloid is a microtubule-disrupting agent that displays similar cellular effects to paclitaxel [72–73]. Because of its biological activities and potential pharmaceutical use, many groups have pursued its total synthesis [74–77], including a number of enantioselective syntheses [78–82].

**Scheme 10:**
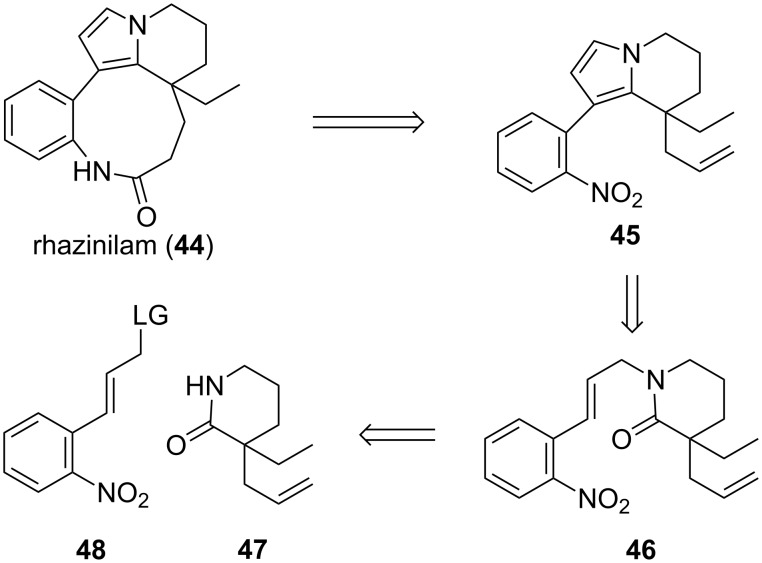
Magnus’ approach to (±)-rhazinilam.

In 2001, Magnus reported a total synthesis of rhazinilam in racemic form (Scheme 10) [83]. In their approach, the first retrosynthetic disconnection of the amide C–N bond in the nine-membered ring led to tricyclic compound **45**. The pyrrole ring of **45** was formed by intramolecular condensation of cinnamyl amide **46**, which is prepared via union of quaternary piperidinone **47** and cinnamyl electrophile **48**. We envisioned that our allylic alkylation of lactam enolates would furnish enantioenriched piperidinone **47**, and thus a single enantiomer of rhazinilam may be prepared.

**Scheme 11:**
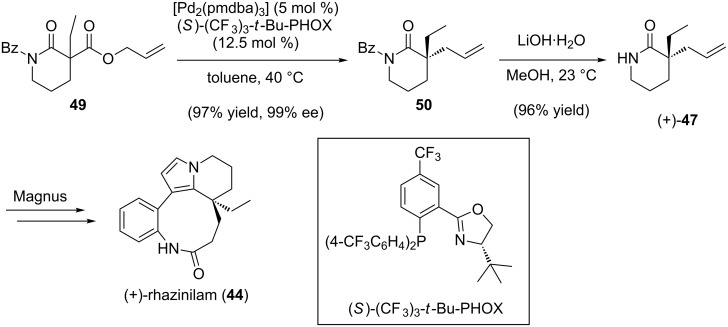
Formal total synthesis of (+)-rhazinilam.

The formal synthesis of (+)-rhazinilam commenced with palladium-catalyzed decarboxylative allylic alkylation of known carboxy-lactam **49** to afford benzoyl-protected piperidinone **50** in 97% yield and 99% ee (Scheme 11) [84]. Cleavage of the benzoyl group under basic conditions furnished piperidinone (+)-**47** [84], which can be advanced to (+)-rhazinilam via Magnus’ route. This formal synthesis demonstrates the utility of our recently developed asymmetric lactam alkylation chemistry.

### F) Quebrachamine

Quebrachamine (**51**) is an indole alkaloid isolated from the *Aspidosperma quebracho* tree bark [60]. It has been found to possess adrenergic blocking activities for a variety of urogenital tissues [85]. Structurally, it features a tetracycle including an indole nucleus, a 9-membered macrocycle, and an all-carbon quaternary stereocenter. Due to its structural complexity and biological activities, quebrachamine has received considerable attention from the chemistry community. A number of total syntheses have been reported [86–88], with several examples of asymmetric syntheses [89–91].

**Scheme 12:**
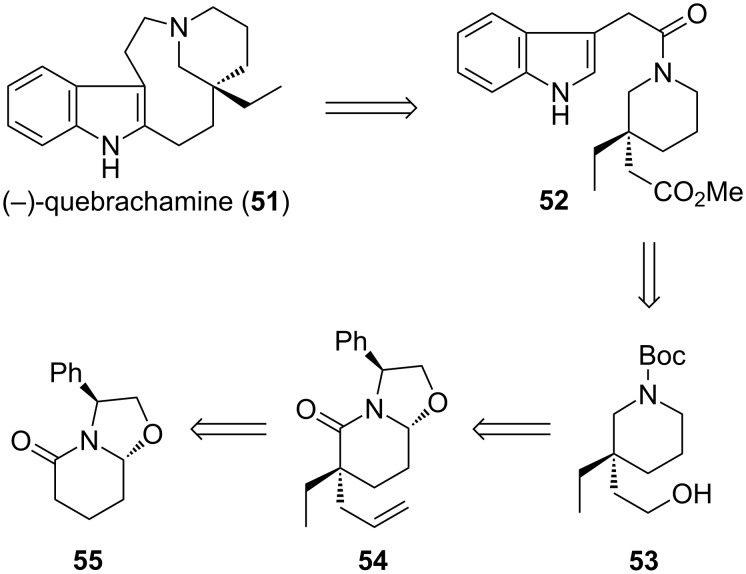
Amat’s approach to (−)-quebrachamine.

In 2007, Amat reported an enantioselective total synthesis of quebrachamine (Scheme 12) [92]. In their planning, disconnection at the macrocycle led to amide **52**, which was prepared from 3,3-disubstituted piperidine **53**. The all-carbon quaternary stereocenter in **53** was installed by double alkylation of lactam **55**, using an auxiliary to control the stereoselectivity. We envisioned that an alternative way of constructing this motif would again make use of our recently developed palladium-catalyzed asymmetric alkylation of lactam enolates.

**Scheme 13:**
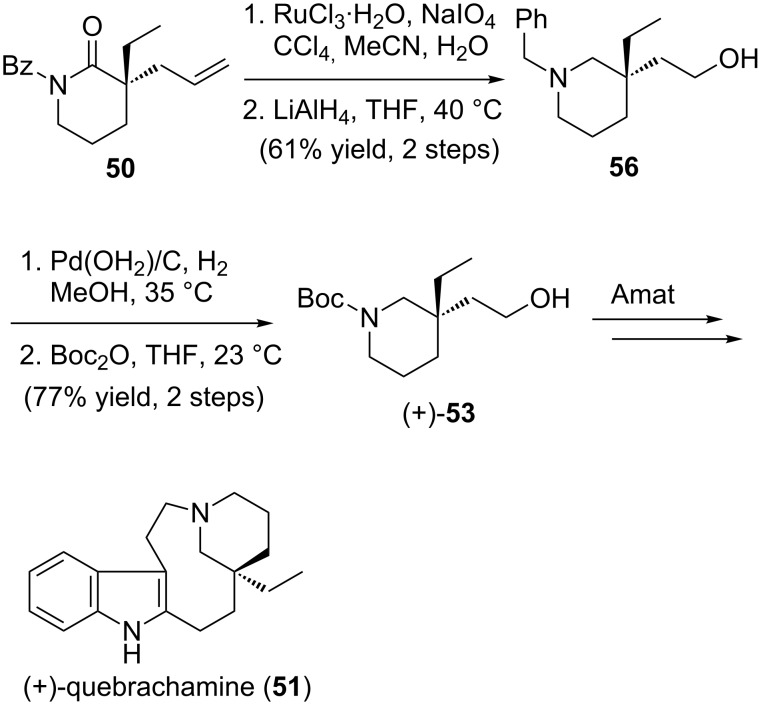
Formal total synthesis of (+)-quebrachamine.

The formal synthesis of (+)-quebrachamine commenced with benzoyl lactam **50** (Scheme 13), which was prepared in excellent yield and ee by alkylation of carboxy-lactam **49** (see Scheme 11) [84]. Oxidative cleavage of the terminal double bond and subsequent reduction with LiAlH_4_ afforded *N*-benzylpiperidine-alcohol **56** [84]. Hydrogenolysis of the *N*-benzyl group and re-protection with di-*tert*-butyl dicarbonate furnished *N*-boc piperidine-alcohol (+)-**53** [84], thus intercepting an intermediate in Amat’s synthesis of quebrachamine.

### G) Vincadifformine

Vincadifformine (**59**) was isolated in both enantioenriched and racemic forms from the leaves and roots of *Rhazya stricta* in 1963 [93]. Not only is it a representative member of the *Aspidosperma* alkaloid family, but it also holds particular significance as a valuable precursor to pharmaceutically important vincamine, vincamone, and cavinton [94–97]. The molecule has a fused pentacyclic framework with three contiguous stereocenters, two of which are all-carbon quaternary centers. The medicinal relevance and structural complexity of vincadifformine have led to a large number of total syntheses [98–104], including several enantioselective examples [105–108].

**Scheme 14:**
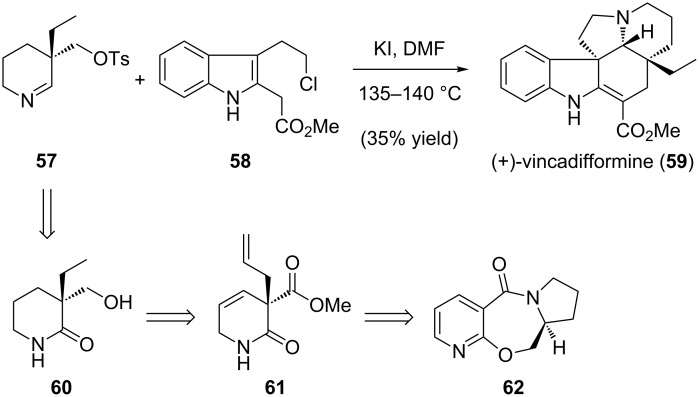
Pandey’s approach to (+)-vincadifformine.

Recently, Pandey reported a highly efficient synthesis of (+)-vincadifformine (Scheme 14) [106]. The key step in the synthesis was an iminium ion cascade reaction that formed the fused ring systems by coupling 3,3-disubstituted tetrahydropyridine **57** with indole derivative **58**. The former coupling partner was derived from chiral α-quaternary lactam **60**, which was constructed using a chiral auxiliary strategy. We envisioned that chiral lactam **60** could again be readily accessed by our palladium-catalyzed enantioselective alkylation chemistry.

**Scheme 15:**
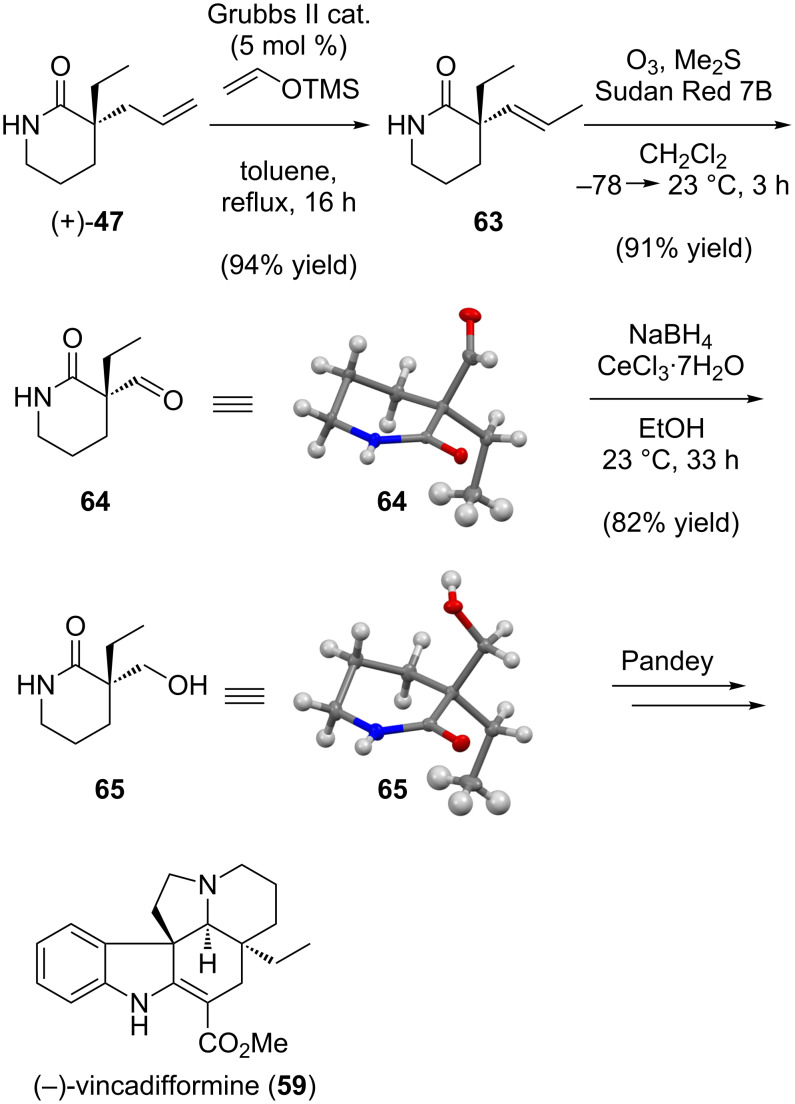
Formal total synthesis of (−)-vincadifformine.

The formal synthesis of (−)-vincadifformine commenced with ruthenium-catalyzed isomerization of the terminal olefin moiety in unprotected piperidinone (+)-**47** (made previously in the formal synthesis of (+)-rhazinilam shown in Scheme 11) to produce internal olefin **63** (Scheme 15) [109]. Ozonolysis of the double bond furnished aldehyde **64**, which was reduced under Luche conditions to alcohol **65**, a compound identical in structure and enantiomeric to the intermediate employed by Pandey in the synthesis of (+)-vincadifformine.

## Conclusion

**Scheme 16:**
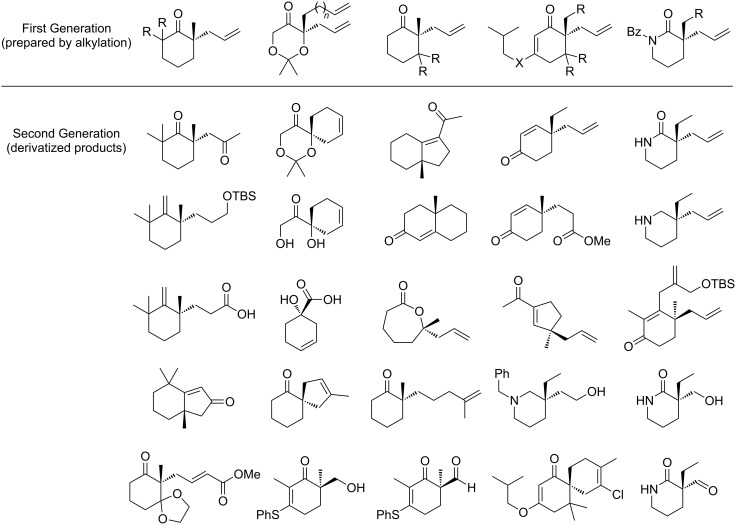
Two generations of building blocks.

The development of a series of Pd-catalyzed methods for constructing stereogenic quaternary carbons has provided two generations of building blocks (Scheme 16). The described derivatization enabled the formal total syntheses of an array of classic natural products including sugar derivatives, terpenes, and alkaloids, adding significantly to the growing list of uses for this powerful C–C bond construction. An efficient route to the sesquiterpenoid (−)-thujopsene (**10**) has been delineated, allowing access to the compound’s natural antipode. Our lab’s novel approach to (−)-quinic acid (**21**) allowed access to either enantiomer of this important substance. We have also intercepted a key intermediate in Danishefsky’s synthesis of (±)-dysidiolide (**29**), rendering the former racemic route enantioselective. Additionally, a rapid approach to a compound in Meyers’ formal synthesis of (+)-aspidospermine (**36**) granted access to the natural product without the use of a chiral auxiliary. Finally, we have demonstrated the application of lactam alkylation products in the catalytic asymmetric syntheses of (+)-rhazinilam (**44**), (+)-quebrachamine (**51**), and (−)-vincadifformine (**59**). The powerful catalytic enantioselective allylic alkylation will undoubtedly enable new synthetic endeavors in the context of both academic and industrial research.

## Supporting Information

Supporting information features experimental procedures, characterization data of synthesized compounds, copies of ^1^H and ^13^C NMR spectra, and single crystal structure data.

File 1 Experimental data, NMR spectra and X-ray data.
