# Supplementary material for: Formal total syntheses of classic natural product target molecules via palladium-catalyzed enantioselective alkylation
Source: Beilstein J Org Chem. 2014 Oct 28;10:2501–12. doi: 10.3762/bjoc.10.261 (PMC4222294; doi:10.3762/bjoc.10.261)
Supplement: File 1 — Experimental data, NMR spectra and X-ray data. [file Beilstein_J_Org_Chem-10-2501-s001.pdf]

Supporting Information

for

Formal total syntheses of classic natural product target molecules via palladium-catalyzed enantioselective alkylation

Yiyang Liu, Marc Liniger, Ryan M. McFadden, Jenny L. Roizen, Jacquie Malette, Corey M. Reeves, Douglas C. Behenna, Masaki Seto, Jimin Kim, Justin T. Mohr, Scott C. Virgil and Brian M. Stoltz*

Address: Warren and Katharine Schlinger Laboratory of Chemistry and Chemical Engineering, Division of Chemistry and Chemical Engineering, California Institute of Technology, 1200 E. California Boulevard, Pasadena, CA, USA

Email: Brian M. Stoltz* - stoltz@caltech.edu

* Corresponding author

Experimental data, NMR spectra and X-ray data

Table of Contents

Experimental Procedures **S2**

Materials and Methods **S2**

Syntheses of Compounds Related to Thujopsene **S3**

Syntheses of Compounds Related to Quinic Acid **S8**

Syntheses of Compounds Related to Dysidiolide **S8**

Syntheses of Compounds Related to Aspidospermine **S11**

Syntheses of Compounds Related to Rhazinilam **S14**

Syntheses of Compounds Related to Quebrachamine **S14**

Syntheses of Compounds Related to Vincadifformine **S15**

Methods for the Determination of Enantiomeric Excess **S19**

^{1}H and ^{13}C NMR Spectra **S21**

X-Ray Structure Determination **S47**

Experimental Procedures

Materials and Methods

Unless stated otherwise, reactions were conducted in flame-dried glassware under an atmosphere of nitrogen using anhydrous solvents (either freshly distilled or passed through activated alumina columns). Chloroform, stabilized with ethanol, was stored in the dark over oven-dried 4 Å molecular sieves. Absolute ethanol, methanol, and *N*,*N*-dimethyl acetamide were used as purchased. 2,2,6-Trimethylcyclohexanone (**16**) was used as received. TMEDA and iPr_2NH were distilled from CaH_2. All other commercially obtained reagents were used as received unless specified otherwise. (*S*)-*t*-Bu-PHOX ligand (***S***)-**5** was prepared according to known methods.[1] Reaction temperatures were controlled using an IKAmag temperature modulator. Thin-layer chromatography (TLC) was conducted with E. Merck silica gel 60 F254 pre-coated plates (0.25 mm) and visualized using UV at 254 nm, *p*-anisaldehyde, potassium permanganate, and iodine vapor over sand. TLC data include R_f, eluent, and method of visualization. ICN silica gel (particle size 0.032–0.063 mm), SilliaFlash P60 Academic silica gel (0.040–0.063 mm), or Florisil (Aldrich) was used for flash column chromatography. Analytical chiral HPLC analyses were performed with an Agilent 1100 Series HPLC using a chiralcel OD or AD normal-phase column (250 × 4.6 mm) employing 2.0–3.0% ethanol in hexane isocratic elution and a flow rate of 0.1 mL/min with visualization at 254 nm. Analytical chiral GC analysis was performed with an Agilent 6850 GC using a GT-A column (0.25 m × 30.00 m) employing an 80 °C isotherm and a flow rate of 1.0 mL/min. ^{1}H NMR spectra were recorded on a Varian Mercury 300 (at 300 MHz) or a Varian Inova 500 (at 500 MHz) and are reported relative to the residual solvent peak (δ 7.26 for $CDCl_3$ and δ 7.16 for C_6D_6). Data for ^{1}H NMR spectra are reported as follows: chemical shift (δ ppm), multiplicity, coupling constant (Hz),[2] and

integration. ^{13}C NMR spectra were recorded on a Varian Mercury 300 (at 75 MHz) or a Varian Inova 500 (at 125 MHz) and are reported relative the residual solvent peak (δ 77.2 for $CDCl_3$ and δ 128.4 for C_6D_6). Data for ^{13}C NMR spectra are reported in terms of chemical shift, and integration (where appropriate). IR spectra were recorded on a Perkin Elmer Spectrum BXII spectrometer and are reported in frequency of absorption (cm^{-1}). IR samples were thin films deposited on sodium chloride plates by evaporation from a solvent (usually $CDCl_3$), which is recorded. Optical rotations were measured with a Jasco P-1010 polarimeter, using a 100 mm path-length cell. High-resolution mass spectra were obtained from the California Institute of Technology Mass Spectral Facility. Melting points were determined on a Thomas-Hoover melting point apparatus and are uncorrected.

Syntheses of Compounds Related to Thujopsene

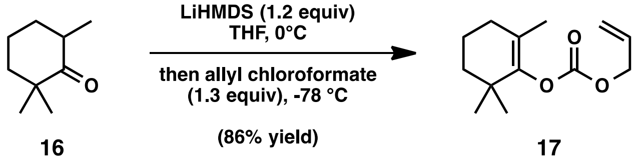

16 17

Enol Carbonate 17. Preparation and characterization of this compound was reported in our previous publication.[3]

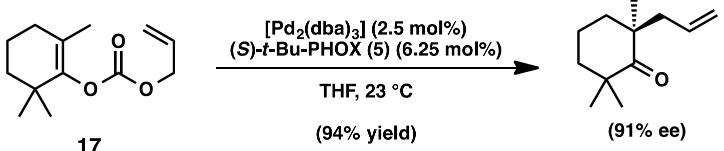

17 (91% ee) (–)-18

Allyl Ketone (–)-18. A round bottom flask was flame-dried under argon and cycled into the glovebox. It was charged with [$Pd_2(dba)_3$] (242 mg, 0.264 mmol, 6.25 mol%) and (*S*)-*t*-Bu-PHOX (***(S)*-5**)(256 mg, 0.661 mmol, 2.5 mol%). Then, THF (317 mL) was introduced. The red mixture was stirred for 20 min at 25 °C. Then, enol carbonate **17** (2.37 g, 10.57 mmol, 1.00 equiv) in THF (10 mL) was added. After the reaction was gauged complete using TLC analysis, it was removed from the glovebox, then concentrated. PhH (~20 mL) was added. After concentrating a second time, more PhH (~20 mL) was added. The solution was purified by flash chromatography on silica gel (2:98 Et_2O/hexane eluent), affording allyl ketone **(–)-18** (1.72 g, 94% yield) as a clear oil in 91% ee as determined by chiral HPLC analysis. R_f 0.48 (1:9 EtOAc/hexane), (I_2/sand, brown spot); ^{1}H NMR (300 MHz, $CDCl_3$): δ 5.64 (dddd, *J* = 17.1 Hz, 10.5 Hz, 7.7 Hz, 6.9 Hz, 1H), 5.05 (app. ddt, J_{d1} = 6.3 Hz, J_{d2} = 2.2 Hz, J_t = 1.1 Hz, 1H), 4.98 (app. ddt, J_{d1} = 13.8 Hz, J_{d2} = 2.5 Hz, J_t = 1.4 Hz, 1H), 2.32 (app. ddt, J_{d1} = 13.8 Hz, J_{d2} = 6.9 Hz, J_t = 1.4 Hz, 1H), 2.16 (app. ddt, J_{d1} = 13.8 Hz, J_{d2} = 6.9 Hz, J_t = 1.4 Hz, 1H) 1.87–1.47 (m, 6H), 1.15 (s, 3H), 1.09 (s, 3H), 1.08 (s, 3H); ^{13}C NMR (75 MHz, $CDCl_3$): δ 219.8, 134.7, 118.0, 47.7, 44.6, 44.0, 39.9, 37.0, 28.0, 27.3, 25.7, 17.9; IR (NaCl/$CDCl_3$): 3077, 2979, 2964, 2933, 2869, 1697, 1463, 1382, 999, 914 cm^{-1}; HRMS-EI$^+$ (*m/z*): [M]$^+$ calc'd for $C_{12}H_{20}O$, 180.1514; found, 180.1506; $[\alpha]_D^{24}$ –36.3° (*c* 0.140, $CHCl_3$), 91% ee. For literature data see [3,5].

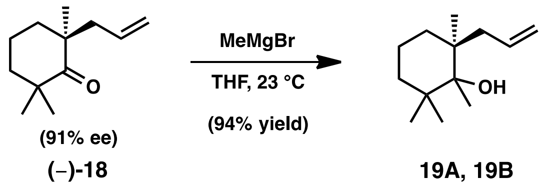

Alcohols 19A and 19B. A round-bottom flask was charged with a solution of allyl ketone **(–)-18** (1.02 g, 5.65 mmol, 1.00 equiv, 91% ee) and THF (55.5 mL). Then, methyl magnesium bromide

(3.0 M in Et_2O, 9.25 mL, 27.8 mmol, 5.00 equiv) was gradually introduced at 23 °C. After 24 h, the reaction was carefully quenched at 0 °C with sat. aq NH_4Cl (30 mL). Then H_2O (50 mL) was added, along with hexanes (50 mL). The biphasic mixture was extracted with Et_2O (2 x 30 mL). All organic layers were combined, dried (Na_2SO_4), filtered, and concentrated. The wet residue was taken up in $CHCl_3$ and dried again with Na_2SO_4, then filtered. The filtrate was concentrated, giving a 1:1 mixture of diastereomeric alcohols **19A** and **19B** (1.04 g, 94% yield) as a colorless oil. R_f 0.59 (10:90 EtOAc/hexane), (*p*-Anisaldehyde, violet spot); ^{1}H NMR (300 MHz, $CDCl_3$) (both diastereomers): δ 5.84 (app. dddd, *J* = 19.4 Hz, 14.6 Hz, 7.4 Hz, 7.2 Hz, 2H), 5.01 (app. d, *J* = 11.1 Hz, 2H), 5.00 (app. d, *J* = 14.6 Hz, 2H), 2.44 (app. ddd, *J* = 12.6 Hz, 11.1 Hz, 7.5 Hz, 2H), 2.07 (app. ddd, *J* = 19.4 Hz, 13.6 Hz, 7.7 Hz, 2H), 1.62–1.46 (m, 4H), 1.44–1.36 (m, 4H), 1.28–1.10 (m, 2H), 1.14 (app. s, 6H), 1.07 (s, 3H), 1.06 (s, 3H), 1.10 (s, 3H), 0.99 (s, 3H), 0.98–0.86 (m, 2H), 0.97 (s, 3H), 0.95 (s, 3H); ^{13}C NMR (75 MHz, $CDCl_3$) (both diastereomers): δ 136.8, 136.4, 117.0, 116.8, 78.2, 77.9, 43.8, 42.0, 41.6, 41.2, 39.2, 39.0, 37.2, 36.9, 33.6, 33.0, 28.3, 28.2, 26.6, 25.8, 22.9, 22.2, 18.6, 18.5, 18.3, 18.1; IR (NaCl/$CDCl_3$): 3504 (broad), 3074, 2930, 2867, 1638, 1454, 1378, 1305, 1071, 998, 910 cm^{-1}; HRMS-EI$^+$ (*m/z*): $[M]^+$ calc'd for $C_{13}H_{24}O$, 196.1827; found, 196.1803.

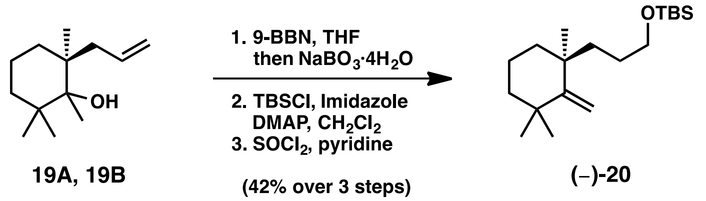

19A, 19B

(–)-20

Methylene cyclohexane (–)-20. A 20 mL scintillation vial containing a mixture of diastereomeric alcohols **19A** and **19B** (72 mg, 0.367 mmol, 1.00 equiv, 91% ee) was treated with a solution of 9-borabicyclo[3.3.1]nonane in THF (0.5 M, 0.90 mL, 0.45 mmol, 1.23 equiv) at 23

°C. The reaction was stirred for 2.5 h. Then the reaction was cooled to 0 °C, and H_2O (1 mL) was carefully added, followed by $NaBO_3•4H_2O$ (219 g, 1.42 mmol, 3.88 equiv). The biphasic reaction mixture was stirred vigorously at 23 °C for 2 h, diluted with water, and extracted with CH_2Cl_2 (4 x 1 mL). All organic layers were combined, dried (Na_2SO_4), filtered, and concentrated. The residue was purified by flash chromatography on silica gel (25:75 EtOAc:hexane → 33:67 EtOAc:hexane → 50:50 EtOAc:hexane eluent), giving an oil containing two diastereomeric products, which was immediately used in the next reaction.

This mixture was transferred to a 20 mL scintillation vial. Imidazole (39 mg, 0.57 mmol), 4-dimethylaminopyridine (1 mg, 0.00885 mmol), and anhydrous CH_2Cl_2 (1.0 mL) were introduced, followed by a solution of TBSCl (48 mg, 0.314 mmol) in anhydrous CH_2Cl_2 (1.0 mL) at 23 °C. A white precipitate quickly formed. After 10 min, the reaction was diluted with hexanes (4 mL), filtered, and concentrated. The residue was purified by flash chromatography on silica gel (5:95 EtOAc:hexane eluent), affording a diastereomeric mixture of silyl ethers. This composite was carried on to the next reaction without further characterization.

The mixture of silyl ethers was transferred to a 20 mL scintillation vial, which was charged with pyridine (freshly distilled from CaH_2, 1.5 mL). After cooling to 0 °C, $SOCl_2$ (36 μL, 0.50 mmol) was slowly introduced. After stirring 1 h at 0 °C and another 1 h at 23 °C, H_2O (5 mL) was carefully added, followed by Et_2O (8 mL). The organic phase was collected, and the aqueous layer was extracted with Et_2O (2 x 10 mL). All organic layers were combined and washed with 1.0 M aq $CuSO_4$ (4 x 5 mL). The aqueous washings were back-extracted with Et_2O (1 x 10 mL). All organic layers were combined, dried (Na_2SO_4), filtered, and concentrated. The residue was purified by flash chromatography on silica gel (1:99 Et_2O:hexane → 2:98 Et_2O:hexane eluent), giving pure methylene cyclohexane **(–)-20** (48 mg, 42% yield from **19A**

and **19B**) as a colorless oil. R_f 0.71 (10:90 EtOAc/hexane), (*p*-Anisaldehyde, blue spot); ^{1}H NMR (300 MHz, $CDCl_3$): δ 5.00 (app. s, 1H), 4.79 (app. s, 1H), 3.57 (app. t, J = 6.6 Hz, 2H), 1.80–1.64 (m, 2H), 1.62–1.16 (m, 8H), 1.11 (s, 3H), 1.10 (s, 3H), 1.04 (s, 3H), 0.89 (s, 9H), 0.04 (s, 6H); ^{13}C NMR (75 MHz, $CDCl_3$): δ 160.5, 108.7, 64.1, 41.8, 40.8, 39.4, 36.6, 36.5, 32.8, 29.9, 29.8, 28.4, 26.2 (3C), 18.7, 18.6, 5.0 (2C); IR (NaCl/$CDCl_3$): 3100, 2955, 2929, 2858, 1623, 1472, 1382, 1361, 1255, 1100, 940, 900, 836, 774 cm^{-1}; HRMS-EI$^+$ (*m/z*): $[M]^+$ calc'd for $C_{19}H_{38}SiO$, 310.2692; found, 310.2689. $[\alpha]_D^{24}$ –18.8° (*c* 1.90, $CHCl_3$), 91% ee.

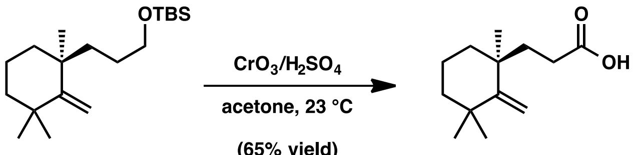

(–)-20 (–)-12

Carboxylic Acid (–)-12. A vessel containing methylene cyclohexane **(–)-20** (48 mg, 0.154 mmol) was charged with acetone (ACS grade, 2.5 mL), then treated with Jones reagent (1.0 M CrO_3, 4.0 H_2SO_4 in H_2O)(1.0 mL, dropwise from a glass pipet) at 23 °C. After 15 min, the reaction was carefully quenched with sat. aq Na_2SO_3 (2 mL). $CHCl_3$ (5 mL) was added, followed by H_2O (5 mL) and 6 M aq HCl (4 mL). After 5 min, the reaction was extracted with $CHCl_3$ (3 x 10 mL). All organic layers were combined, dried (Na_2SO_4), filtered, and concentrated. The residue was purified by flash chromatography on silica gel (6:94 Et_2O:CH_2Cl_2 → 14:86 Et_2O:CH_2Cl_2 eluent), giving carboxylic acid (–)-**12** (21 mg, 65% yield) as a colorless oil. R_f 0.17 (10:90 EtOAc/hexane), (*p*-Anisaldehyde, blue spot); ^{1}H NMR (300 MHz, $CDCl_3$): δ 5.06 (app. s, 1H), 4.80 (app. s, 1H), 2.36–2.04 (m, 3H), 1.82–1.66 (m, 2H), 1.60–1.30 (m, 5H), 1.11 (s, 3H), 1.10 (s, 3H), 1.05 (s, 3H); ^{13}C NMR (75 MHz, $CDCl_3$): δ 159.3, 109.6, 41.5, 40.6, 39.2, 36.5, 34.7, 32.7, 29.61, 29.56, 18.6; IR (NaCl/$CDCl_3$): 3000 (broad), 2927, 1708, 1462, 1414, 1380,

1296, 1095, 902 cm^{-1}; HRMS-EI$^+$ (*m/z*): [M]$^+$ calc'd for $C_{13}H_{22}O$, 210.1620; found, 210.1618. $[\alpha]_D^{24}$ –27.8° (*c* 1.205, $CHCl_3$), 91% ee.

Syntheses of Compounds Related to Quinic Acid

Preparation and characterization of these compounds was reported in our previous publication.[4]

Syntheses of Compounds Related to Dysidiolide

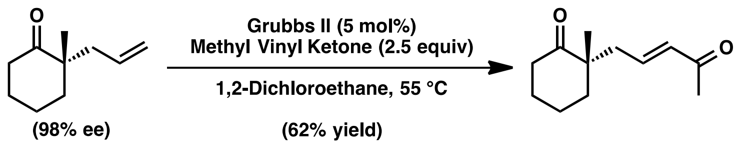

Keto-Enone 34.[5] A vial was charged with allyl ketone **2** (45.2 mg, 0.297 mmol, 1.0 equiv, 98% ee), followed by a solution of methyl vinyl ketone (61.8 μL, 0.743 mmol, 2.5 equiv) in 1,2-dichloroethane (1.5 mL). Then, Grubbs 2nd generation catalyst (12.6 mg, 14.9 μmol, 5 mol%) was added. The vessel was sealed and warmed to 55 °C for 24 h. The reaction transitioned from maroon to deep green. The reaction was cooled to 23 °C and concentrated. The residue was purified by flash chromatography on silica gel (hexane → 20:80 EtOAc:hexane eluent), giving keto-enone **34** (35.7 mg, 62% yield) as a pale brown oil. R_f 0.23 (20:80 EtOAc/hexane), (UV, 254 nm); ^{1}H NMR (300 MHz, $CDCl_3$): δ 6.70 (app. dt, J_d = 15.9 Hz, J_t = 7.4 Hz, 1H), 6.03 (app. d, J = 15.9 Hz, 1H), 2.50–2.26 (m, 2H), 2.40 (app. d, J = 6.9 Hz, 1H), 2.39 (app. d, J = 6.9 Hz, 1H), 2.22 (s, 3H), 1.91–1.81 (m, 2H), 1.80–1.60 (m, 4H), 1.12 (s, 3H); ^{13}C NMR (75 MHz, $CDCl_3$): δ 214.6, 198.4, 144.1, 134.2, 48.7, 41.0, 38.9, 38.7, 27.4, 26.9, 23.1, 21.1; IR

(NaCl/$CDCl_3$): 2935, 2866, 1704, 1672, 1626, 1426, 1361, 1254, 1124, 986 cm^{-1}; HRMS-EI$^+$ (*m/z*): $[M]^+$ calc'd for $C_{12}H_{18}O_2$, 194.1307; found, 194.1336. $[\alpha]_D^{22}$ –1.14° (*c* 1.415, $CHCl_3$), 98% ee.

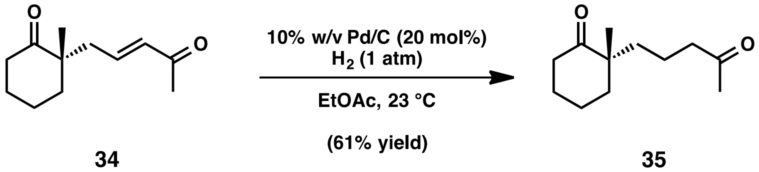

34 35

Diketone 35.[5] A round-bottom flask containing keto-enone **34** (28.0 mg, 0.144 mmol, 1.0 equiv) in EtOAc (3.0 mL) was sparged with argon for 2 min. Pd/C (10% w/w) (30.6 mg, 28.8 μmol, 20 mol) was introduced, and the reaction was cooled to –78 °C. It was purged/backfilled with vacuum/H_2 (1 atm) (3 x) and warmed to 23 °C and stirred under H_2 (1 atm) for 12 h. More EtOAc (5 mL) was added, and the reaction was sparged with argon to remove residual H_2. The material was filtered through a plug of silica gel with the aide of EtOAc. The filtrate was concentrated, affording diketone **35** (17.3 mg, 61% yield) as a pale yellow oil. R_f 0.26 (20:80 EtOAc/hexane), (*p*-Anisaldehyde, peach spot); ^{1}H NMR (300 MHz, $CDCl_3$): δ 2.40 (app. t, *J* = 6.6 Hz, 2H), 2.36 (app. t, *J* = 5.5 Hz, 2H), 2.11 (s, 3H), 1.90–1.44 (m, 9H), 1.36 (app. d, *J* = 7.7 Hz, 1H), 1.15 (s, 3H); ^{13}C NMR (75 MHz, $CDCl_3$): δ 216.0, 208.8, 48.6, 44.0, 39.2, 38.9, 37.0, 30.1, 27.6, 22.7, 21.2, 18.2; IR (NaCl/$CDCl_3$): 2936, 2865, 1705, 1452, 1360, 1167, 1123, 1099 cm^{-1}; HRMS-EI$^+$ (*m/z*): $[M]^+$ calc'd for $C_{12}H_{20}O_2$, 196.1463; found, 196.1469. $[\alpha]_D^{22}$ –42.3° (*c* 0.865, $CHCl_3$), 98% ee.

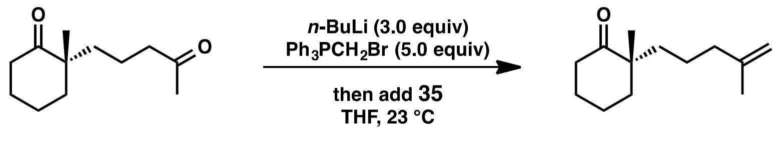

35 32

Keto-Olefin 32. A round-bottom flask was charged with methyl triphenyl phosphonium bromide (weighed in glovebox, 260 mg, 0.688 mmol, 5.0 equiv). THF (5.5 mL) was introduced, followed by *n*-BuLi (2.5 M in hexane, 165 μL, 0.413 mmol, 3.0 equiv) at 23 °C. After stirring for 1 h, a solution of diketone **35** (27.0 mg, 0.138 mmol, 1.0 equiv) in THF (2.0 mL) was added. 30 min later, the reaction was quenched with sat. aq NH_4Cl (4.0 mL). Then, the reaction was diluted with H_2O (20 mL) and hexane (15 mL). The biphasic mixture was extracted with EtOAc (4 x 20 mL). All organic layers were combined, dried (Na_2SO_4), filtered, and concentrated. The residue was purified by flash chromatography on silica gel (hexane → 2:98 EtOAc:hexane eluent), giving keto-olefin **32** (17.3 mg, 65% yield) as a colorless oil. R_f 0.75 (20:80 EtOAc/hexane), (*p*-Anisaldehyde, blue spot); ^{1}H NMR (300 MHz, $CDCl_3$): δ 4.70 (app. s, 1H), 4.65 (app. s, 1H), 2.46–2.26 (m, 2H), 1.98 (app. t, *J* = 7.1 Hz, 2H), 1.94–1.84 (m, 1H), 1.82–1.50 (m, 5H), 1.68 (s, 3H), 1.47–1.39 (m, 1H), 1.38 (app. ddd, *J* = 26.4 Hz, 12.6 Hz, 4.1 Hz, 1H), 1.22–1.10 (m, 2H), 1.14 (s, 3H); ^{13}C NMR (75 MHz, $CDCl_3$): δ 216.3, 145.7, 110.3, 48.7, 39.6, 39.0, 38.4, 37.2, 27.7, 22.8, 22.5, 21.7, 21.2; IR (NaCl/$CDCl_3$): 3074, 2936, 2865, 1707, 1650, 1452, 1376, 1260, 1096, 1020, 886, 804 cm^{-1}; HRMS-EI$^+$ (*m/z*): $[M]^+$ calc'd for $C_{13}H_{22}O$, 194.1671; found, 194.1680. $[\alpha]_D^{21}$ –49.8° (*c* 0.865, $CHCl_3$), 98% ee. Literature optical rotation: $[\alpha]_D^{25}$ –55.2° (*c* 0.25, $CHCl_3$).[6]

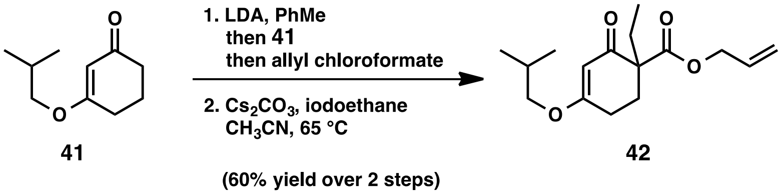

α–Ethyl–α–Allyloxycarbonyl Vinylogous Ester 42. A round-bottom flask was flamedried under argon and charged with dry PhMe (320 mL). Then, iPr_2NH (12.81 mL, 91.3 mmol, 2.05 equiv) was introduced. The reaction was cooled to –78 °C, and *n*-BuLi (2.5 M in hexane, 35.68 mL, 89.2 mmol, 2.00 equiv) was added slowly. The reaction was warmed to 0 °C for 15 min, then promptly cooled back to –78 °C. Then, a solution of vinylogous acid **41** (7.50 g, 44.6 mmol, 1.00 equiv) in PhMe (20 mL) was added at –78 °C over a 5 min period. After 40 min had passed, the reaction was treated with allyl chloroformate (4.97 mL, 46.8 mmol, 1.05 equiv) over a 5 min timeframe at –78 °C. After 15 min, the reaction was warmed to 23 °C and stirred for 1 h, during which the reaction went from yellow to orange. Then, 1.0 M aq $KHSO_4$ (127 mL) was added with vigorous stirring, causing the reaction to turn yellow. The organic phase was collected. The aqueous layer was extracted with Et_2O (2 x 50 mL). All organic layers were combined, dried (Na_2SO_4), filtered, and concentrated, giving a crude α–allyloxycarbonyl vinylogous ester as an orange oil, which was immediately used in the next reaction.

A round-bottom flask containing the crude vinylogous ester was charged with CH_3CN (45 mL), followed by iodoethane (14.26 mL, 178.4 mmol, 4.0 equiv relative to **41**). Anhydrous Cs_2CO_3 (29.06 g, 89.2 mmol, 2.0 equiv relative to **41**) was introduced, and the reaction was stirred vigorously at 65 °C for 12 h. The reaction was cooled to 23 °C and filtered over glass frits. The filtrate was concentrated in vacuo, and the residue was purified by flash column chromatography on silica gel (hexane → 15:85 EtOAc:hexane eluent), giving semipure **42**. The

product-containing fractions were combined and concentrated, and the resulting residue was purified on a second silica gel flash column (5:95 EtOAc:CH_2Cl_2 eluent), giving pure α–ethyl–α–allyloxycarbonyl vinylogous ester **42** (7.47 g, 60% yield over 2 steps) as a yellow oil. R_f 0.44 (20:80 EtOAc/hexane), (*p*-Anisaldehyde, brownish-blue spot); ^{1}H NMR (300 MHz, $CDCl_3$): δ 5.83 (ddt, J_{d1} = 16.2 Hz, J_{d2} = 10.7 Hz, J_t = 5.7 Hz, 1H), 5.31 (s, 1H), 5.24 (app. ddd, *J* = 16.2 Hz, 2.9 Hz, 1.5 Hz, 1H), 5.15 (app. ddd, *J* = 10.7 Hz, 2.9 Hz, 1.5 Hz, 1H), 4.56 (app. dt, J_d = 5.4 Hz, J_t = 1.5 Hz, 2H), 3.54 (d, *J* = 6.7 Hz, 2H), 2.68–2.28 (m, 2H), 2.42–2.26 (m, 1H), 1.99 (dq, J_d = 22.2 Hz J_q = 7.4 Hz, 1H), 1.97–1.85 (m, 2H), 1.78 (dq, J_d = 22.2 Hz, J_q = 7.4 Hz, 1H), 0.92 (d, *J* = 6.9 Hz, 6H), 0.86 (t, *J* = 7.4 Hz, 3H); ^{13}C NMR (75 MHz, $CDCl_3$): δ 195.8, 176.8, 171.6, 131.9, 118.2, 102.2, 74.9, 65.5, 56.3, 27.77, 27.76, 27.0, 26.4, 19.1, 9.1; IR (NaCl/$CDCl_3$): 3083, 2963, 2939, 2879, 1731, 1664, 1610, 1470, 1384, 1236, 1195, 1178, 1119, 998, 919 cm^{-1}; HRMS-EI$^+$ (*m/z*): [M]$^+$ calc'd for $C_{16}H_{24}O_4$, 280.1687; found, 280.1687.

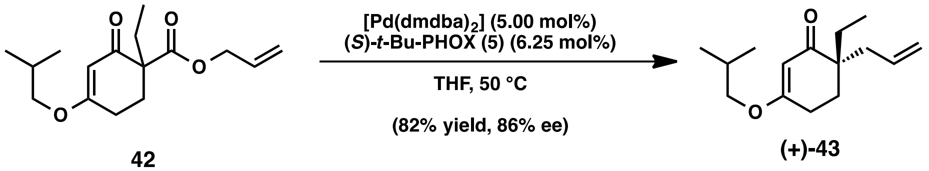

42

(+)-43

Allyl Vinylogous Ester (+)-43 (86% ee). In the glovebox, a flame-dried round-bottom flask was charged with [Pd(dmdba)$_2$] (40.8 mg, 50.0 μmol, 5.00 mol%) and (*S*)-*t*-butyl phosphinooxazoline (24.2 mg, 62.5 μmol, 6.25 mol%) and removed from the glovebox. THF (30 mL) was added, and the reaction stirred at 23 °C for 30 min. Then, a solution of α–ethyl–β–allyloxycarbonyl vinylogous ester **42** (280 mg, 1.00 mmol, 1.00 equiv) in THF (3.0 mL) was added. The reactor was quickly fitted with a reflux condenser, and the reaction was heated to 50 °C under N_2 for 24 h. During this time the reaction went from orange to green. The

reaction was cooled to 23 °C and concentrated. The residue was purified by flash chromatography on silica gel (hexane → 5:95 EtOAc:hexane eluent), giving allyl vinylogous ester **(+)-43** (193.4 mg, 82% yield) in 86% ee (as determined by chiral HPLC assay) as a yellow oil. R_f 0.58 (20:80 EtOAc/hexane), (UV, 254 nm); ^{1}H NMR (300 MHz, $CDCl_3$): δ 5.73 (app. dddd, *J* = 17.0 Hz, 10.5 Hz, 7.7 Hz, 6.9 Hz, 1H), 5.24 (s, 1H), 5.08–5.04 (m, 1H), 5.04–5.00 (m, 1H), 3.57 (d, *J* = 6.6 Hz, 2H), 2.42 (app. td, J_t = 6.6 Hz, J_d = 2.5 Hz, 2H), 2.38 (app. dd, *J* = 14.0 Hz, 7.1 Hz, 1H), 2.19 (app. dd, *J* = 14.0 Hz, 7.1 Hz, 1H), 1.85 (app. t, *J* = 6.6 Hz, 2H), 2.01 (app. septuplet, *J* = 6.6 Hz, 1H), 1.61 (dq, J_d = 22.2 Hz, J_q = 7.4 Hz, 1H), 1.55 (dq, J_d = 22.2 Hz, J_q = 7.4 Hz, 1H), 0.97 (d, *J* = 6.6 Hz, 6H), 0.84 (t, *J* = 7.4 Hz, 3H); ^{13}C NMR (75 MHz, $CDCl_3$): δ 203.0, 176.0, 134.8, 117.8, 102.0, 74.8, 46.6, 39.4, 29.0, 27.9, 27.6, 25.8, 19.2, 8.5; IR (NaCl/$CDCl_3$): 3074, 2963, 2936, 2878, 1652, 1612, 1384, 1193, 1178, 1003 cm^{-1}; HRMS-EI$^+$ (*m/z*): $[M]^+$ calc'd for $C_{15}H_{24}O_2$, 236.1776; found, 236.1788. $[\alpha]_D^{24}$ +10.4° (*c* 0.675, $CHCl_3$), 86% ee.

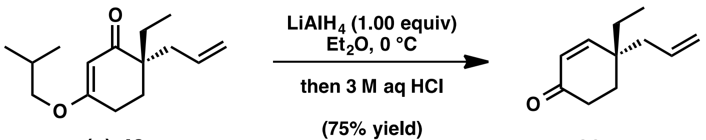

(+)-43 38

γ-Ethyl-γ-Allyl Enone 38. A round-bottom flask was charged with allyl vinylogous ester **(+)-43** (50.0 mg, 0.212 mmol, 95% ee, 1.00 equiv), and the reactor was purged with vacuum/argon (1 x). Et_2O (10.0 mL) was introduced, and the reaction was cooled to 0 °C. $LiAlH_4$ (8.0 mg, 0.212 mmol, 1.00 equiv) was then added, and the reaction was stirred for 1 h. The 3 M aq HCl (10.0 mL) was very cautiously added at 0 °C. Once the addition was complete, the reaction was warmed to 23 °C and stirred vigorously for 5 h. The reaction was transferred to a separatory

funnel and extracted with Et_2O (3 x 10 mL). All organic layers were combined, dried (Na_2SO_4), filtered, and concentrated. The residue, which contained some H_2O, was dissolved in $CHCl_3$ and dried with Na_2SO_4. The mixture was filtered, and the filtrate was concentrated, affording γ-ethyl-γ-allyl enone **(+)-38** (26.2 mg, 75% yield) as a colorless, volatile oil. R_f 0.57 (20:80 EtOAc/hexane), (UV, 254 nm) ^{1}H NMR (300 MHz, $CDCl_3$): δ 6.69 (d, *J* = 10.4 Hz, 1H), 5.91 (d, *J* = 10.4 Hz, 1H), 5.74 (app. ddt, J_{d1} = 16.7 Hz, J_{d2} = 9.9 Hz, J_t = 7.4 Hz, 1H), 5.10 (app. d, *J* = 9.9 Hz, 1H), 5.08 (app. d, *J* = 16.7 Hz, 1H), 2.42 (app. t, *J* = 6.9 Hz, 2H), 2.21 (app. d, *J* = 7.4 Hz, 2H), 1.86 (app. t, *J* = 6.9 Hz, 2H), 1.53 (dq, J_d = 22.2 Hz, J_q = 7.4 Hz, 1H), 1.47 (dq, J_d = 22.2 Hz, J_q = 7.4 Hz, 1H), 0.90 (t, *J* = 7.4 Hz, 3H); ^{13}C NMR (75 MHz, $CDCl_3$): δ 199.9, 158.3, 133.7, 128.4, 118.7, 41.9, 38.7, 34.0, 30.6, 30.4, 8.5; IR (NaCl/$CDCl_3$): 3077, 2966, 2929, 2880, 1682, 1452, 1387, 916, 800 cm^{-1}; HRMS-EI$^+$ (*m/z*): $[M]^+$ calc'd for $C_{11}H_{16}O$, 164.1201; found, 164.1207. $[\alpha]_D^{25}$ +27.5° (*c* 0.524, $CHCl_3$), 95% ee.

Syntheses of Compounds Related to Rhazinilam

Preparation and characterization of these compounds was reported in our previous publication.[7]

Syntheses of Compounds Related to Quebrachamine

Preparation and characterization of these compounds was reported in our previous publication.[7]

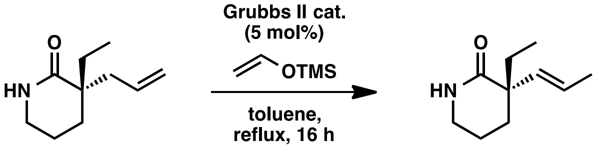

(+)-47 **63**

Disubstituted Alkene 63.[8] To a solution of **(+)-47** (108 mg, 0.65 mmol, 1.0 equiv) and vinyloxytrimethylsilane (0.96 mL, 6.46 mmol, 10 equiv) in toluene (34 mL) was added at rt Grubbs 2nd generation catalyst (27.4 mg, 5 mol%). The purple reaction mixture was immersed in an oil bath (125 °C) (color changed to yellow) and refluxed for 16 h. The reaction mixture was then concentrated under reduced pressure and the residue was purified by column chromatography (hexane/EtOAc, 1:1 → 0:1) to afford **63** (102 mg, 93% conv., 94%) as a brown oil. R_f = 0.20 (hexane/EtOAc, 1:1); ^{1}H NMR (500 MHz, $CDCl_3$): δ 5.94 (brs, 1H), 5.55–5.42 (m, 2H), 3.31–3.22 (m, 2H), 1.91–1.66 (m, 5H), 1.69 (d, *J* = 5.4 Hz, 3H), 1.64–1.54 (m, 1H), 0.84 (t, *J* = 7.4 Hz, 3H); ^{13}C NMR (126 MHz, $CDCl_3$): δ 175.7, 134.8, 124.7, 48.1, 42.7, 31.5, 29.3, 19.1, 18.1, 8.4; IR (NaCl): 3203, 3074, 2936, 2876, 1654, 1489, 1447, 1354, 1298, 1209, 979, 852 cm^{-1}; HRMS (MM: ESI-APCI+) m/z calc'd for $C_{10}H_{17}NO$ $[M+H]^+$: 168.1383, found 168.1385. $[\alpha]_D^{24}$ +10.2° (*c* 1.270, $CHCl_3$).

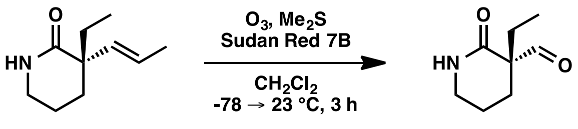

63 **64**

Aldehyde 64. Ozone was bubbled through a cooled (–78 °C) solution of **63** (100 mg, 0.60 mmol, 1.0 equiv) in CH_2Cl_2 (6.0 mL with one drop of sat. Sudan Red 7B CH_2Cl_2 solution) until the reaction mixture turned from bright purple to colorless. Then, the ozone generator was turned off and oxygen was bubbled through for a few minutes. Then, the argon flow was turned on and

dimethylsulfide (0.88 mL, 12.0 mmol, 20 equiv) was added dropwise at –78 °C. After stirring for 30 min at that temperature, the reaction mixture was allowed to warm to rt over 2.5 h. The reaction mixture was then concentrated under reduced pressure and the residue was purified by column chromatography (hexane/EtOAc, 1:2 → EtOAc) to afford **64** (84.7 mg, 91%) as beige crystalline solid. X-ray quality crystals sublimed under high vacuum at rt. R_f = 0.36 (EtOAc); ^{1}H NMR (500 MHz, $CDCl_3$): δ 9.63 (s, 1H), 6.54 (brs, 1H), 3.34–3.20 (m, 2H), 2.33–2.20 (m, 1H), 2.05–1.93 (m, 1H), 1.89–1.75 (m, 2H), 1.73–1.62 (m, 1H), 1.62–1.52 (m, 1H), 0.87 (t, J = 7.5 Hz, 3H); ^{13}C NMR (126 MHz, $CDCl_3$): δ 201.2, 171.1, 58.9, 42.5, 27.3, 24.1, 20.2, 8.2; IR (NaCl): 3290, 2941, 2877, 1727, 1660, 1488, 1462, 1450, 1353, 1323 cm^{-1}; HRMS (MM: ESI-APCI+) m/z calc'd for $C_8H_{13}NO_2$ [M+H]$^+$: 156.1019, found 156.1021; $[\alpha]_D^{24}$ –54.6° (c 1.305, $CHCl_3$); mp: 63–65 °C. X-ray structure has been deposited in the Cambridge Database (CCDC) under the deposition number 1000826.

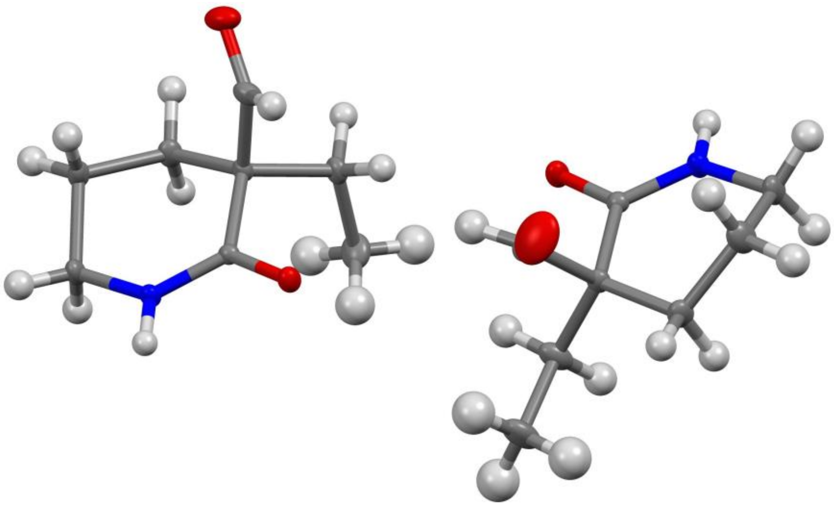

Figure 1: Crystal structure of **64** (ellipsoids, 50% probability level).

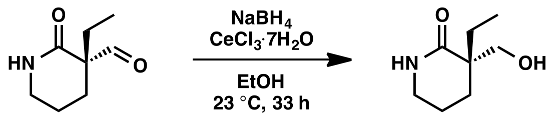

64 (82% yield) 65

Alcohol 65. To a suspension of $NaBH_4$ (49.4 mg, 1.3 mmol, 5.0 equiv) and $CeCl_3$•$7H_2O$ (146 mg, 0.39 mmol, 1.5 equiv) in EtOH (4.0 mL) was added at 0 °C solid **64** (40.5 mg, 0.26 mmol, 1.0 equiv) in one portion, after evolution of hydrogen gas had subsided. After stirring for 33 h at 23 °C, the heterogeneous reaction mixture was quenched with sat. NH_4Cl (15 mL) and diluted with CH_2Cl_2 (20 mL). The layers were separated and the aqueous layer was extracted with CH_2Cl_2 (20 mL). Since phase separation was tedious due to the presence of boronic acid salts (emulsion), the aqueous layer was then basified with 6 M NaOH (2 mL) and was extracted with CH_2Cl_2 (2 x 20 mL). The combined organic extracts were dried over $MgSO_4$ and the solvent was removed under reduced pressure. The residue was purified by column chromatography (hexane/acetone, 1:1 → 1:2) to afford **65** (33.8 mg, 82%) as a colorless, viscous oil. R_f = 0.33 (hexane/acetone, 1:2); ^{1}H NMR (500 MHz, $CDCl_3$): δ 6.66 (brs, 1H), 3.85 (brs, 1H), 3.59–3.45 (m, 2H), 3.32–3.20 (m, 2H), 1.89–1.64 (m, 5H), 1.47 (ddd, *J* = 13.7 Hz, 10.0 Hz, 3.6 Hz, 1H), 0.88 (t, *J* = 7.5 Hz, 3H); ^{13}C NMR (126 MHz, $CDCl_3$): δ 178.3, 67.4, 45.3, 42.2, 26.9, 26.5, 19.4, 7.9. IR (NaCl): 3289, 2939, 2875, 1643, 1492, 1355, 1324, 1208, 1055 cm^{-1}; HRMS (MM: ESI-APCI+) m/z calc'd for $C_8H_{15}NO_2$ $[M+H]^+$: 158.1176, found 158.1179. $[\alpha]_D^{24}$ –12.9° (*c* 1.69, $CHCl_3$) (Lit.[9] for (*R*)-**65**: $[\alpha]_D^{27}$ +13.5° (*c* 1.05, $CHCl_3$)).

During the synthesis of the racemic compound, alcohol (±)-**65** solidified upon storage in the freezer to give a white crystalline solid. X-ray quality crystals were obtained by slow diffusion of heptane (with a few drops of benzene) into a solution of the alcohol in EtOAc at 23 °C. X-ray structure has been deposited in the Cambridge Database (CCDC) under the deposition number 1002339.

mp: 91–93 °C.

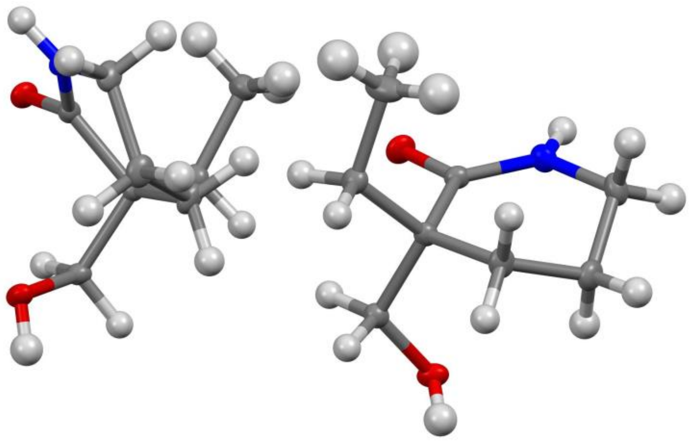

Figure 2: Crystal structure of (±)-**65** (ellipsoids, 50% probability level).

Methods for the Determination of Enantiomeric Excess

entry	product	assay conditions	retention time of major isomer (min)	retention time of minor isomer (min)	% ee
1	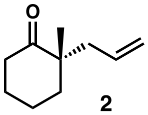 **2**	Chiral GC<br>Agilent GT-A<br>100 °C isotherm	11.1	12.7	88
2	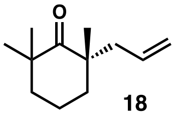 **18**	Chiral GC<br>Agilent GT-A<br>80 °C isotherm	29.1	30.5	91
3	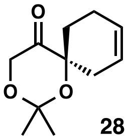 **28**	HPLC<br>Chiralpak AD<br>Hexanes<br>isocratic, 1.0 mL/min<br>220 nm	11.462	10.307	92
4	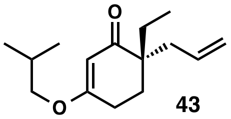 **43**	HPLC<br>Chiralcel OD<br>2% EtOH in hexanes<br>isocratic, 1.0 mL/min<br>254 nm	7.4	8.2	86
5	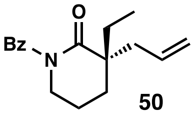 **50**	SFC<br>Chiralcel OJ-H<br>3% MeOH in CO_2<br>isocratic, 5.0 mL/min<br>254 nm	3.85	2.49	99

References

(1) (a) Peer, M.; de Jong, J. C.; Kiefer, M.; Langer, T.; Riech, H.; Schell, H.; Sennhenn, P.; Sprinz, J.; Steinhagen, H.; Wiese, B.; Helmchen, G. *Tetrahedron* **1996**, *52*, 7547–7583. (b) Tani, K.; Behenna, D. C.; McFadden, R. M.; Stoltz, B. M. *Org. Lett.* **2007**, *9*, 2529–2931.

(2) When a subscript is shown with the coupling constant, it indicates what type of splitting the constant is associated with. For example (td, J_t = 5.0 Hz, J_d = 3.3 Hz, 1H) indicates that the triplet splitting has a 5.0 Hz coupling constant and the doublet has a 3.3 Hz coupling constant.

(3) McFadden, R. M.; Stoltz, B. M. *J. Am. Chem. Soc.* **2006**, *128*, 7738–7739.

(4) Seto, M.; Roizen, J. L.; Stoltz, B. M. *Angew. Chem., Int. Ed.* **2008**, *47*, 6873–6876.

(5) Behenna, D. C.; Stoltz, B. M. *J. Am. Chem. Soc.* **2004**, *126*, 15044–15045.

(6) Das, P. P.; Lysenko, I. L.; Cha, J. K. *Angew. Chem., Int. Ed.* **2011**, *50*, 9459–9461.

(7) Behenna, D. C.; Liu, Y.; Yurino, T.; Kim, J.; White, D. E.; Virgil, S. C.; Stoltz, B. M. *Nature Chem.* **2012**, *4*, 130–133.

(8) Arisawa, M.; Terada, Y.; Nakagawa, M.; Nishida, A. *Angew. Chem., Int. Ed.* **2002**, *41*, 4732–4734.

(9) Pandey, G.; Kumara, P. C. *Org. Lett.* **2011**, *13*, 4672–4675.

^{1}H and ^{13}C NMR Spectra

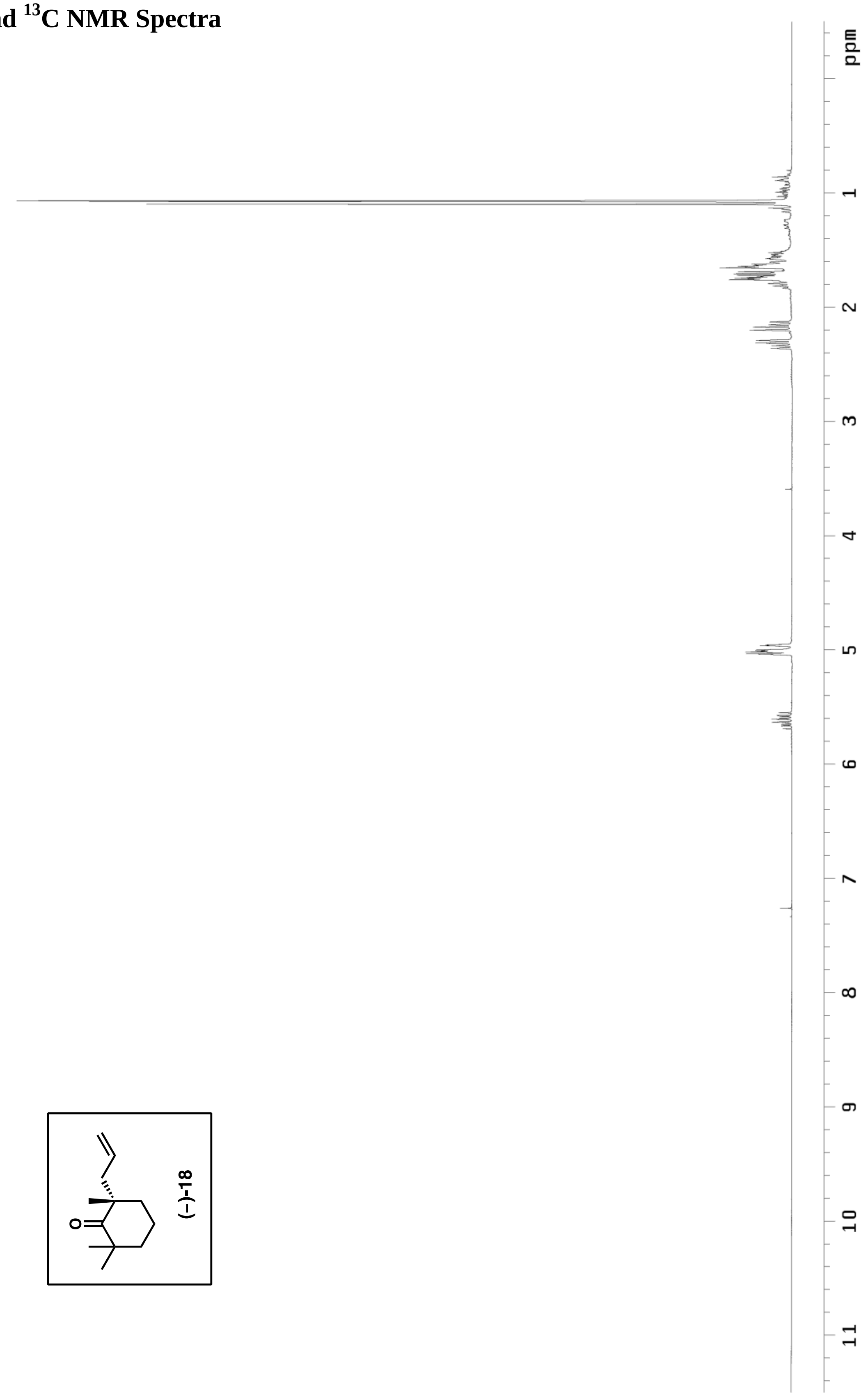

^{1}H NMR (300 MHz, $CDCl_3$) of compound **18**.

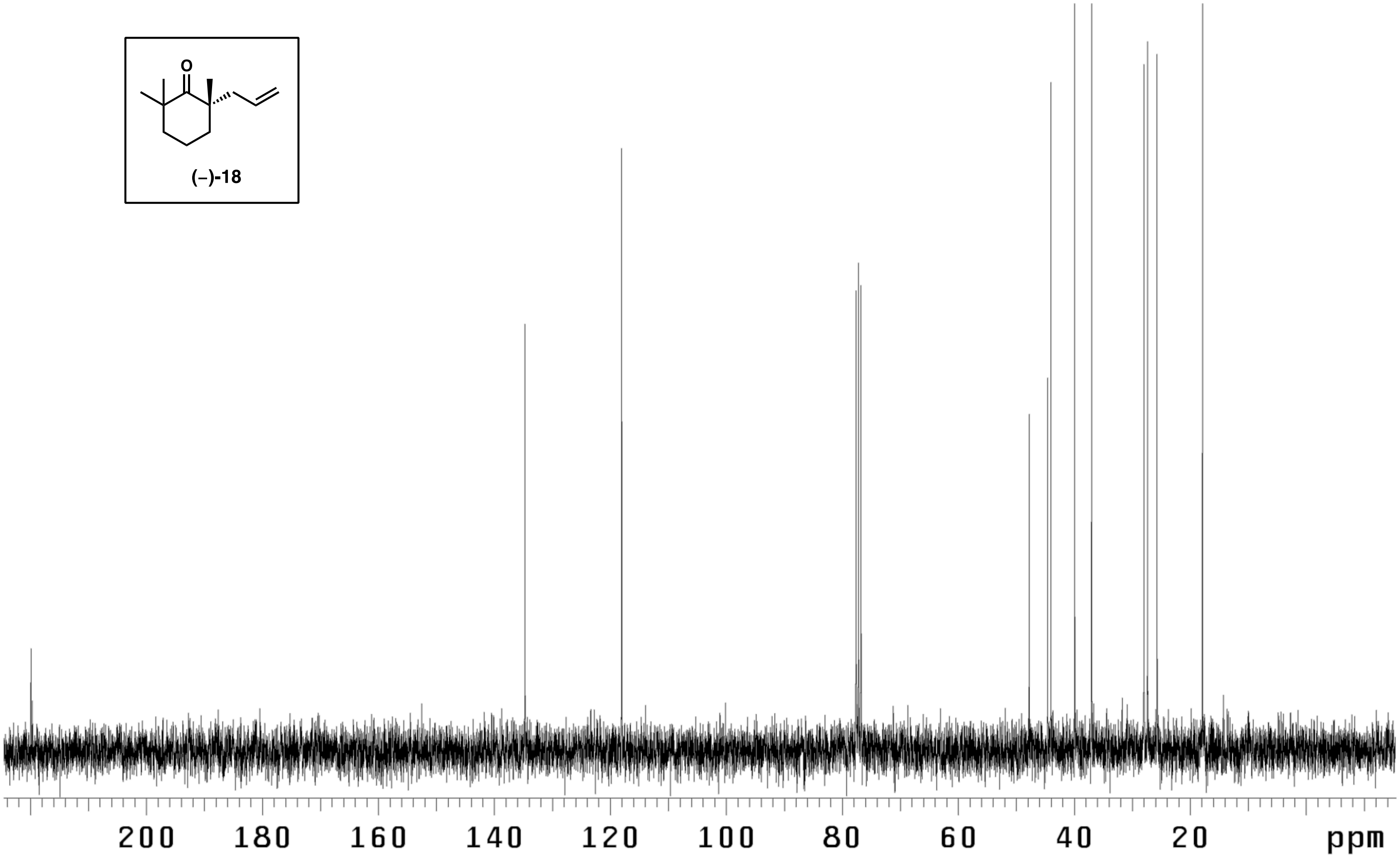

^{13}C NMR (75 MHz, $CDCl_3$) of compound **18**.

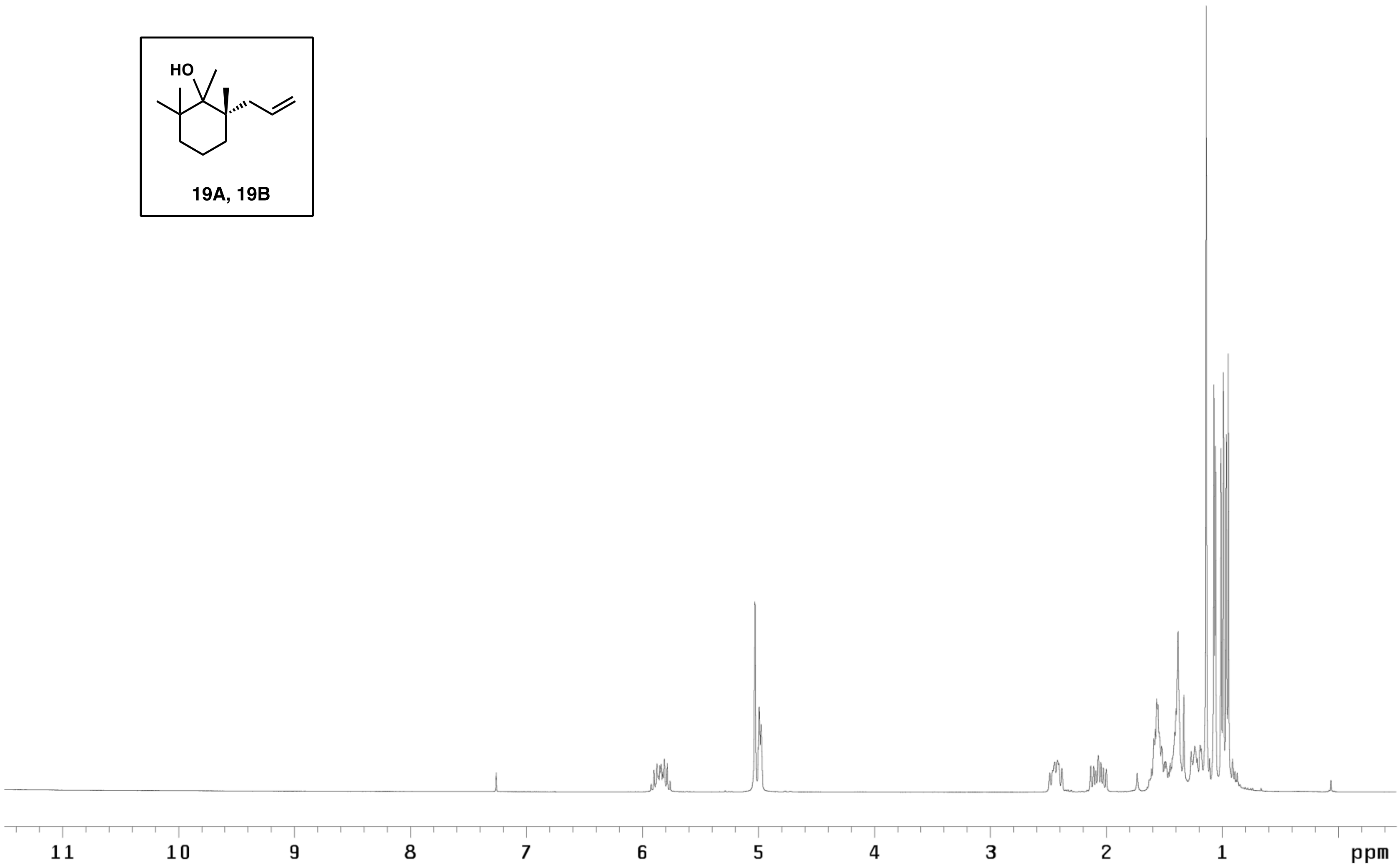

^{1}H NMR (300 MHz, $CDCl_3$) of compounds **19A** and **19B**.

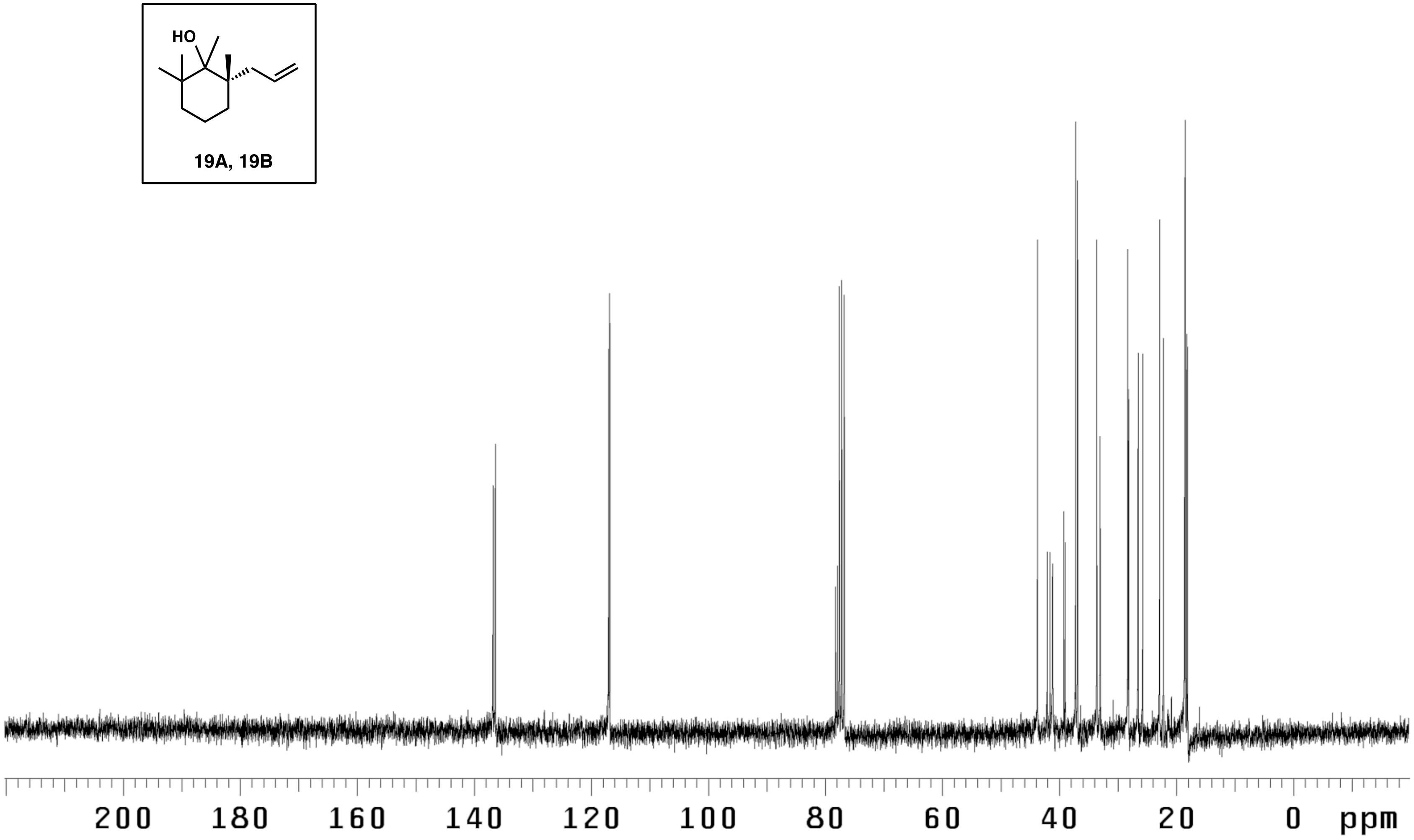

^{13}C NMR (75 MHz, $CDCl_3$) of compounds **19A** and **19B**.

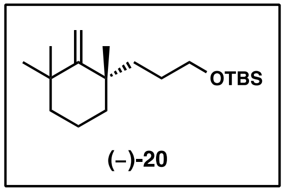

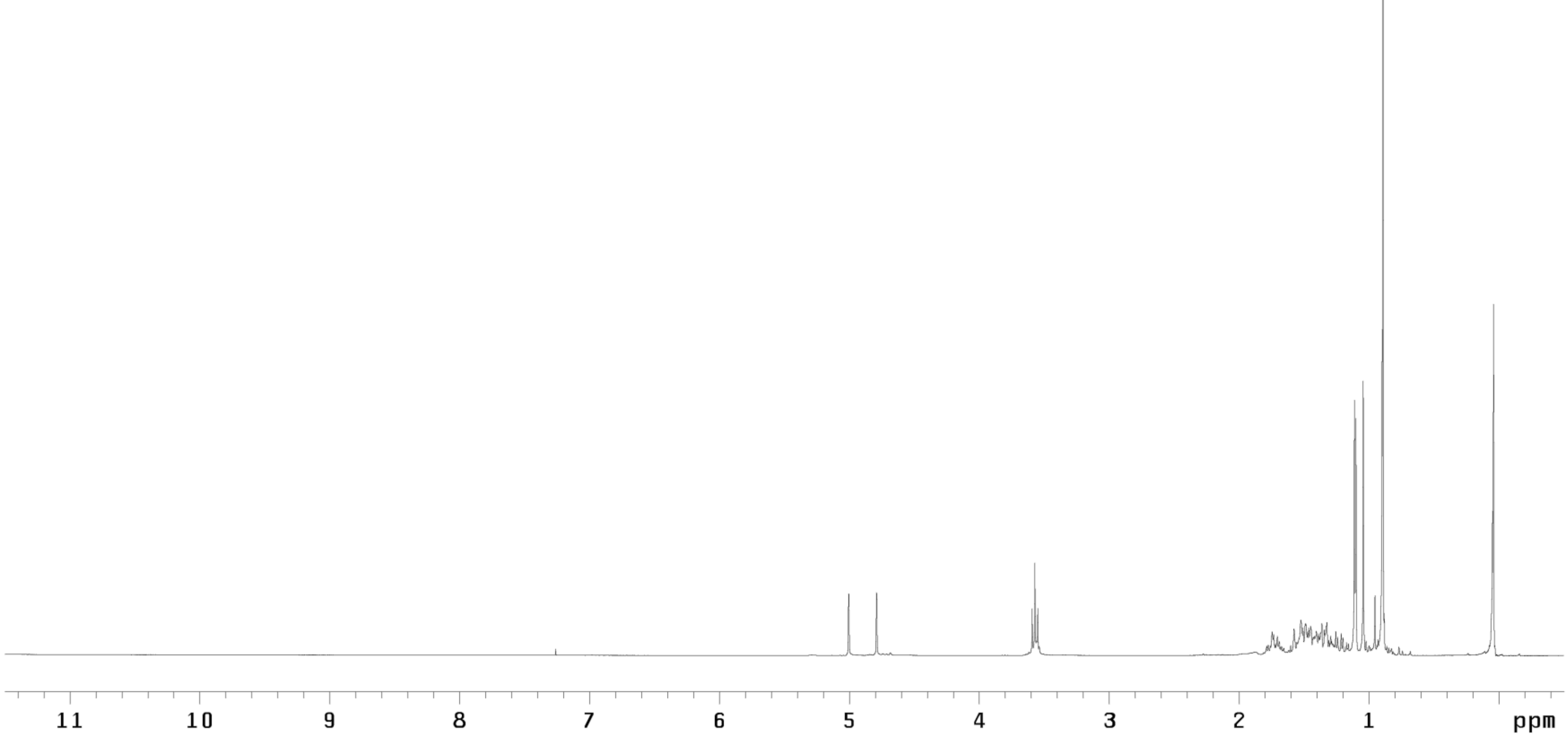

^{1}H NMR (300 MHz, $CDCl_3$) of compound **20**.

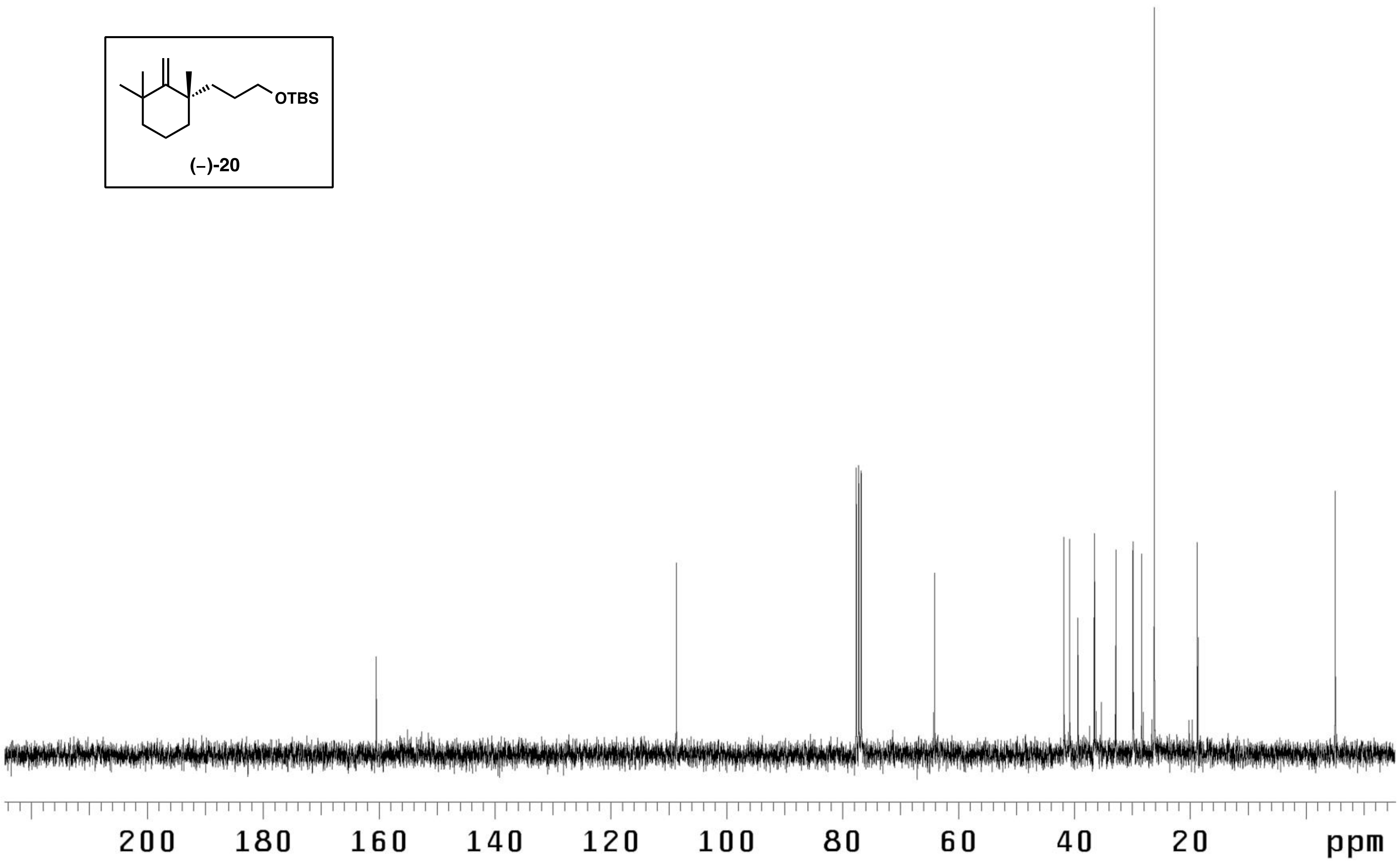

^{13}C NMR (75 MHz, $CDCl_3$) of compound **20**.

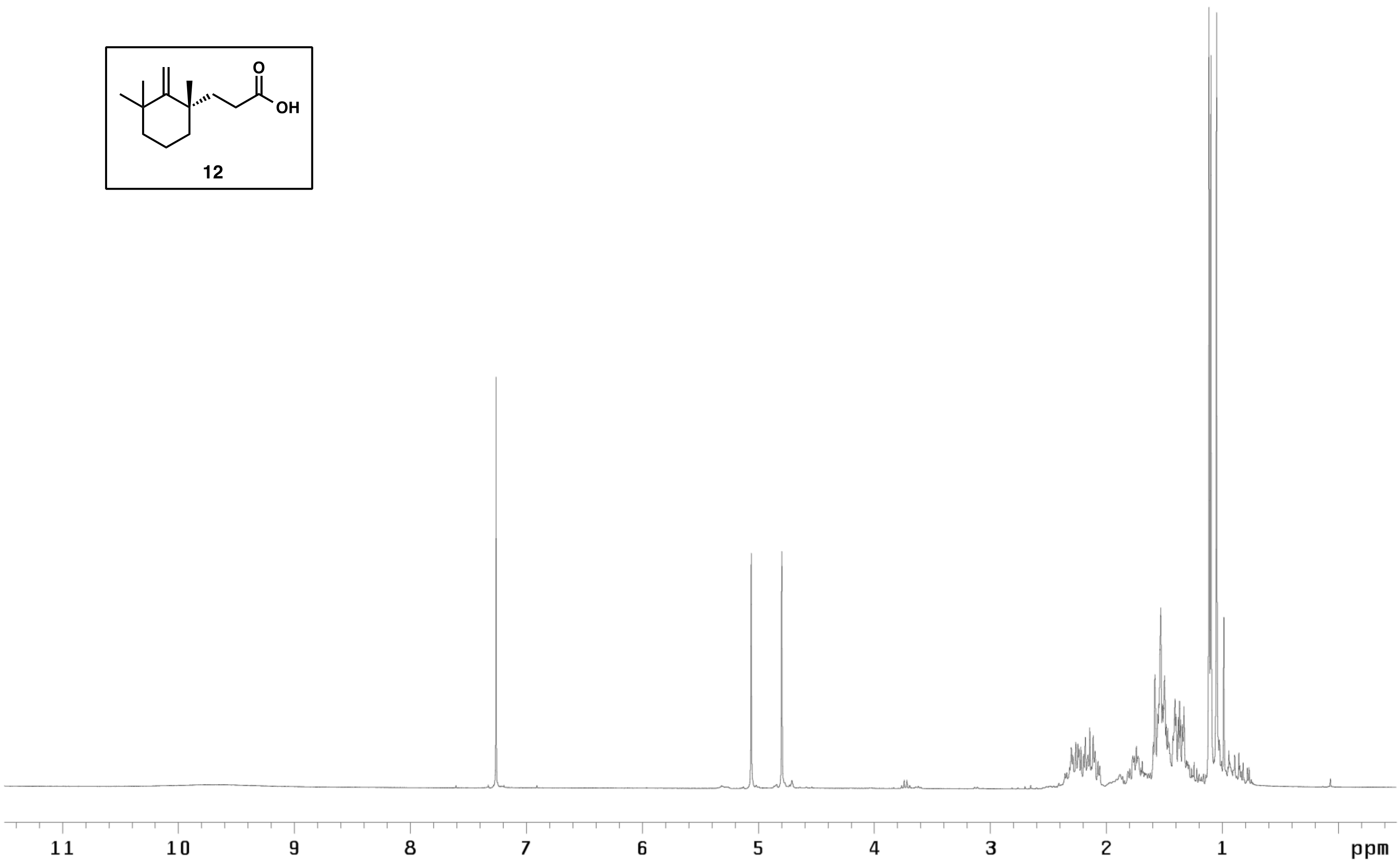

^{1}H NMR (300 MHz, $CDCl_3$) of compound **12**.

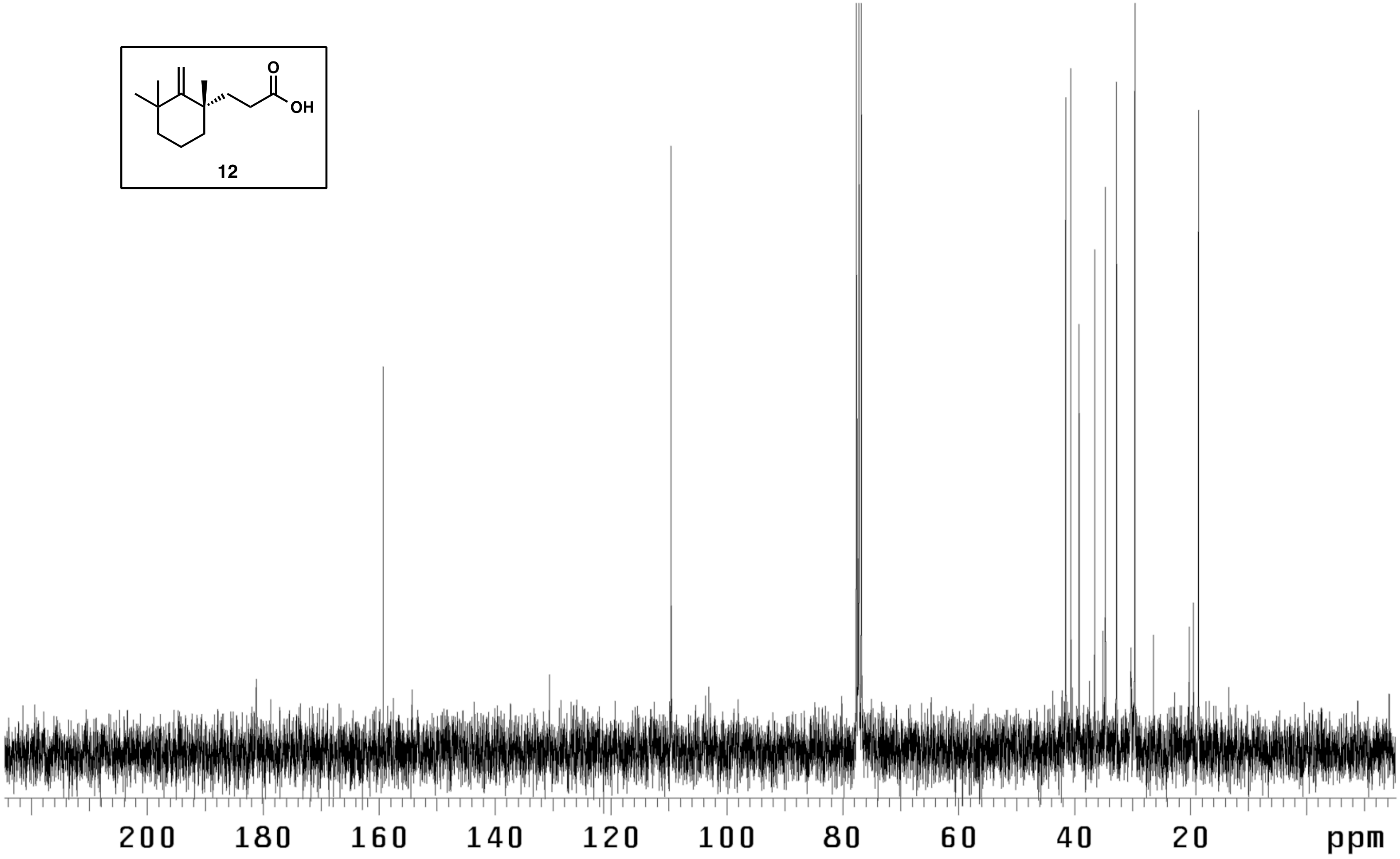

^{13}C NMR (75 MHz, $CDCl_3$) of compound **12**.

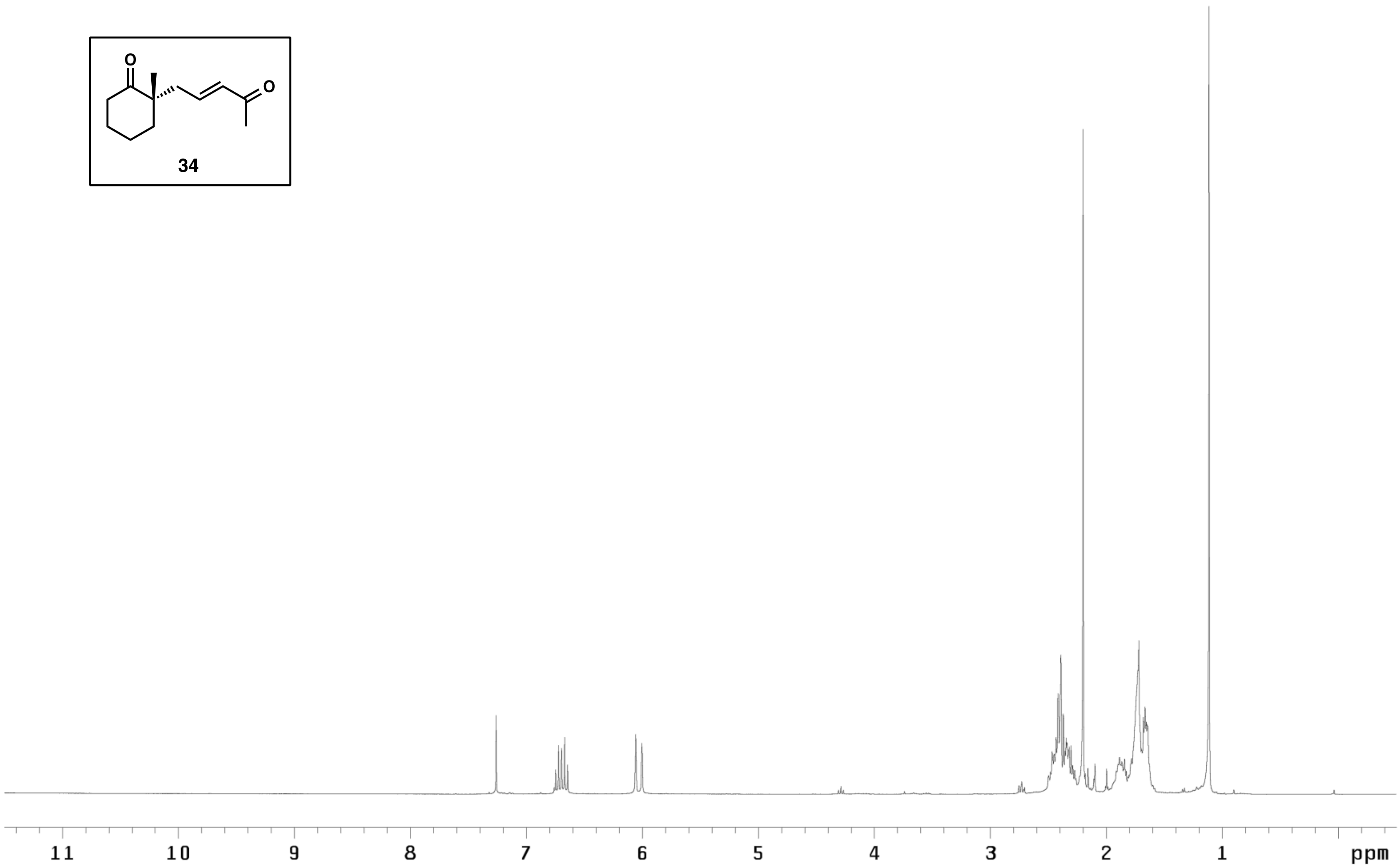

^{1}H NMR (300 MHz, $CDCl_3$) of compound **34**.

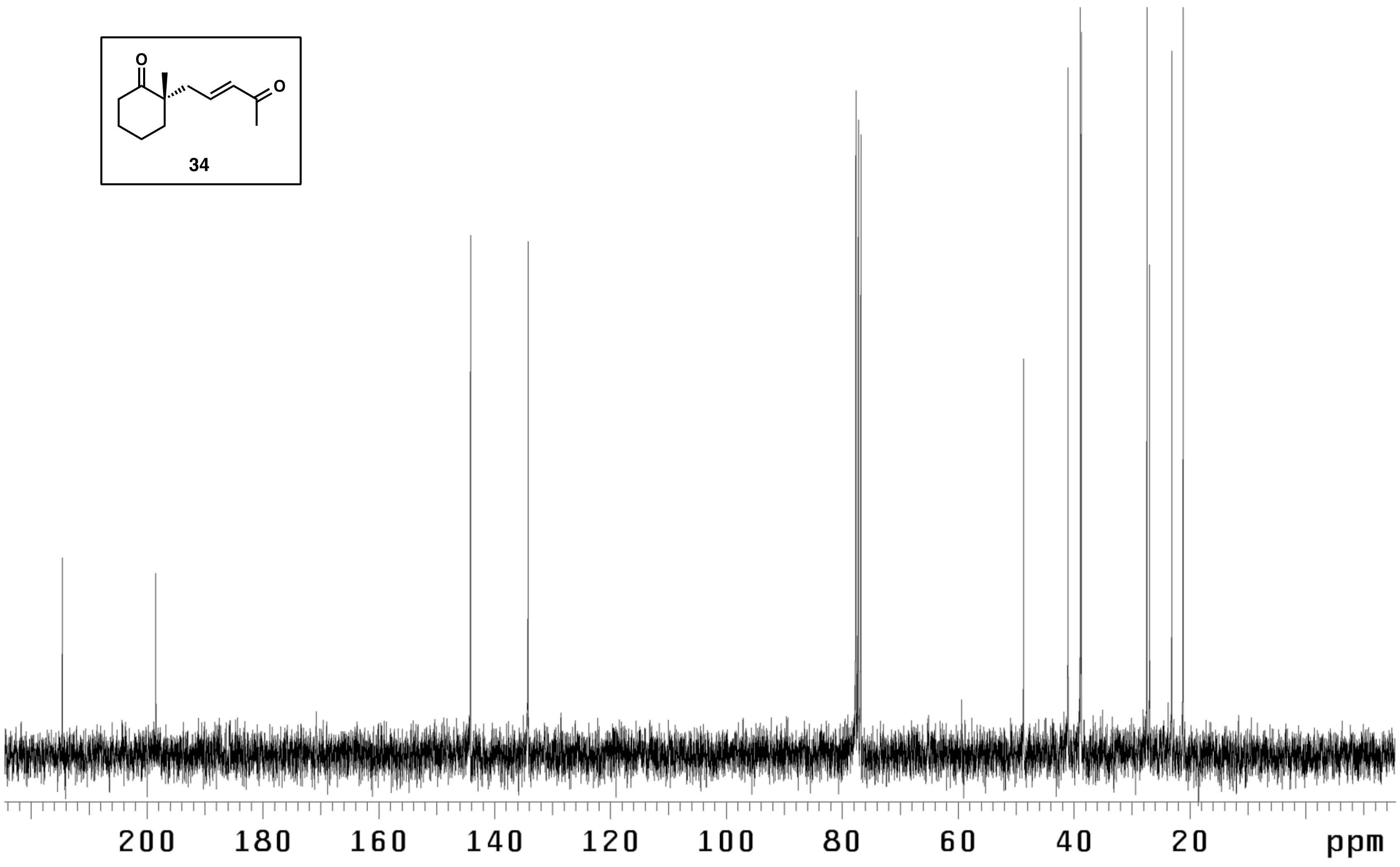

^{13}C NMR (75 MHz, $CDCl_3$) of compound **34**.

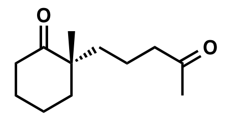

35

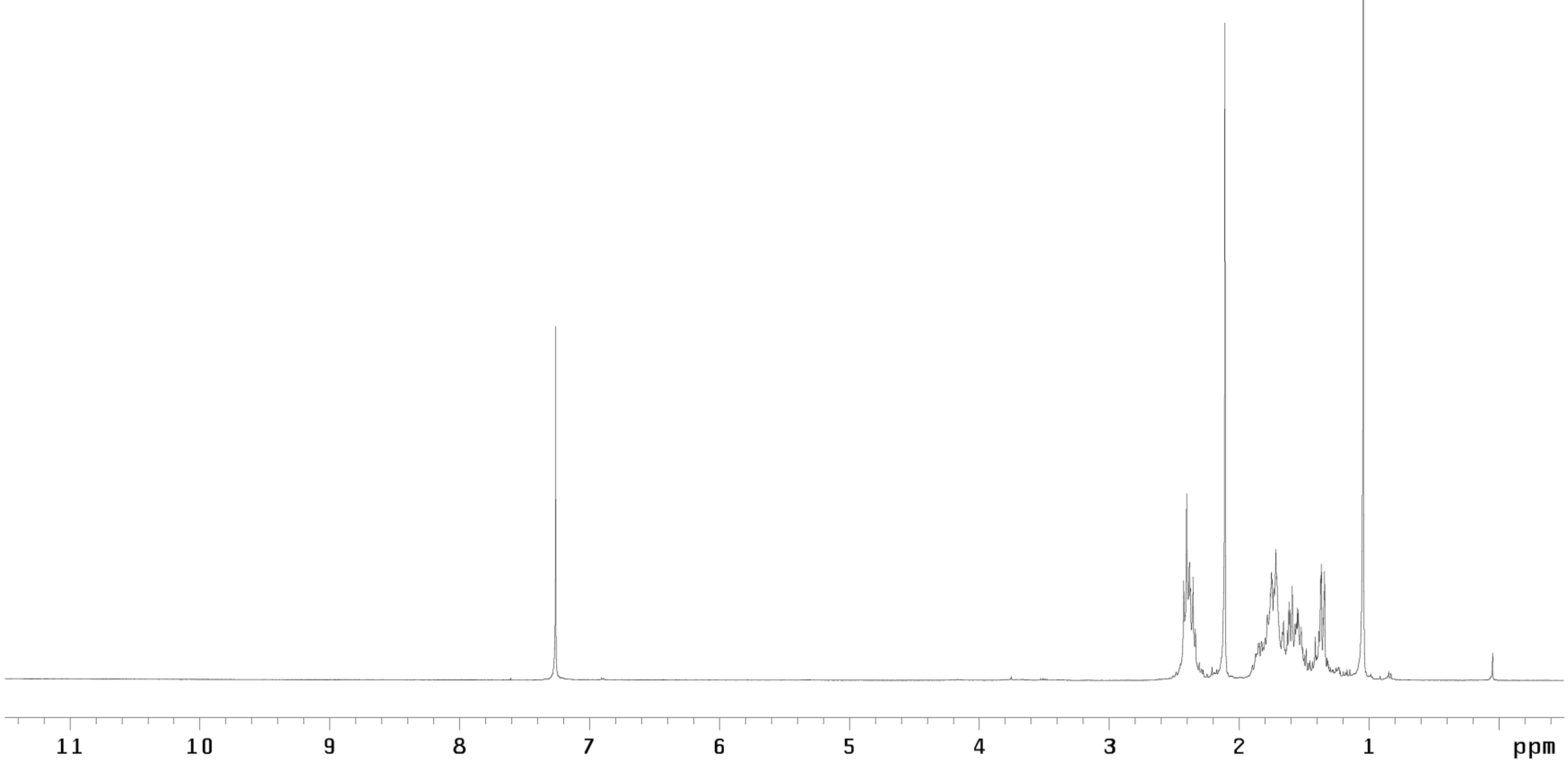

^{1}H NMR (300 MHz, $CDCl_3$) of compound **35**.

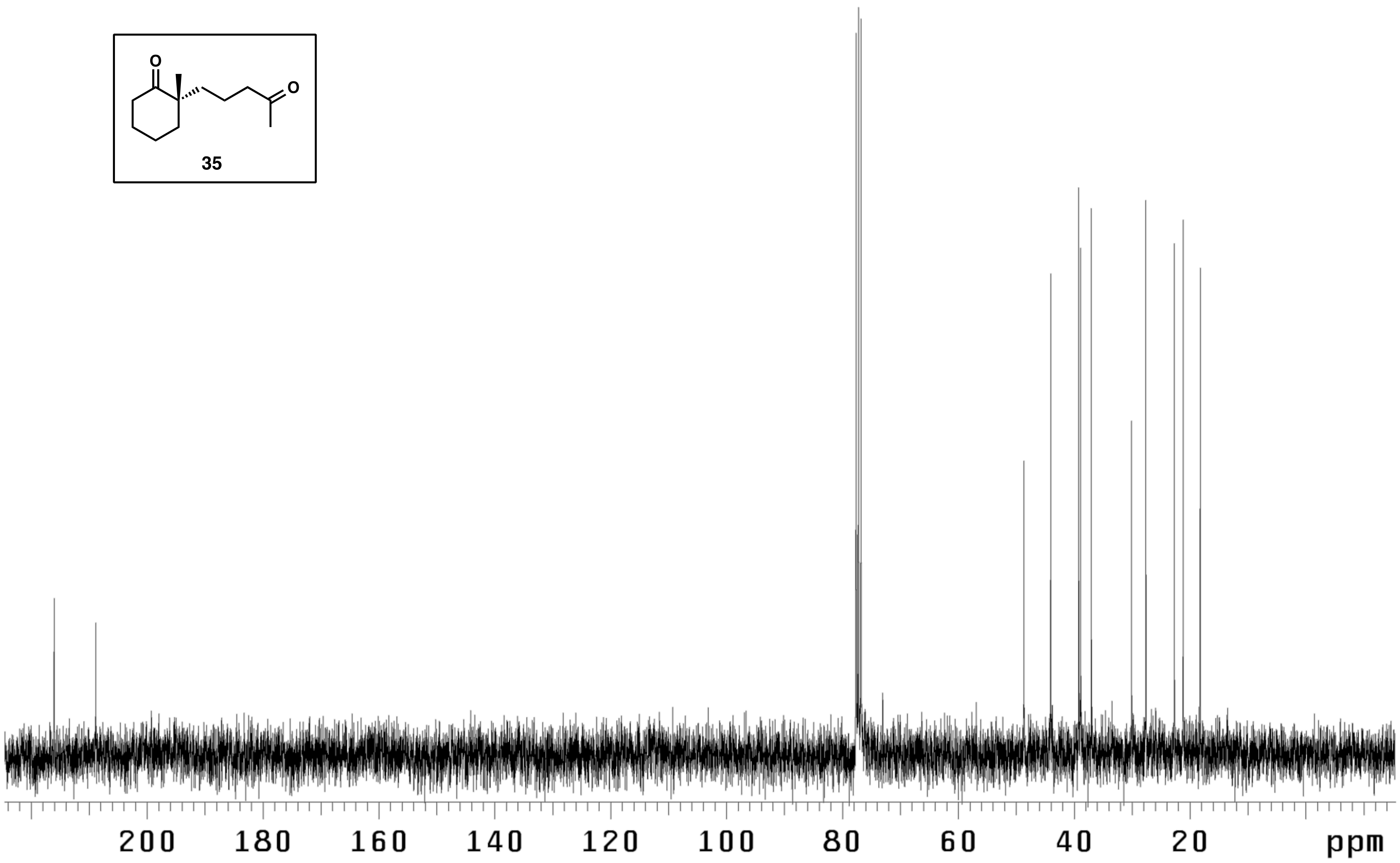

^{13}C NMR (75 MHz, $CDCl_3$) of compound **35**.

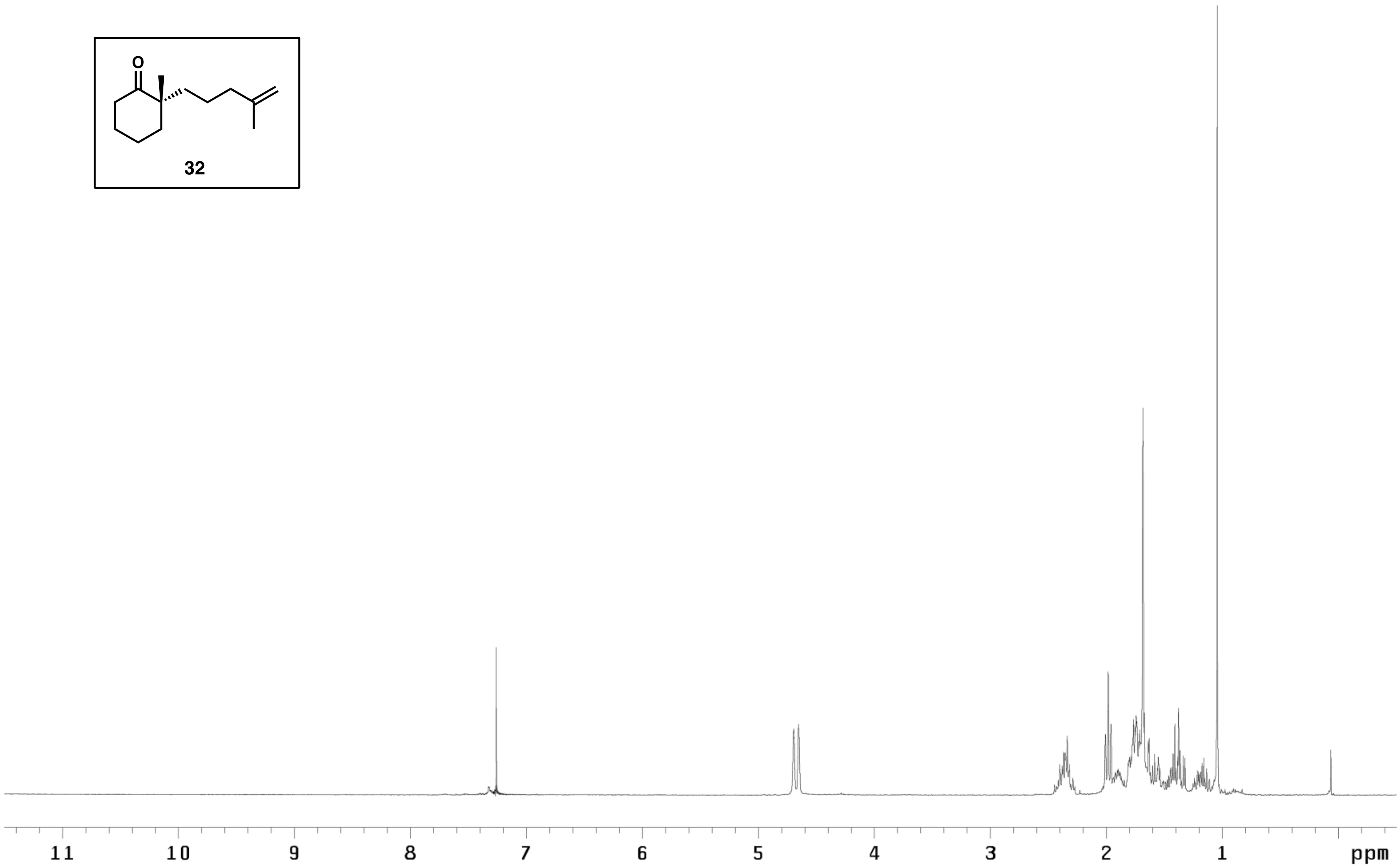

^{1}H NMR (300 MHz, $CDCl_3$) of compound **32**.

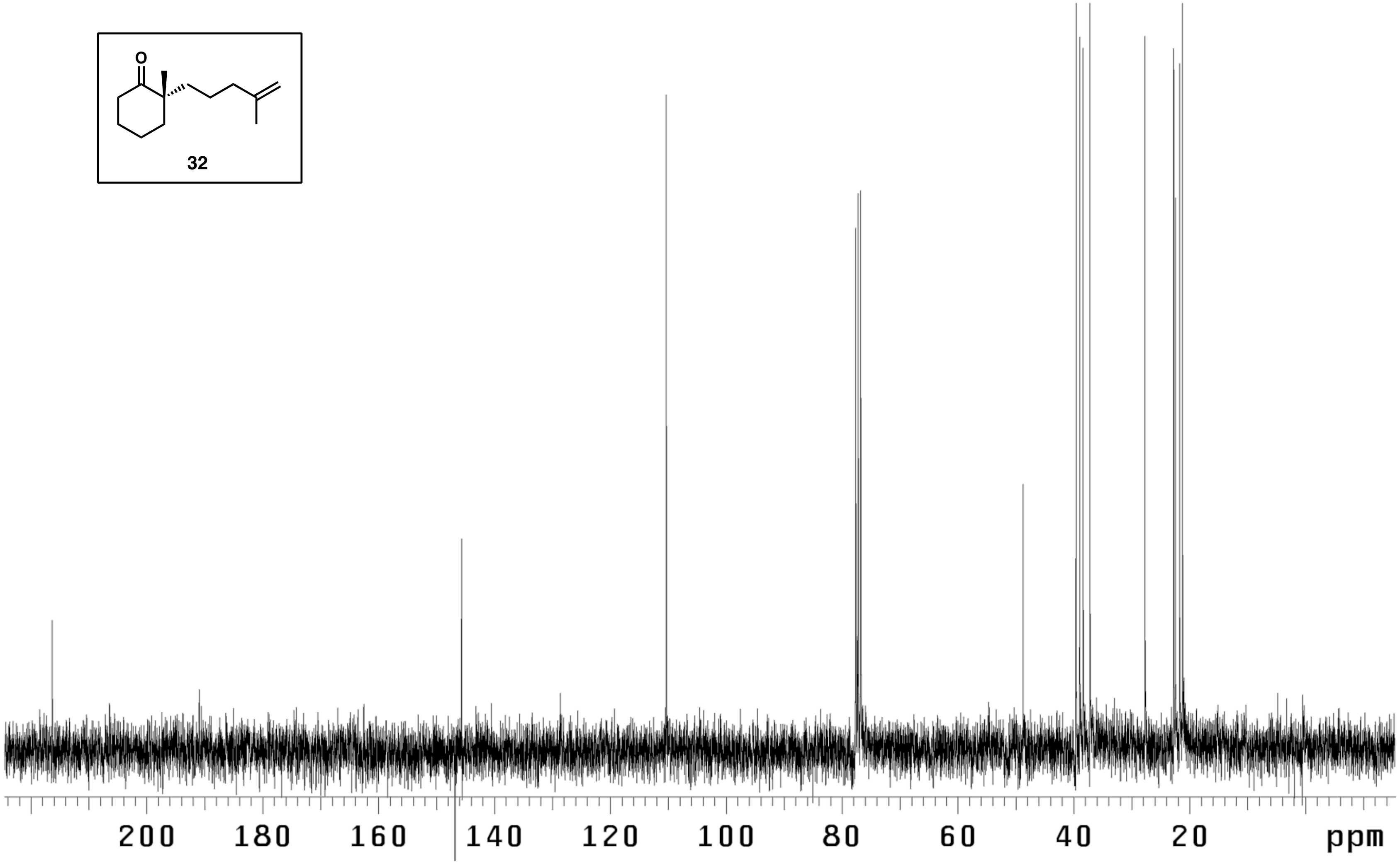

^{13}C NMR (75 MHz, $CDCl_3$) of compound **32**.

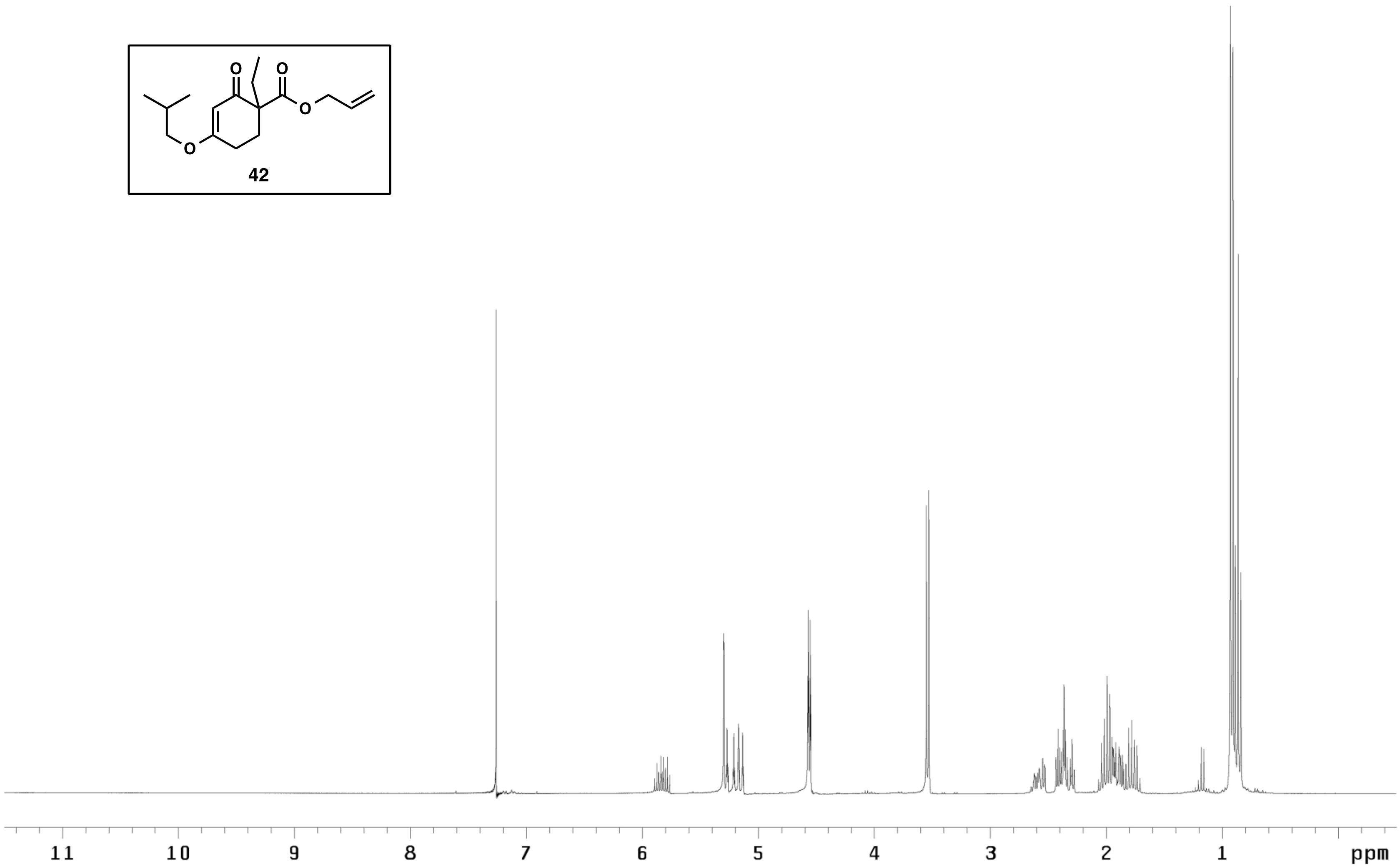

^{1}H NMR (300 MHz, $CDCl_3$) of compound **42**.

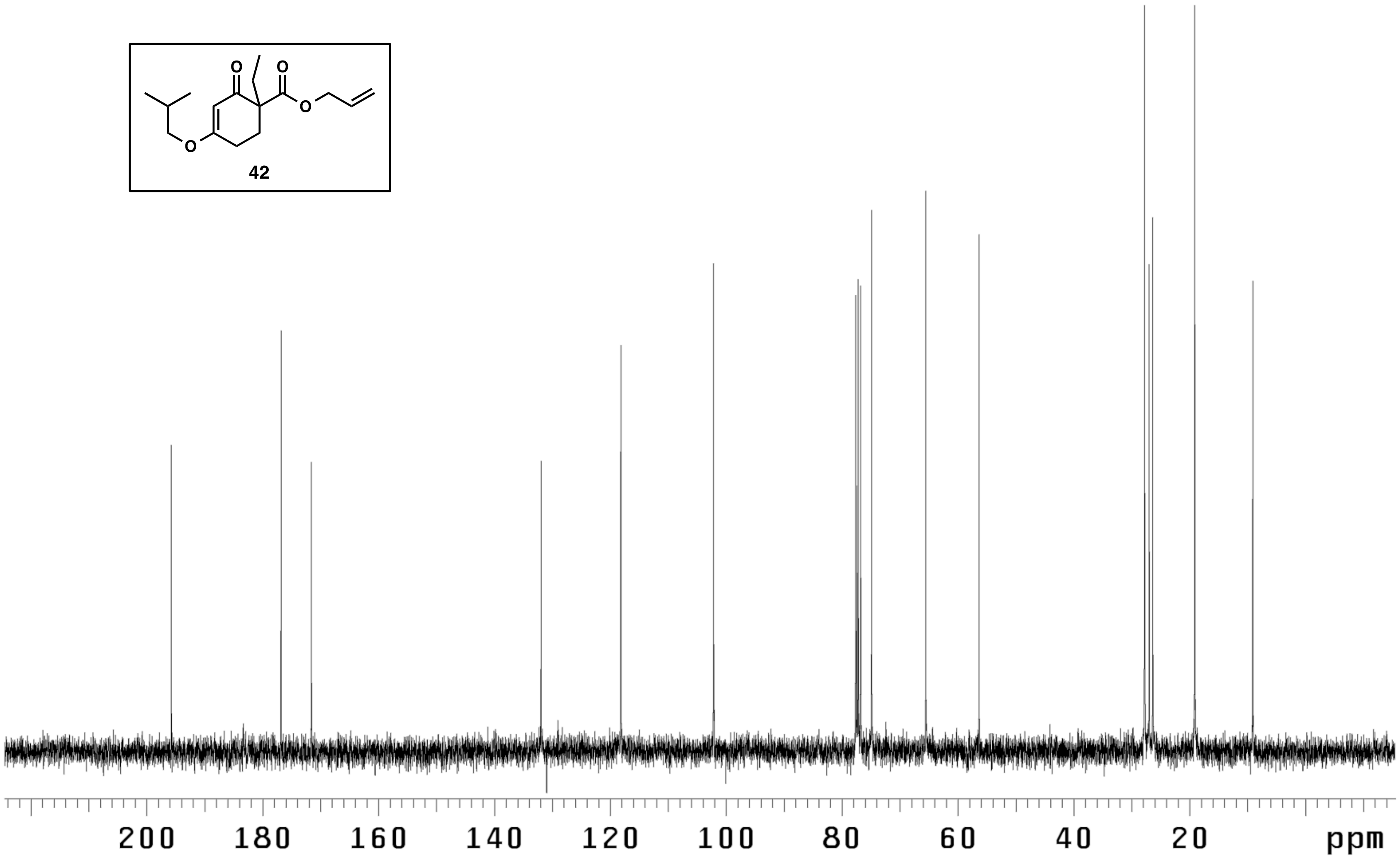

^{13}C NMR (75 MHz, $CDCl_3$) of compound **42**.

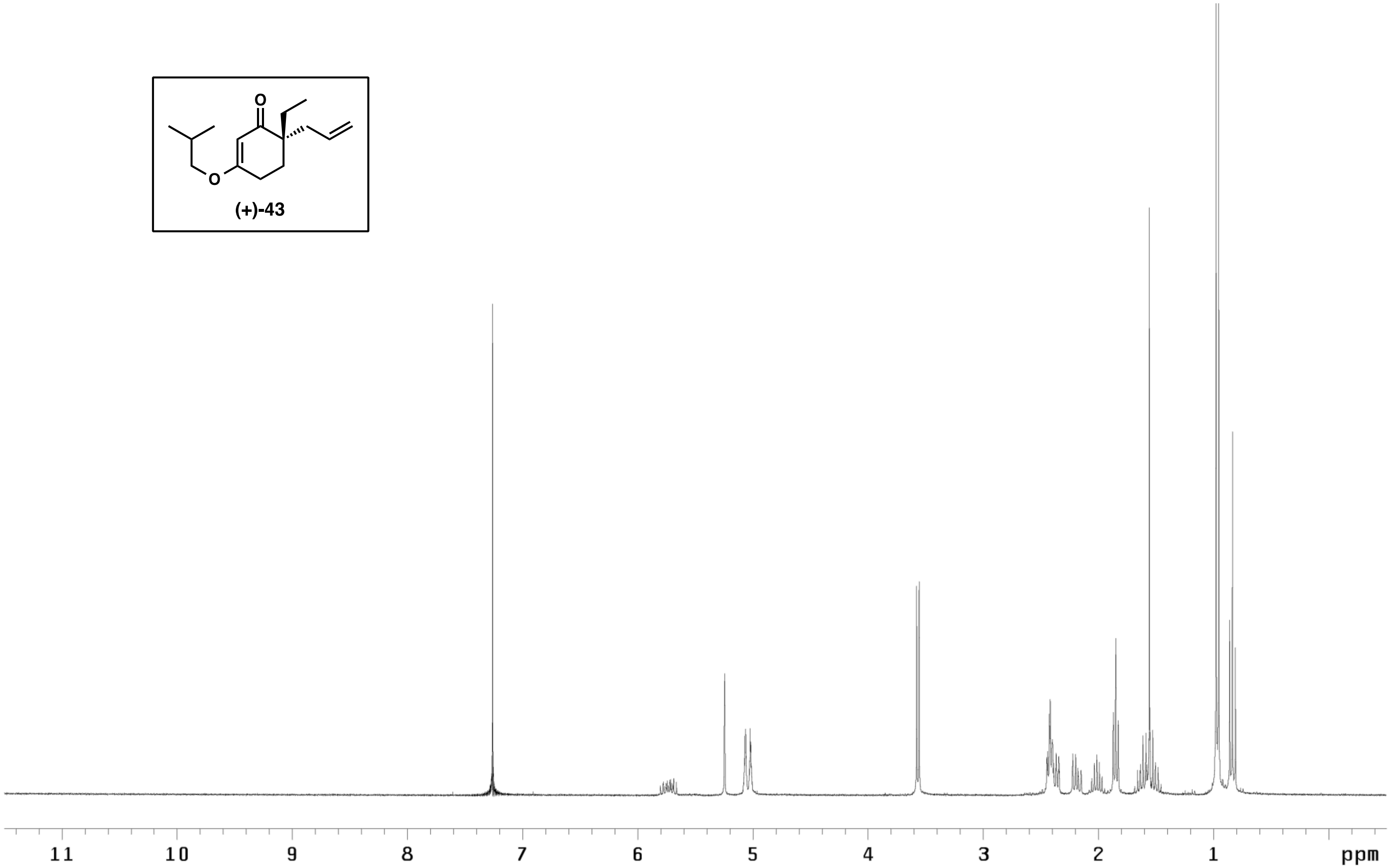

^{1}H NMR (300 MHz, $CDCl_3$) of compound **43**.

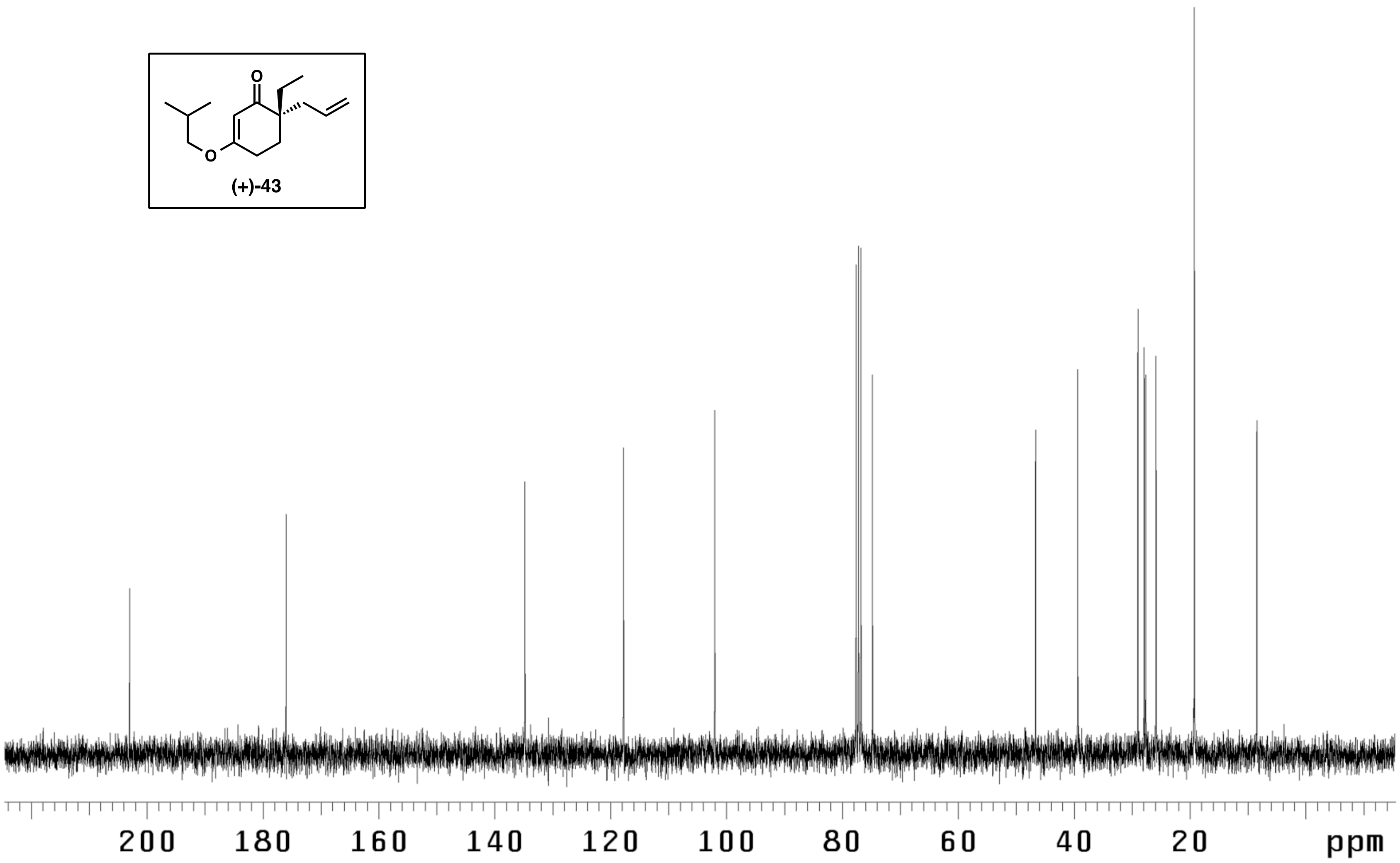

^{13}C NMR (75 MHz, $CDCl_3$) of compound **43**.

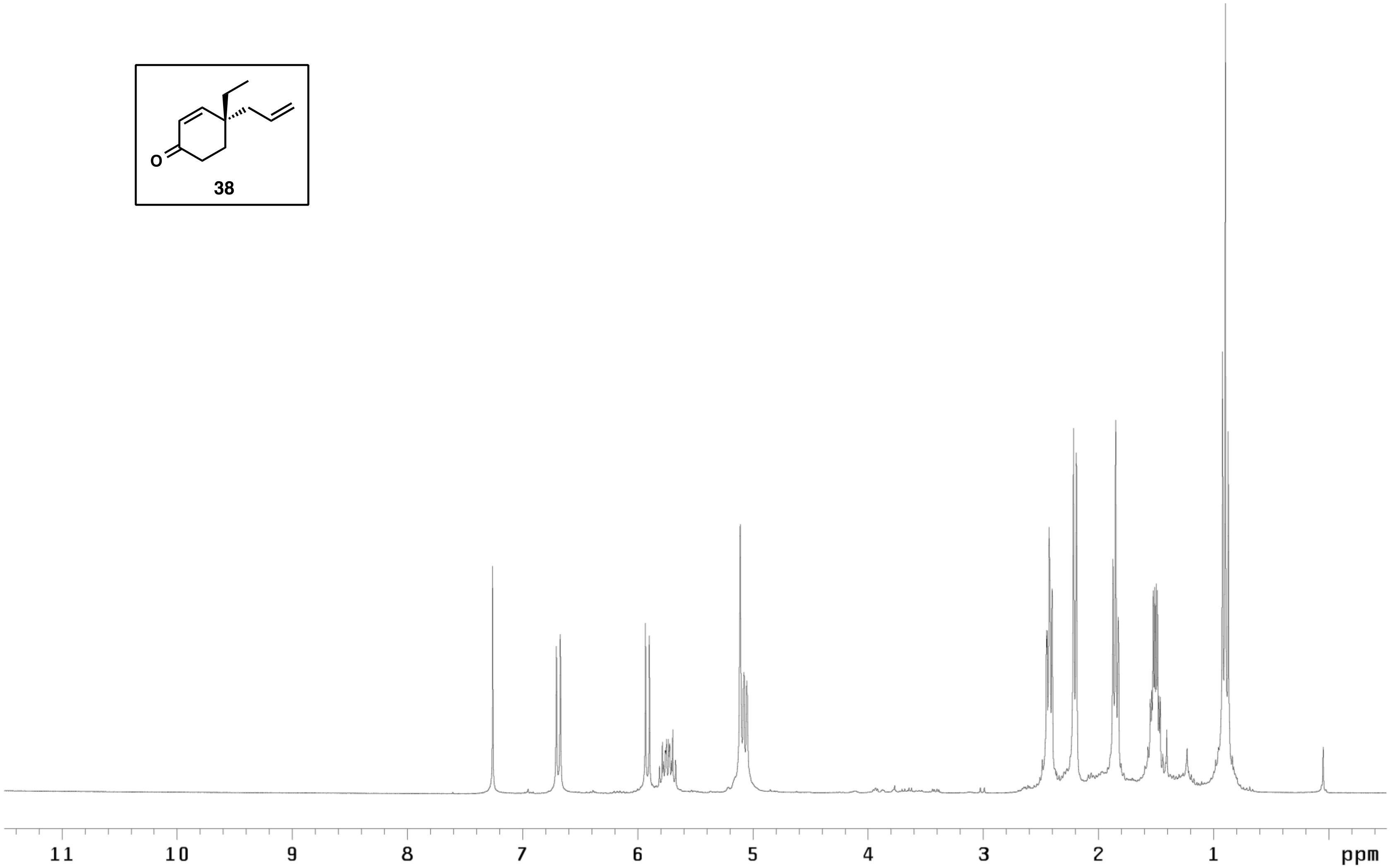

^{1}H NMR (300 MHz, $CDCl_3$) of compound **38**.

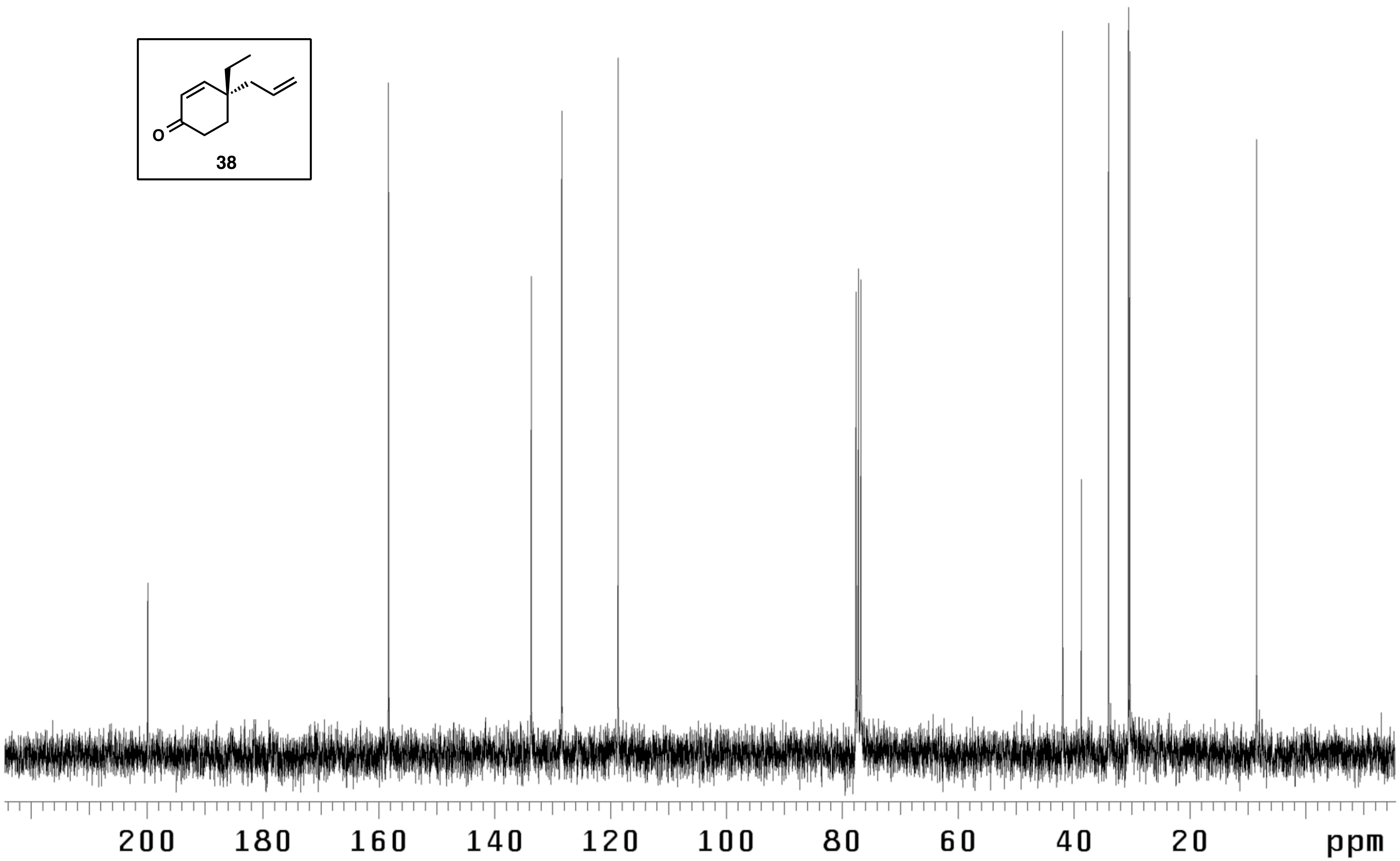

^{13}C NMR (75 MHz, $CDCl_3$) of compound **38**.

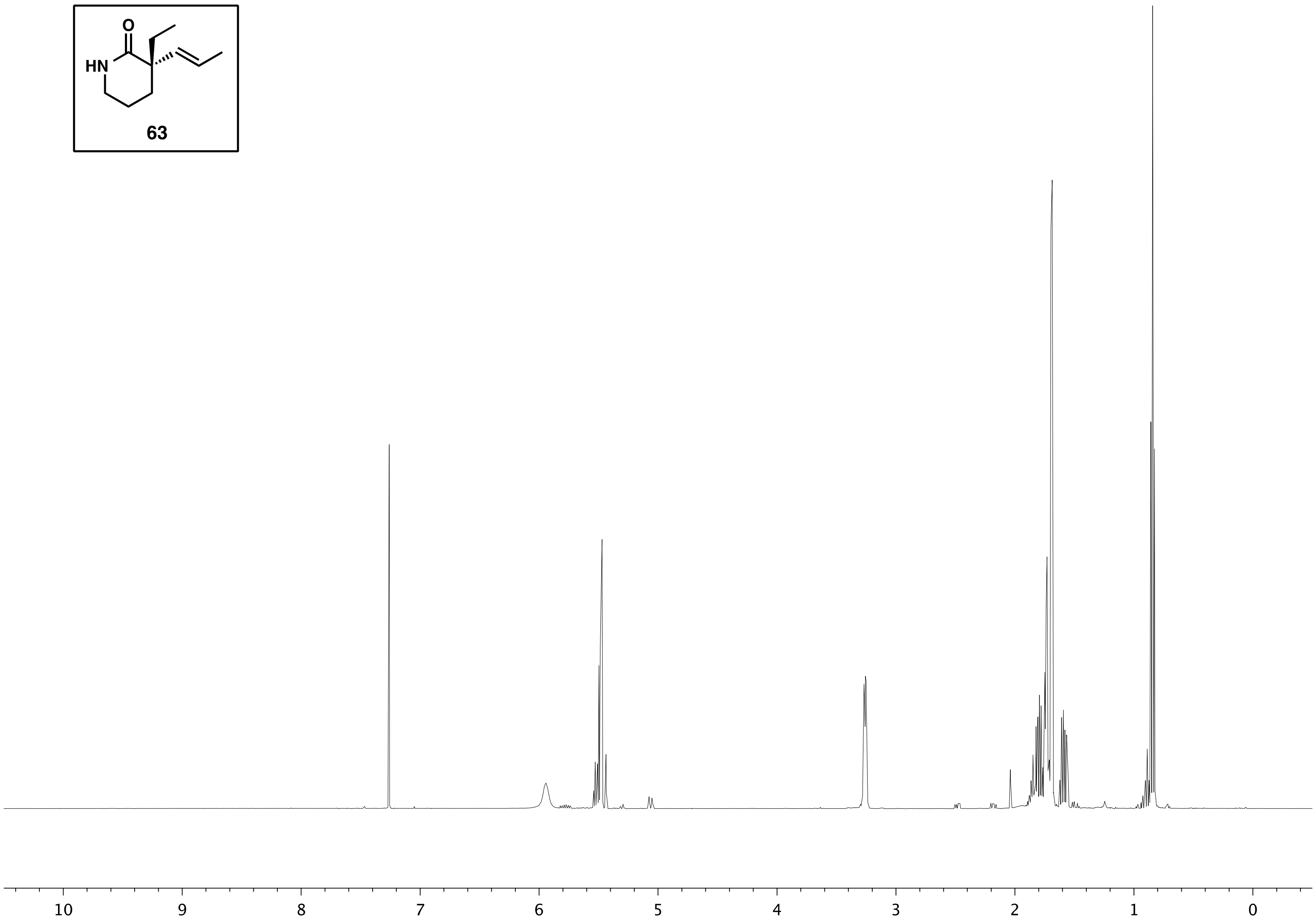

^{1}H NMR (500 MHz, $CDCl_3$) of compound **63**.

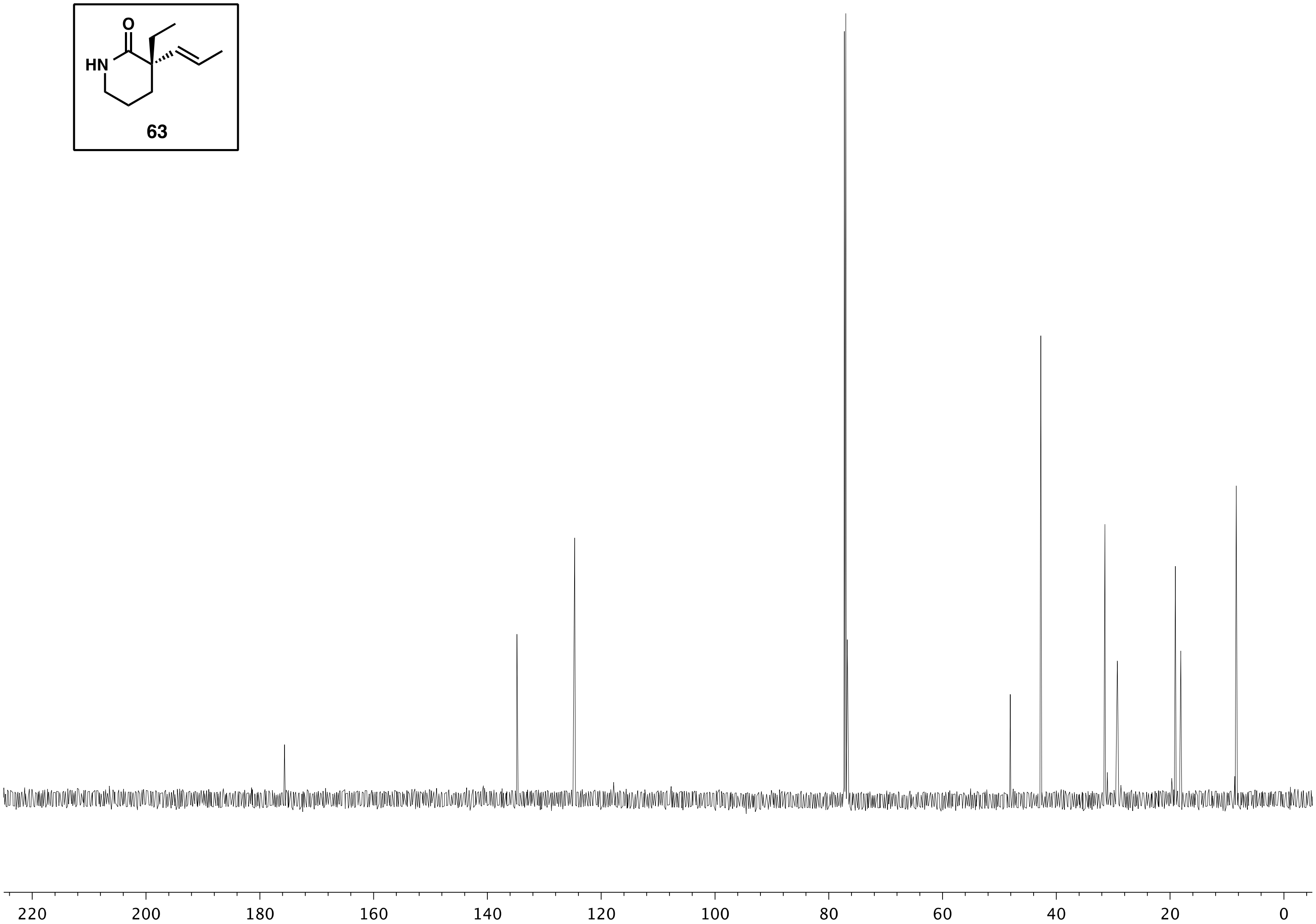

^{13}C NMR (126 MHz, $CDCl_3$) of compound **63**.

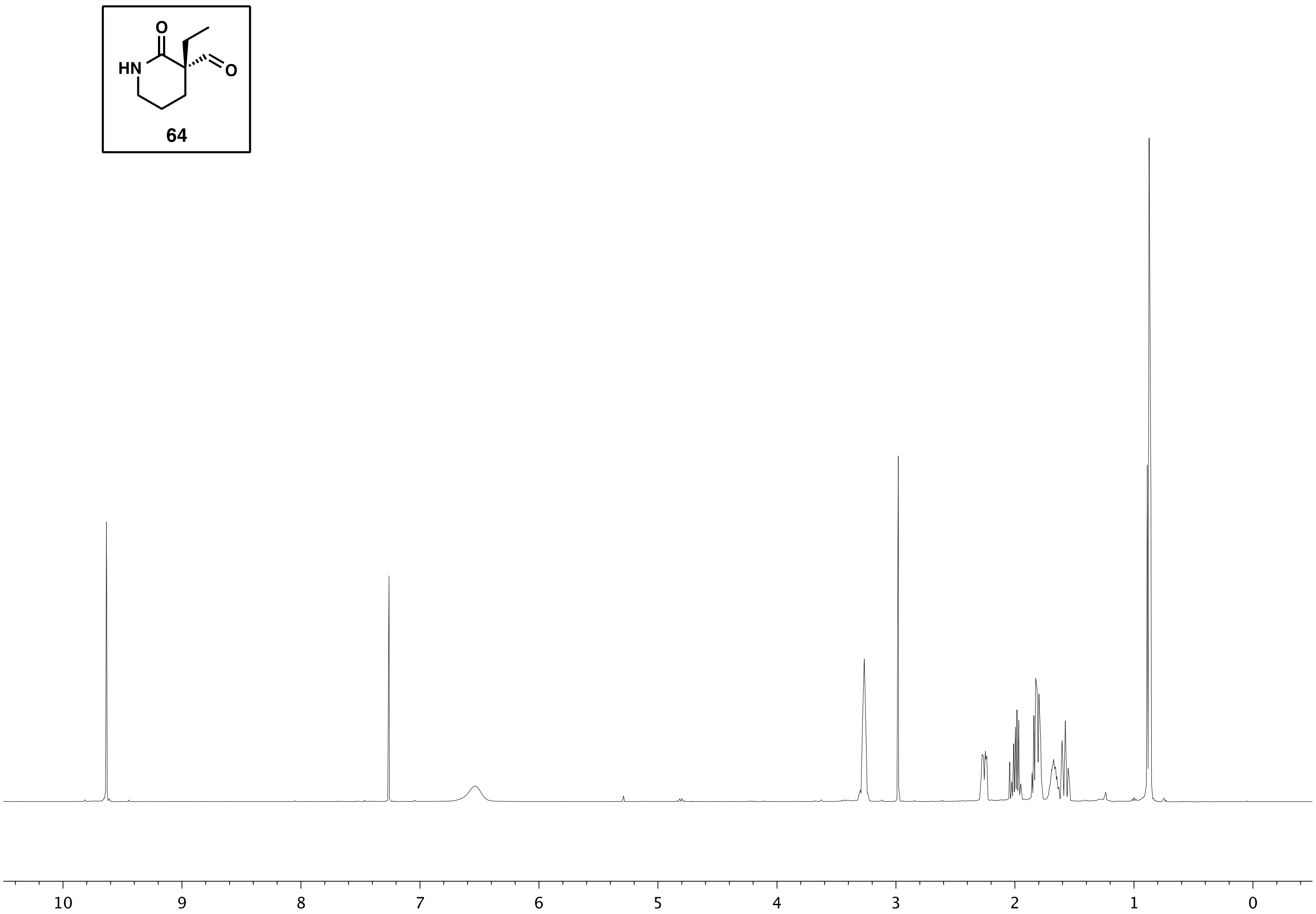

^{1}H NMR (500 MHz, $CDCl_3$) of compound **64**.

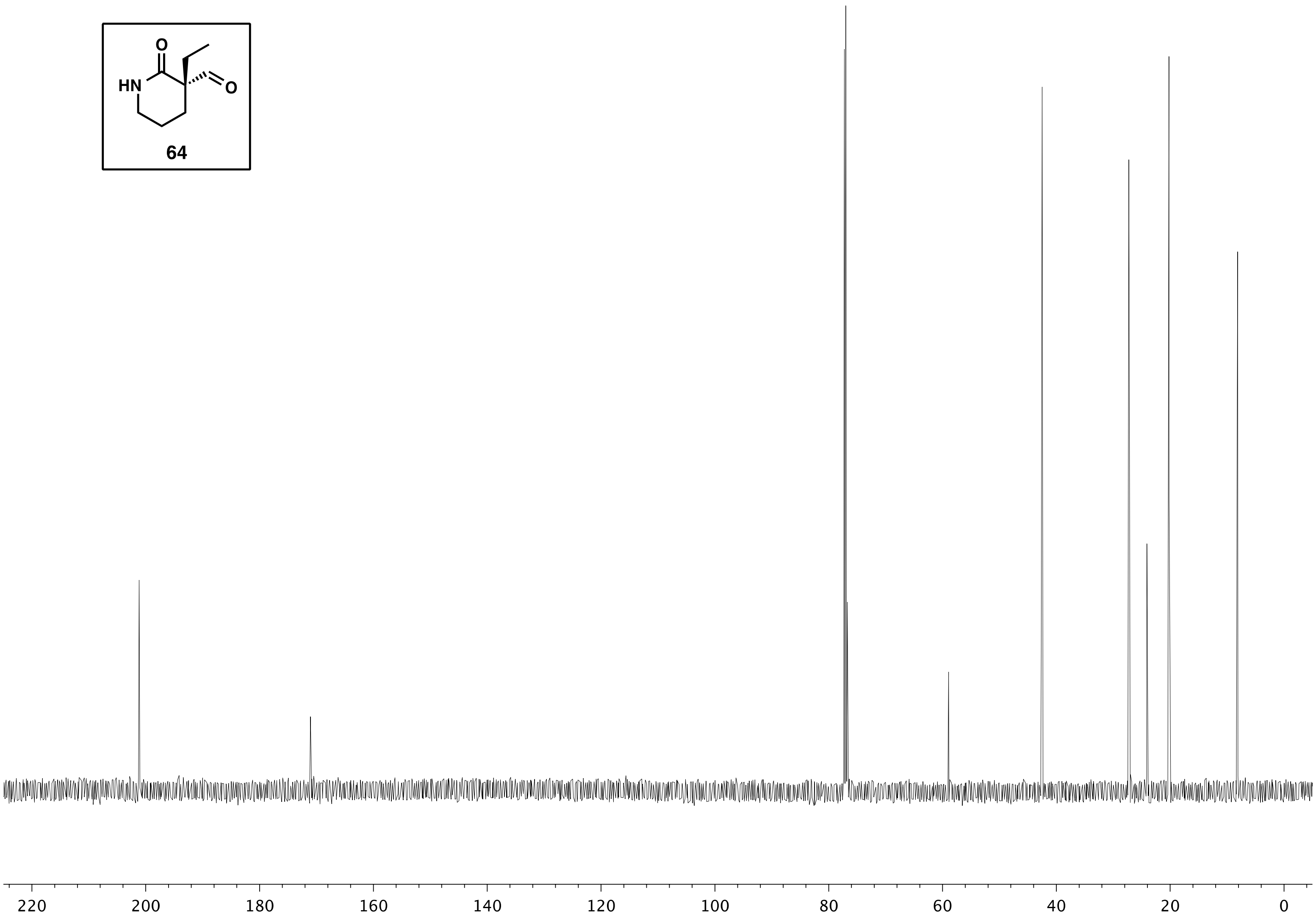

^{13}C NMR (126 MHz, $CDCl_3$) of compound **64**.

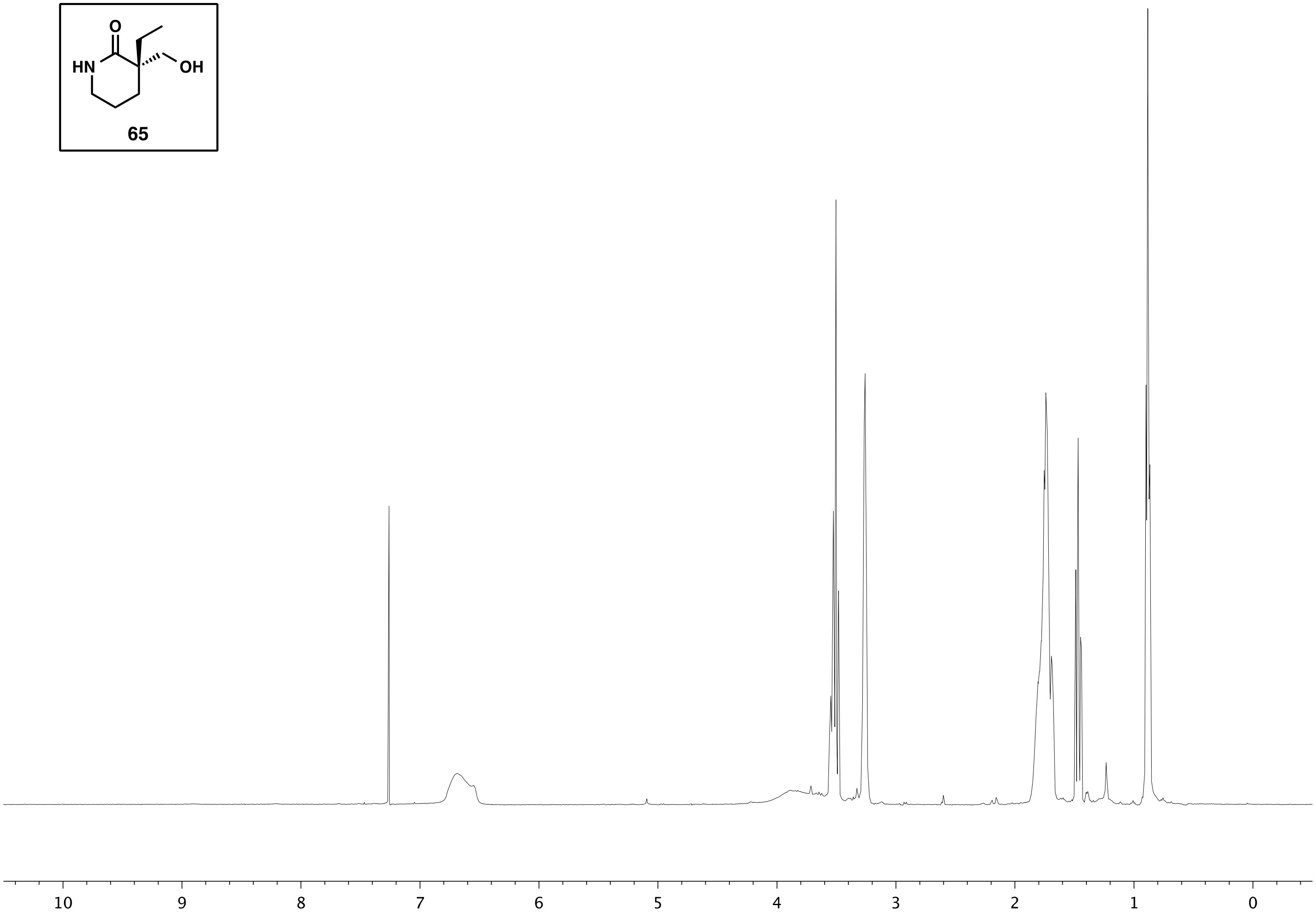

^{1}H NMR (500 MHz, $CDCl_3$) of compound **65**.

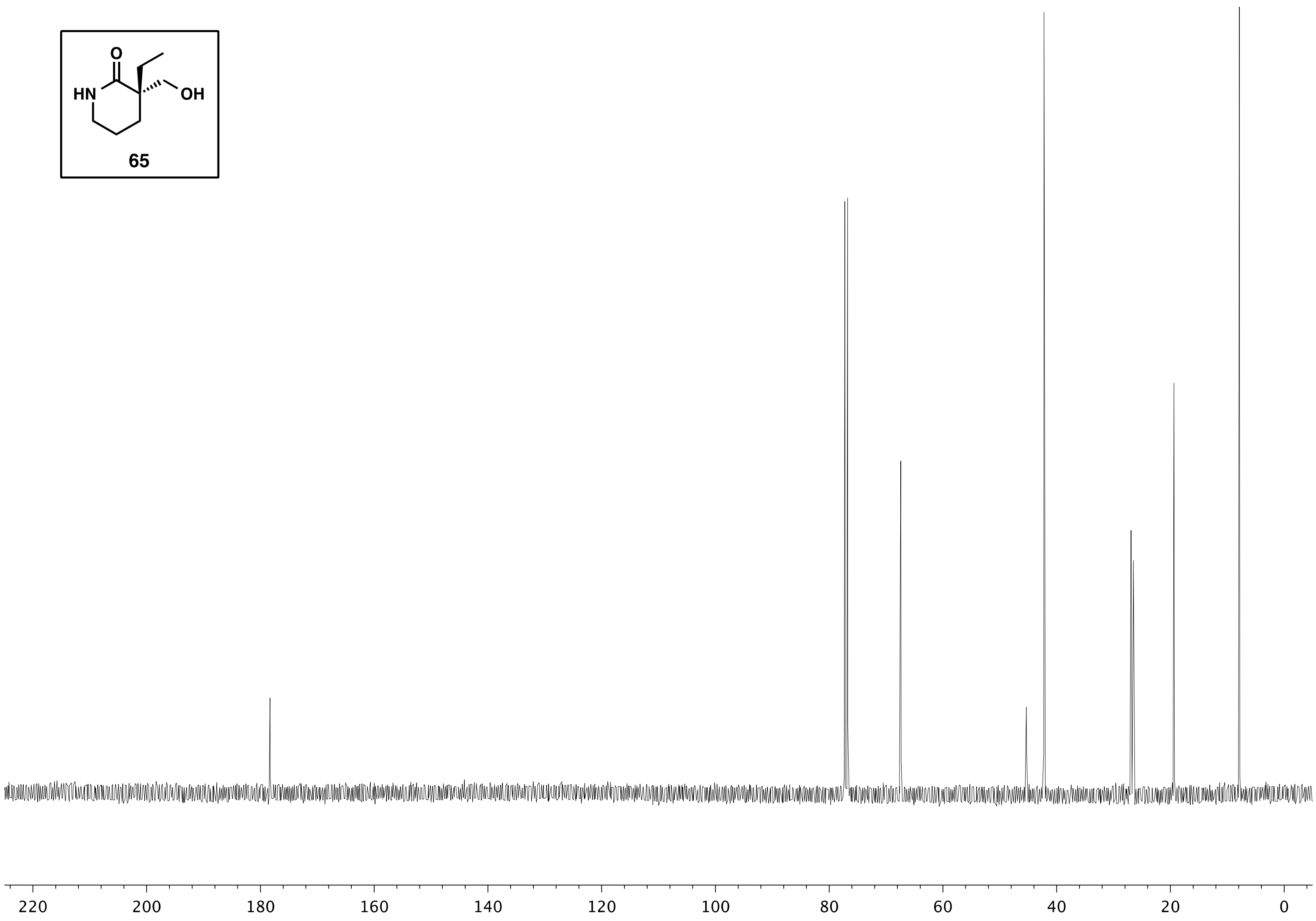

^{13}C NMR (126 MHz, $CDCl_3$) of compound **65**.

X-Ray Structure Determination

Low-temperature diffraction data (ϕ-and ω-scans) were collected on a Bruker Kappa diffractometer coupled to a Apex II CCD detector with graphite monochromated Mo K_α radiation (λ = 0.71073 Å) for the structure of compound **64** and (±)-**65**. The structure was solved by direct methods using SHELXS[1] and refined against F^2 on all data by full-matrix least squares with SHELXL-2013[2] using established refinement techniques.[3] All non-hydrogen atoms were refined anisotropically. Unless otherwise noted, all hydrogen atoms were included into the model at geometrically calculated positions and refined using a riding model. The isotropic displacement parameters of all hydrogen atoms were fixed to 1.2 times the *U* value of the atoms they are linked to (1.5 times for methyl groups).

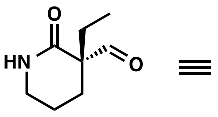

64

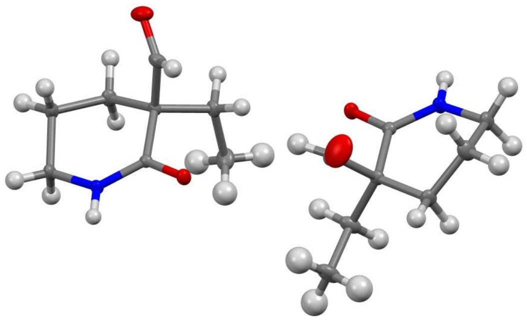

Compound **64** crystallizes in the monoclinic space group $P2_1$ with two molecules in the asymmetric unit. The coordinates for the hydrogen atoms bound to N1 and N2 were located in the difference Fourier synthesis and refined semi-freely with the help of a distance restraint. The N-H distances were restrained to be 0.88(4) Å. The absolute configuration was determined during the synthetic procedure and is consistent with the diffraction data. Bayesian statistics P2(true) 0.977, P3(true).... 0.680, P3(rac-twin) 0.304, P3(false) .. 0.016.[4]

---

[1] Sheldrick, G. M. *Acta Cryst.* **1990**, A46, 467-473.
[2] Sheldrick, G. M. *Acta Cryst.* **2008**, A64, 112-122.
[3] Müller, P. *Crystallography Reviews* **2009**, *15*, 57-83.
[4] Hoft,, R. Straver, L, Spek, A. *J. Appl. Cryst.* **2008**, *41*, 96-103.

Table 1: Crystal data and structure refinement for **64**.

Identification code	A14103	
CCDC Deposition Number	1000826	
Empirical formula	C8 H13 N O2	
Formula weight	155.19	
Temperature	100(2) K	
Wavelength	0.71073 Å	
Crystal system	Monoclinic	
Space group	P 21	
Unit cell dimensions	a = 8.1906(13) Å	α = 90°.
	b = 10.4809(17) Å	β = 110.678(7)°.
	c = 10.3279(16) Å	Y = 90°.
Volume	829.5(2) Å^3	
Z	4	
Density (calculated)	1.243 Mg/m^3	
Absorption coefficient	0.089 mm^{-1}	
F(000)	336	
Crystal size	0.700 x 0.070 x 0.050 mm^3	
Theta range for data collection	2.108 to 36.313°.	
Index ranges	-13<=h<=13, -17<=k<=17, -17<=l<=17	
Reflections collected	39923	
Independent reflections	8043 [R(int) = 0.0504]	
Completeness to theta = 25.242°	100.0 %	
Absorption correction	Semi-empirical from equivalents	
Max. and min. transmission	0.7474 and 0.6748	
Refinement method	Full-matrix least-squares on F^2	
Data / restraints / parameters	8043 / 3 / 207	
Goodness-of-fit on F^2	1.034	
Final R indices [I>2sigma(I)]	R1 = 0.0436, wR2 = 0.1030	
R indices (all data)	R1 = 0.0578, wR2 = 0.1104	

Absolute structure parameter	0.0(4)
Extinction coefficient	n/a
Largest diff. peak and hole	0.439 and -0.239 e.Å^{-3}

Table 2: Atomic coordinates (x 10^4) and equivalent isotropic displacement parameters (Å^2 x 10^3) for **64**. U(eq) is defined as one third of the trace of the orthogonalized U^{ij} tensor.

	x	y	z	U(eq)
C(1)	5129(2)	2251(1)	3426(1)	11(1)
O(1)	4972(1)	2819(1)	2328(1)	15(1)
C(2)	6748(2)	1423(1)	4116(1)	12(1)
C(6)	6594(2)	318(1)	3110(2)	17(1)
O(2)	7134(2)	-744(1)	3455(1)	25(1)
C(7)	8414(2)	2137(1)	4138(1)	16(1)
C(8)	8695(2)	3426(2)	4864(2)	25(1)
C(3)	6914(2)	931(1)	5548(1)	17(1)
C(4)	5150(2)	517(1)	5581(2)	19(1)
C(5)	3940(2)	1663(1)	5263(2)	18(1)
N(1)	3887(1)	2283(1)	3982(1)	13(1)
C(11)	10512(2)	4123(1)	1308(1)	12(1)
O(3)	10666(1)	3510(1)	2374(1)	16(1)
C(12)	8793(2)	4826(1)	554(1)	14(1)
C(16)	7434(2)	3755(2)	91(2)	24(1)
O(4)	6589(2)	3526(2)	-1097(2)	47(1)
C(17)	8355(2)	5646(2)	1638(2)	23(1)
C(18)	6550(2)	6260(2)	1077(2)	30(1)
C(13)	8826(2)	5624(2)	-676(2)	20(1)
C(14)	9870(2)	4978(2)	-1451(2)	24(1)
C(15)	11737(2)	4807(2)	-482(2)	21(1)
N(2)	11786(1)	4161(1)	787(1)	16(1)

Table 3: Bond lengths [Å] and angles [°] for **64**.

C(1)-O(1)	1.2460(15)

C(1)-N(1)	1.3336(16)
C(1)-C(2)	1.5334(17)
C(2)-C(3)	1.5262(19)
C(2)-C(6)	1.5311(19)
C(2)-C(7)	1.5497(18)
C(6)-O(2)	1.2037(18)
C(6)-H(6)	0.9500
C(7)-C(8)	1.523(2)
C(7)-H(7A)	0.9900
C(7)-H(7B)	0.9900
C(8)-H(8A)	0.9800
C(8)-H(8B)	0.9800
C(8)-H(8C)	0.9800
C(3)-C(4)	1.5208(19)
C(3)-H(3A)	0.9900
C(3)-H(3B)	0.9900
C(4)-C(5)	1.5176(19)
C(4)-H(4A)	0.9900
C(4)-H(4B)	0.9900
C(5)-N(1)	1.4617(17)
C(5)-H(5A)	0.9900
C(5)-H(5B)	0.9900
N(1)-H(1N)	0.901(17)
C(11)-O(3)	1.2420(16)
C(11)-N(2)	1.3334(17)
C(11)-C(12)	1.5352(17)
C(12)-C(13)	1.5286(19)
C(12)-C(16)	1.533(2)
C(12)-C(17)	1.552(2)
C(16)-O(4)	1.202(2)
C(16)-H(16)	0.9500
C(17)-C(18)	1.526(2)
C(17)-H(17A)	0.9900
C(17)-H(17B)	0.9900
C(18)-H(18A)	0.9800
C(18)-H(18B)	0.9800
C(18)-H(18C)	0.9800
C(13)-C(14)	1.521(2)
C(13)-H(13A)	0.9900
C(13)-H(13B)	0.9900

C(14)-C(15)	1.514(2)
C(14)-H(14A)	0.9900
C(14)-H(14B)	0.9900
C(15)-N(2)	1.4625(18)
C(15)-H(15A)	0.9900
C(15)-H(15B)	0.9900
N(2)-H(2N)	0.851(17)

O(1)-C(1)-N(1)	122.04(11)
O(1)-C(1)-C(2)	119.17(11)
N(1)-C(1)-C(2)	118.69(11)
C(3)-C(2)-C(6)	111.08(11)
C(3)-C(2)-C(1)	114.09(10)
C(6)-C(2)-C(1)	105.21(10)
C(3)-C(2)-C(7)	111.85(10)
C(6)-C(2)-C(7)	103.66(11)
C(1)-C(2)-C(7)	110.25(10)
O(2)-C(6)-C(2)	124.42(13)
O(2)-C(6)-H(6)	117.8
C(2)-C(6)-H(6)	117.8
C(8)-C(7)-C(2)	114.49(12)
C(8)-C(7)-H(7A)	108.6
C(2)-C(7)-H(7A)	108.6
C(8)-C(7)-H(7B)	108.6
C(2)-C(7)-H(7B)	108.6
H(7A)-C(7)-H(7B)	107.6
C(7)-C(8)-H(8A)	109.5
C(7)-C(8)-H(8B)	109.5
H(8A)-C(8)-H(8B)	109.5
C(7)-C(8)-H(8C)	109.5
H(8A)-C(8)-H(8C)	109.5
H(8B)-C(8)-H(8C)	109.5
C(4)-C(3)-C(2)	110.99(10)
C(4)-C(3)-H(3A)	109.4
C(2)-C(3)-H(3A)	109.4
C(4)-C(3)-H(3B)	109.4
C(2)-C(3)-H(3B)	109.4
H(3A)-C(3)-H(3B)	108.0
C(5)-C(4)-C(3)	108.79(11)
C(5)-C(4)-H(4A)	109.9

C(3)-C(4)-H(4A)	109.9
C(5)-C(4)-H(4B)	109.9
C(3)-C(4)-H(4B)	109.9
H(4A)-C(4)-H(4B)	108.3
N(1)-C(5)-C(4)	110.86(11)
N(1)-C(5)-H(5A)	109.5
C(4)-C(5)-H(5A)	109.5
N(1)-C(5)-H(5B)	109.5
C(4)-C(5)-H(5B)	109.5
H(5A)-C(5)-H(5B)	108.1
C(1)-N(1)-C(5)	126.55(11)
C(1)-N(1)-H(1N)	115.4(13)
C(5)-N(1)-H(1N)	118.0(13)
O(3)-C(11)-N(2)	121.64(11)
O(3)-C(11)-C(12)	119.05(11)
N(2)-C(11)-C(12)	119.30(11)
C(13)-C(12)-C(16)	111.43(11)
C(13)-C(12)-C(11)	113.70(11)
C(16)-C(12)-C(11)	103.98(11)
C(13)-C(12)-C(17)	111.89(12)
C(16)-C(12)-C(17)	107.65(12)
C(11)-C(12)-C(17)	107.73(10)
O(4)-C(16)-C(12)	123.93(15)
O(4)-C(16)-H(16)	118.0
C(12)-C(16)-H(16)	118.0
C(18)-C(17)-C(12)	113.55(12)
C(18)-C(17)-H(17A)	108.9
C(12)-C(17)-H(17A)	108.9
C(18)-C(17)-H(17B)	108.9
C(12)-C(17)-H(17B)	108.9
H(17A)-C(17)-H(17B)	107.7
C(17)-C(18)-H(18A)	109.5
C(17)-C(18)-H(18B)	109.5
H(18A)-C(18)-H(18B)	109.5
C(17)-C(18)-H(18C)	109.5
H(18A)-C(18)-H(18C)	109.5
H(18B)-C(18)-H(18C)	109.5
C(14)-C(13)-C(12)	111.85(12)
C(14)-C(13)-H(13A)	109.2
C(12)-C(13)-H(13A)	109.2

C(14)-C(13)-H(13B)	109.2
C(12)-C(13)-H(13B)	109.2
H(13A)-C(13)-H(13B)	107.9
C(15)-C(14)-C(13)	109.24(13)
C(15)-C(14)-H(14A)	109.8
C(13)-C(14)-H(14A)	109.8
C(15)-C(14)-H(14B)	109.8
C(13)-C(14)-H(14B)	109.8
H(14A)-C(14)-H(14B)	108.3
N(2)-C(15)-C(14)	110.44(12)
N(2)-C(15)-H(15A)	109.6
C(14)-C(15)-H(15A)	109.6
N(2)-C(15)-H(15B)	109.6
C(14)-C(15)-H(15B)	109.6
H(15A)-C(15)-H(15B)	108.1
C(11)-N(2)-C(15)	126.69(11)
C(11)-N(2)-H(2N)	115.7(14)
C(15)-N(2)-H(2N)	117.6(14)

Table 4: Anisotropic displacement parameters (Å^{2}x 10^3) for **64**. The anisotropic displacement factor exponent takes the form: -2p^2[h^2 a*^{2}U^{11} + ... + 2 h k a* b* U^{12}]

	U^{11}	U^{22}	U^{33}	U^{23}	U^{13}	U^{12}
C(1)	9(1)	11(1)	11(1)	0(1)	2(1)	0(1)
O(1)	14(1)	18(1)	13(1)	4(1)	5(1)	2(1)
C(2)	9(1)	14(1)	12(1)	1(1)	4(1)	1(1)
C(6)	13(1)	19(1)	19(1)	-4(1)	5(1)	2(1)
O(2)	21(1)	17(1)	36(1)	-3(1)	7(1)	3(1)
C(7)	9(1)	20(1)	18(1)	2(1)	5(1)	-1(1)
C(8)	23(1)	26(1)	26(1)	-7(1)	8(1)	-11(1)
C(3)	13(1)	22(1)	14(1)	7(1)	3(1)	4(1)
C(4)	18(1)	18(1)	23(1)	9(1)	11(1)	4(1)
C(5)	18(1)	20(1)	21(1)	8(1)	12(1)	5(1)
N(1)	11(1)	15(1)	15(1)	4(1)	6(1)	4(1)
C(11)	11(1)	12(1)	12(1)	0(1)	3(1)	0(1)
O(3)	14(1)	20(1)	14(1)	6(1)	5(1)	4(1)

C(12)	11(1)	16(1)	13(1)	4(1)	3(1)	3(1)
C(16)	14(1)	24(1)	30(1)	7(1)	1(1)	-2(1)
O(4)	34(1)	52(1)	37(1)	-1(1)	-7(1)	-20(1)
C(17)	20(1)	30(1)	17(1)	2(1)	5(1)	11(1)
C(18)	25(1)	39(1)	28(1)	7(1)	11(1)	18(1)
C(13)	18(1)	22(1)	18(1)	9(1)	7(1)	6(1)
C(14)	25(1)	31(1)	16(1)	8(1)	8(1)	7(1)
C(15)	21(1)	26(1)	21(1)	11(1)	12(1)	6(1)
N(2)	12(1)	19(1)	16(1)	6(1)	6(1)	4(1)

Table 5: Hydrogen coordinates (x 10^4) and isotropic displacement parameters (Å^2 x 10^3) for **64**.

	x	y	z	U(eq)
H(6)	6035	481	2149	21
H(7A)	9440	1590	4603	19
H(7B)	8351	2267	3172	19
H(8A)	7744	4005	4359	38
H(8B)	9810	3787	4891	38
H(8C)	8712	3315	5811	38
H(3A)	7729	199	5795	20
H(3B)	7401	1612	6241	20
H(4A)	4651	-162	4887	22
H(4B)	5288	172	6506	22
H(5A)	2750	1385	5174	22
H(5B)	4350	2280	6037	22
H(1N)	2940(20)	2750(20)	3510(20)	16
H(16)	7250	3243	786	29
H(17A)	8425	5102	2439	27
H(17B)	9243	6327	1974	27
H(18A)	6455	6781	265	45
H(18B)	6381	6801	1793	45
H(18C)	5656	5591	812	45
H(13A)	7615	5763	-1318	23
H(13B)	9345	6468	-340	23
H(14A)	9351	4138	-1802	28

H(14B)	9842	5508	-2252	28
H(15A)	12388	4297	-946	25
H(15B)	12309	5651	-249	25
H(2N)	12730(20)	3790(20)	1260(20)	19

Table 6: Hydrogen bonds for **64** [Å and °].

D-H...A	d(D-H)	d(H...A)	d(D...A)	<(DHA)
C(5)-H(5B)...O(2)#1	0.99	2.55	3.277(2)	130.3
N(1)-H(1N)...O(3)#2	0.901(17)	1.984(17)	2.8771(15)	171(2)
N(2)-H(2N)...O(1)#3	0.851(17)	2.047(17)	2.8963(15)	177(2)

Symmetry transformations used to generate equivalent atoms:

#1 -x+1,y+1/2,-z+1 #2 x-1,y,z #3 x+1,y,z

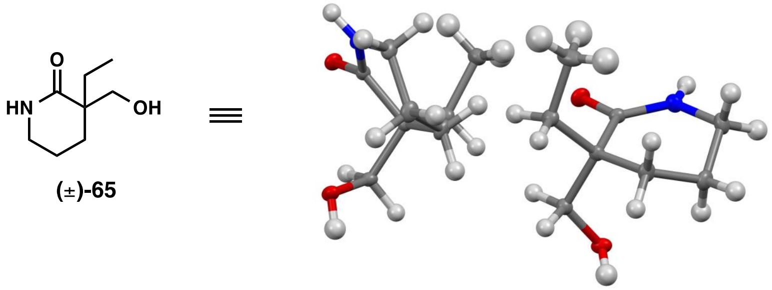

Compound (±)-**65** crystallizes in the triclinic space group $P\bar{1}$ with two molecules in the asymmetric unit. The structure was refined with two twin domains related by a 2-fold axis, twin law (-100/0-10/001). The twin ratio refined to 0.1275(16):0.8725(16). The highest electron density maxima are located on the bonds between atoms.

Table 7: Crystal data and structure refinement for (±)-**65**.

Identification code	A14199	
CCDC Deposition Number	1002339	
Empirical formula	C8 H15 N O2	
Formula weight	157.21	
Temperature	100(2) K	
Wavelength	0.71073 Å	
Crystal system	Triclinic	
Space group	P -1	
Unit cell dimensions	a = 6.0486(5) Å	α = 89.349(5)°.
	b = 11.6947(9) Å	β = 88.952(5)°.
	c = 11.8938(9) Å	γ = 84.996(5)°.
Volume	837.94(11) $Å^3$	

Z	4
Density (calculated)	1.246 Mg/m^3
Absorption coefficient	0.089 mm^{-1}
F(000)	344
Crystal size	0.250 x 0.200 x 0.150 mm^3
Theta range for data collection	1.713 to 35.630°.
Index ranges	-9<=h<=9, -19<=k<=19, -19<=l<=19
Reflections collected	48695
Independent reflections	7714 [R(int) = 0.0550]
Completeness to theta = 25.242°	100.0 %
Absorption correction	Semi-empirical from equivalents
Max. and min. transmission	0.7471 and 0.6469
Refinement method	Full-matrix least-squares on F^2
Data / restraints / parameters	7714 / 4 / 214
Goodness-of-fit on F^2	1.078
Final R indices [I>2sigma(I)]	R1 = 0.0793, wR2 = 0.2239
R indices (all data)	R1 = 0.1022, wR2 = 0.2511
Extinction coefficient	n/a
Largest diff. peak and hole	0.709 and -0.413 e.$Å^{-3}$

Table 8: Atomic coordinates (x 10^4) and equivalent isotropic displacement parameters ($Å^2$ x 10^3) for (±)-**65**. U(eq) is defined as one third of the trace of the orthogonalized U^{ij} tensor.

	x	y	z	U(eq)
N(1)	5794(3)	6168(2)	5728(2)	17(1)
C(1)	5529(3)	5411(2)	6563(2)	14(1)
O(1)	6927(2)	4579(1)	6721(1)	19(1)
C(2)	3485(3)	5561(2)	7352(2)	13(1)
C(3)	1921(3)	6634(2)	7084(2)	18(1)
C(6)	2270(3)	4459(2)	7288(2)	16(1)
O(2)	1294(2)	4312(1)	6233(1)	18(1)
C(7)	4304(3)	5600(2)	8571(2)	17(1)
C(8)	5917(4)	6501(2)	8764(2)	27(1)
C(4)	1908(3)	6950(2)	5834(2)	19(1)
C(5)	4248(4)	7153(2)	5440(2)	20(1)
N(101)	11065(3)	1082(1)	9237(1)	16(1)
C(101)	10787(3)	325(2)	8443(2)	13(1)
O(101)	12055(2)	-576(1)	8353(1)	17(1)
C(102)	8897(3)	540(2)	7617(2)	13(1)
C(103)	7567(3)	1713(2)	7772(2)	18(1)
C(106)	7405(3)	-457(2)	7767(2)	15(1)
O(102)	6312(2)	-464(1)	8840(1)	17(1)
C(107)	9890(3)	448(2)	6410(2)	16(1)
C(108)	11805(3)	1187(2)	6147(2)	21(1)
C(104)	7387(4)	2097(2)	8991(2)	20(1)
C(105)	9683(4)	2151(2)	9448(2)	19(1)

Table 9: Bond lengths [Å] and angles [°] for (±)-**65.**

N(1)-C(1)	1.340(3)
N(1)-C(5)	1.461(3)
N(1)-H(1N)	0.869(17)
C(1)-O(1)	1.247(2)
C(1)-C(2)	1.537(2)
C(2)-C(3)	1.538(2)
C(2)-C(6)	1.542(3)

C(2)-C(7)	1.542(3)
C(3)-C(4)	1.528(3)
C(3)-H(3A)	0.9900
C(3)-H(3B)	0.9900
C(6)-O(2)	1.415(2)
C(6)-H(6A)	0.9900
C(6)-H(6B)	0.9900
O(2)-H(2O)	0.820(18)
C(7)-C(8)	1.520(3)
C(7)-H(7A)	0.9900
C(7)-H(7B)	0.9900
C(8)-H(8A)	0.9800
C(8)-H(8B)	0.9800
C(8)-H(8C)	0.9800
C(4)-C(5)	1.520(3)
C(4)-H(4A)	0.9900
C(4)-H(4B)	0.9900
C(5)-H(5A)	0.9900
C(5)-H(5B)	0.9900
N(101)-C(101)	1.324(2)
N(101)-C(105)	1.464(2)
N(101)-H(01N)	0.880(17)
C(101)-O(101)	1.252(2)
C(101)-C(102)	1.524(2)
C(102)-C(103)	1.539(2)
C(102)-C(106)	1.543(3)
C(102)-C(107)	1.547(3)
C(103)-C(104)	1.522(3)
C(103)-H(10A)	0.9900
C(103)-H(10B)	0.9900
C(106)-O(102)	1.426(2)
C(106)-H(10C)	0.9900
C(106)-H(10D)	0.9900
O(102)-H(02O)	0.822(18)
C(107)-C(108)	1.531(3)
C(107)-H(10E)	0.9900
C(107)-H(10F)	0.9900
C(108)-H(10G)	0.9800
C(108)-H(10H)	0.9800
C(108)-H(10I)	0.9800

C(104)-C(105)	1.507(3)
C(104)-H(10J)	0.9900
C(104)-H(10K)	0.9900
C(105)-H(10L)	0.9900
C(105)-H(10M)	0.9900
C(1)-N(1)-C(5)	126.10(16)
C(1)-N(1)-H(1N)	121(2)
C(5)-N(1)-H(1N)	113(2)
O(1)-C(1)-N(1)	121.07(17)
O(1)-C(1)-C(2)	118.75(16)
N(1)-C(1)-C(2)	120.18(15)
C(1)-C(2)-C(3)	113.54(15)
C(1)-C(2)-C(6)	107.54(14)
C(3)-C(2)-C(6)	111.31(15)
C(1)-C(2)-C(7)	108.13(14)
C(3)-C(2)-C(7)	110.52(15)
C(6)-C(2)-C(7)	105.42(15)
C(4)-C(3)-C(2)	112.97(15)
C(4)-C(3)-H(3A)	109.0
C(2)-C(3)-H(3A)	109.0
C(4)-C(3)-H(3B)	109.0
C(2)-C(3)-H(3B)	109.0
H(3A)-C(3)-H(3B)	107.8
O(2)-C(6)-C(2)	113.33(15)
O(2)-C(6)-H(6A)	108.9
C(2)-C(6)-H(6A)	108.9
O(2)-C(6)-H(6B)	108.9
C(2)-C(6)-H(6B)	108.9
H(6A)-C(6)-H(6B)	107.7
C(6)-O(2)-H(2O)	109(3)
C(8)-C(7)-C(2)	114.14(17)
C(8)-C(7)-H(7A)	108.7
C(2)-C(7)-H(7A)	108.7
C(8)-C(7)-H(7B)	108.7
C(2)-C(7)-H(7B)	108.7
H(7A)-C(7)-H(7B)	107.6
C(7)-C(8)-H(8A)	109.5
C(7)-C(8)-H(8B)	109.5
H(8A)-C(8)-H(8B)	109.5

C(7)-C(8)-H(8C)	109.5
H(8A)-C(8)-H(8C)	109.5
H(8B)-C(8)-H(8C)	109.5
C(5)-C(4)-C(3)	109.48(17)
C(5)-C(4)-H(4A)	109.8
C(3)-C(4)-H(4A)	109.8
C(5)-C(4)-H(4B)	109.8
C(3)-C(4)-H(4B)	109.8
H(4A)-C(4)-H(4B)	108.2
N(1)-C(5)-C(4)	110.39(16)
N(1)-C(5)-H(5A)	109.6
C(4)-C(5)-H(5A)	109.6
N(1)-C(5)-H(5B)	109.6
C(4)-C(5)-H(5B)	109.6
H(5A)-C(5)-H(5B)	108.1
C(101)-N(101)-C(105)	126.71(16)
C(101)-N(101)-H(01N)	117(2)
C(105)-N(101)-H(01N)	116(2)
O(101)-C(101)-N(101)	121.24(16)
O(101)-C(101)-C(102)	118.64(16)
N(101)-C(101)-C(102)	120.12(15)
C(101)-C(102)-C(103)	113.21(15)
C(101)-C(102)-C(106)	106.96(14)
C(103)-C(102)-C(106)	111.53(15)
C(101)-C(102)-C(107)	108.21(14)
C(103)-C(102)-C(107)	110.07(15)
C(106)-C(102)-C(107)	106.57(14)
C(104)-C(103)-C(102)	113.37(15)
C(104)-C(103)-H(10A)	108.9
C(102)-C(103)-H(10A)	108.9
C(104)-C(103)-H(10B)	108.9
C(102)-C(103)-H(10B)	108.9
H(10A)-C(103)-H(10B)	107.7
O(102)-C(106)-C(102)	113.08(15)
O(102)-C(106)-H(10C)	109.0
C(102)-C(106)-H(10C)	109.0
O(102)-C(106)-H(10D)	109.0
C(102)-C(106)-H(10D)	109.0
H(10C)-C(106)-H(10D)	107.8
C(106)-O(102)-H(02O)	104(3)

C(108)-C(107)-C(102)	115.97(16)
C(108)-C(107)-H(10E)	108.3
C(102)-C(107)-H(10E)	108.3
C(108)-C(107)-H(10F)	108.3
C(102)-C(107)-H(10F)	108.3
H(10E)-C(107)-H(10F)	107.4
C(107)-C(108)-H(10G)	109.5
C(107)-C(108)-H(10H)	109.5
H(10G)-C(108)-H(10H)	109.5
C(107)-C(108)-H(10I)	109.5
H(10G)-C(108)-H(10I)	109.5
H(10H)-C(108)-H(10I)	109.5
C(105)-C(104)-C(103)	109.30(17)
C(105)-C(104)-H(10J)	109.8
C(103)-C(104)-H(10J)	109.8
C(105)-C(104)-H(10K)	109.8
C(103)-C(104)-H(10K)	109.8
H(10J)-C(104)-H(10K)	108.3
N(101)-C(105)-C(104)	111.07(16)
N(101)-C(105)-H(10L)	109.4
C(104)-C(105)-H(10L)	109.4
N(101)-C(105)-H(10M)	109.4
C(104)-C(105)-H(10M)	109.4
H(10L)-C(105)-H(10M)	108.0

Symmetry transformations used to generate equivalent atoms:

Table 10: Anisotropic displacement parameters ($Å^2$ x 10^3) for (±)-**65**. The anisotropic displacement factor exponent takes the form: $-2p^2[h^2 a^{*2}U^{11} + ... + 2 h k a^* b^* U^{12}]$

	U^{11}	U^{22}	U^{33}	U^{23}	U^{13}	U^{12}
N(1)	16(1)	17(1)	17(1)	0(1)	4(1)	0(1)
C(1)	12(1)	13(1)	16(1)	-3(1)	1(1)	1(1)
O(1)	13(1)	19(1)	23(1)	-1(1)	1(1)	6(1)
C(2)	11(1)	12(1)	15(1)	-1(1)	2(1)	2(1)
C(3)	17(1)	15(1)	21(1)	0(1)	3(1)	6(1)
C(6)	15(1)	14(1)	18(1)	0(1)	1(1)	-1(1)

O(2)	12(1)	22(1)	19(1)	-5(1)	1(1)	-1(1)
C(7)	18(1)	19(1)	15(1)	-1(1)	0(1)	-1(1)
C(8)	26(1)	36(1)	21(1)	-10(1)	3(1)	-14(1)
C(4)	20(1)	15(1)	22(1)	2(1)	-3(1)	5(1)
C(5)	25(1)	15(1)	20(1)	3(1)	2(1)	0(1)
N(101)	16(1)	14(1)	16(1)	-1(1)	-4(1)	0(1)
C(101)	12(1)	11(1)	15(1)	0(1)	0(1)	1(1)
O(101)	12(1)	15(1)	22(1)	0(1)	-1(1)	4(1)
C(102)	13(1)	12(1)	14(1)	0(1)	-2(1)	2(1)
C(103)	20(1)	15(1)	19(1)	-2(1)	-5(1)	7(1)
C(106)	12(1)	15(1)	17(1)	-1(1)	-1(1)	-2(1)
O(102)	12(1)	24(1)	17(1)	2(1)	0(1)	-1(1)
C(107)	18(1)	17(1)	14(1)	-1(1)	1(1)	-2(1)
C(108)	20(1)	23(1)	20(1)	2(1)	-1(1)	-6(1)
C(104)	22(1)	15(1)	21(1)	-2(1)	0(1)	7(1)
C(105)	26(1)	12(1)	18(1)	-2(1)	-3(1)	1(1)

Table 11: Hydrogen coordinates (x 10^4) and isotropic displacement parameters (Å^2 x 10^3) for (±)-**65**.

	x	y	z	U(eq)
H(1N)	6870(40)	6050(30)	5240(20)	20
H(3A)	395	6497	7333	21
H(3B)	2382	7289	7516	21
H(6A)	3338	3788	7444	19
H(6B)	1096	4481	7881	19
H(2O)	-50(30)	4450(30)	6300(30)	27
H(7A)	3002	5752	9081	20
H(7B)	5034	4837	8773	20
H(8A)	7266	6324	8307	41
H(8B)	6301	6502	9561	41
H(8C)	5225	7259	8549	41
H(4A)	1357	6320	5398	23
H(4B)	903	7652	5709	23
H(5A)	4271	7279	4615	24
H(5B)	4714	7851	5800	24

H(01N)	12130(40)	910(30)	9720(20)	19
H(10A)	8287	2297	7320	22
H(10B)	6055	1670	7480	22
H(10C)	6274	-400	7173	18
H(10D)	8323	-1193	7668	18
H(02O)	5030(30)	-580(30)	8690(30)	26
H(10E)	10420	-365	6274	19
H(10F)	8687	659	5873	19
H(10G)	11282	2000	6225	31
H(10H)	12348	1044	5376	31
H(10I)	13012	991	6673	31
H(10J)	6554	2863	9038	24
H(10K)	6574	1549	9444	24
H(10L)	10384	2800	9088	23
H(10M)	9577	2290	10268	23

Table 12: Hydrogen bonds for (±)-**65** [Å and °].

D-H...A	d(D-H)	d(H...A)	d(D...A)	<(DHA)
N(1)-H(1N)...O(2)#1	0.869(17)	2.08(2)	2.926(2)	163(3)
O(2)-H(2O)...O(1)#2	0.820(18)	1.880(19)	2.686(2)	168(4)
C(4)-H(4A)...O(2)	0.99	2.54	3.168(3)	121.0
N(101)-H(01N)...O(102)#3	0.880(17)	2.01(2)	2.858(2)	161(3)
O(102)-H(02O)...O(101)#2	0.822(18)	1.849(19)	2.664(2)	171(4)
C(104)-H(10K)...O(102)	0.99	2.49	3.128(3)	122.0

Symmetry transformations used to generate equivalent atoms:

#1 -x+1,-y+1,-z+1 #2 x-1,y,z #3 -x+2,-y,-z+2
